# FPNuNet: a frequency-aware prompt-guided network for nuclear segmentation and classification in immunohistochemistry images

**DOI:** 10.1093/gigascience/giag091

**Published:** 2026-09-07

**Authors:** Lulu Qin, Zhigang Pei, Xudong He, Jiarui Zhou, Xianhong Xu, Zexuan Zhu

**Affiliations:** College of Computer Science and Software Engineering, Shenzhen University, Nanhai Avenue 3688, Nanshan District, Shenzhen 518060, China; Pathology Department, Chongqing University Jiangjin Hospital, Jinchaijing Road 93, Jiangjin District, Chongqing 402260‌, China; School of Artificial Intelligence, Shenzhen University, Nanhai Avenue 3688, Nanshan District, Shenzhen 518060, China; School of Biosciences, University of Birmingham, Edgbaston, Birmingham B15 2TT, UK; Department of Oncology, University of Oxford, Roosevelt Drive, Oxford OX3 7DQ, UK; School of Artificial Intelligence, Shenzhen University, Nanhai Avenue 3688, Nanshan District, Shenzhen 518060, China

## Abstract

**Background:**

Accurate nuclear segmentation and classification (NuSC) in immunohistochemistry (IHC)-stained whole-slide images is essential for reliable biomarker quantification in computational pathology. Although existing NuSC methods perform well on Hematoxylin and Eosin images, they frequently underperform on IHC-stained images owing to stain heterogeneity, low nuclear contrast, and scarce biomarker-specific annotations. Prior efforts based on stain normalization or cross-domain knowledge transfer have yielded only marginal improvements under these conditions. To address these limitations, we introduce FPNuNet, a frequency-aware prompt-guided network tailored for NuSC of IHC-stained images.

**Results:**

FPNuNet extracts multi-source features through 4 parallel encoders: a frozen SAM-based structural encoder and a frozen UNI-based semantic encoder—both adapted via lightweight discrete cosine transform-based prompt generators—together with a wavelet feature encoder and a multi-scale context encoder that capture complementary spatial and frequency-domain descriptors from the raw input. A discrete cosine transform-enabled frequency-aware fusion neck integrates all 4 feature streams with spectral enhancement, and 3 collaborative decoder branches jointly predict binary masks, horizontal–vertical vectors, and nuclear types. FPNuNet is evaluated on CD47-IHCNuSC, a dataset comprising 86 CD47-stained esophageal cancer patches with 18,483 manually annotated nuclei spanning 7 clinically meaningful subtypes. On CD47-IHCNuSC, FPNuNet achieves the best instance-segmentation and aggregate subtype-classification performance among all evaluated methods under challenging IHC staining conditions.

**Conclusions:**

These results demonstrate the effectiveness of frequency-aware prompt-guided feature learning for nuclear segmentation and classification in IHC-stained images and suggest that FPNuNet provides a promising framework for robust biomarker-oriented computational pathology analysis.

## Introduction

Automated histopathology is reshaping clinical workflows by enabling reproducible, scalable biomarker quantification, and large-cohort analysis [1, 2]. Robust nuclear segmentation and classification (NuSC) is central to these pipelines, underpinning cell-level readouts (e.g., counts, densities, and phenotypes) that inform diagnosis, prognosis, and treatment monitoring [3]. Yet clinical slide heterogeneity and lab-specific protocols demand NuSC models that remain accurate across staining variability and acquisition differences [4–6].

Deep learning has driven rapid advances in NuSC on Hematoxylin and Eosin (H&E) slides, enabled by sustained algorithmic innovation and the standardization of evaluation on widely adopted public benchmarks. Representative architectures such as HoVer-Net [7] unify pixel-wise masks with horizontal–vertical (HV) distance regression to split touching instances while enabling joint nuclear typing. TransUNet [8] combines a transformer encoder with a U-Net style decoder to fuse long-range dependencies with multi-scale detail. Triple U-Net [9] adds hematoxylin-aware pathways and progressive dense aggregation to stabilize boundaries and reduce type confusion. Swin-UNet [10] employs hierarchical windowed self-attention with shifted windows to capture multi-scale context efficiently. Collectively, these designs achieve reliable instance delineation and nuclear typing on H&E slides, yet they are optimized for H&E-specific stain appearance and morphology, which limits direct transfer to immunohistochemistry (IHC)-stained images [11].

IHC introduces heterogeneous 3,3$^\prime$-diaminobenzidine (DAB) deposition that alters local spectral characteristics and attenuates the hematoxylin counterstain, thereby blurring nuclear boundaries and reducing intra-nuclear contrast. Variation in antibody selection and staining protocols further reshapes tissue texture and class distribution, amplifying ambiguity among positive, weak-positive, and negative phenotypes. Together, these domain shifts produce characteristic errors—boundary leakage, over- or under-segmentation, and inconsistent type assignment—motivating the IHC-specific methods discussed below.

Recent IHC-oriented NuSC research has pursued 2 complementary strategies. The first learns directly on IHC with coupled objectives so that segmentation and phenotype or intensity estimation co-adapt within a single model. For example, IHC-Net [12] combines a fully convolutional network segmenter with watershed splitting and a classical ensemble for intensity scoring on estrogen receptor IHC. SRSA-Net [13] modernizes the triple-head design with a lightweight encoder, decoder-level self-attention, and stitching. PH-ScoreNet [14] uses dual branches and dual attention to predict IHC scores end-to-end for proliferating cell nuclear antigen. The second reduces the stain gap prior to segmentation through generative translation or morphology normalization. For instance, GeNSeg-Net [15] performs in-domain generative adversarial network (GAN) based enhancement followed by a lightweight U-Net and watershed, achieving strong cross-tissue transfer. NuclSeg [16] bridges from IHC to multiplex immunofluorescence images using semi-supervised stain deconvolution with multi-GAN fusion and thus depends on co-registered pairs. UPHGAN [17] translates IHC to virtual H&E with a U-Net–PatchGAN generator–discriminator trained with a Huber-augmented objective and then fuses masks from both domains. Trullo et al. [18] similarly translate IHC to virtual H&E to leverage H&E-specialized segmenters such as HoVer-Net, although gains for cross-modal models such as StarDist remain modest. ISC-GAN integrates CycleGAN with Mask R-CNN in a curriculum to adapt from labeled H&E to unlabeled IHC [19]. An alternative strategy treats IHC primarily as a supervision source rather than an inference modality, employing destain–restain registration to transfer IHC-derived labels back to H&E and thereby improve immune-cell recognition [20]. Despite tangible gains from task coupling, in-domain enhancement, and cross-stain supervision, progress remains constrained by the absence of publicly accessible, biomarker-specific IHC benchmarks with subtype-level NuSC labels and by the scarcity of architectures explicitly designed for heterogeneous IHC staining conditions.

A further obstacle is the paucity of high-quality, publicly available, and expertly annotated IHC datasets [3]. While established benchmarks such as CoNSeP [7], MoNuSAC [21], and PanNuke [22] have catalyzed advances in H&E-based NuSC, they do not account for IHC-specific complexity—heterogeneous DAB deposition, diminished nuclear contrast, and the absence of biomarker context. By contrast, IHC resources remain fragmented: many widely cited datasets are private or request-only [12, 14–16, 20, 23], and others are image-only test corpora without publicly released ground truth, with scoring available only via server-side submission [17, 18]. Even where labels exist, they are often restricted to binary positivity rather than per-nucleus subtypes and frequently lack standardized tiling/stitching protocols, complicating fair instance-level evaluation and cross-study replication [13, 17, 18]. In sum, the field still lacks publicly accessible, expert-verified IHC benchmarks with subtype-level annotations and standardized evaluation protocols, suitable for rigorous, reproducible NuSC on IHC.

To address the aforementioned issues and facilitate biomarker-aware NuSC under the severe stain variability of IHC, we present FPNuNet, a frequency-aware, prompt-guided network that employs 4 parallel encoders—a frozen segment anything model (SAM) structural encoder [24], a frozen UNI pathology semantic encoder [25], a wavelet feature encoder (WFE), and a multi-scale context encoder (MSCE)—and integrates their outputs through a discrete cosine transform (DCT)-enabled frequency-aware fusion neck, as shown in Fig. 1. FPNuNet is evaluated on IHC-stained images of a representative biomarker, the tumor-associated antigen *cluster of differentiation 47* (CD47). CD47 overexpression enables tumor cells to evade macrophage-mediated phagocytosis and is correlated with aggressive behavior and poor prognosis in esophageal carcinoma and other malignancies [26–28]. To support this task, we curate CD47-IHCNuSC, an expert-annotated benchmark for CD47-stained esophageal carcinoma. Experiments on CD47-IHCNuSC demonstrate that FPNuNet achieves strong instance-segmentation and subtype-classification performance under challenging IHC staining conditions. The main contributions are as follows:

- We propose FPNuNet, a frequency-aware, prompt-guided architecture for NuSC of IHC-stained images. FPNuNet comprises 4 parallel encoders (a frozen SAM structural encoder, a frozen UNI pathology encoder, WFE, and MSCE), a DCT-enabled prompt-guided frequency-aware enhancement (PFAE) fusion neck, and tri-branch decoders for simultaneous binary, HV, and type predictions.
- We release CD47-IHCNuSC, the first publicly available IHC dataset dedicated to CD47-stained esophageal cancer. The dataset comprises 86 high-resolution patches (18,483 nuclei across 7 clinically meaningful subtypes), annotated through a semi-automated, pretrained-model-assisted protocol with expert verification.
- Experiments on CD47-IHCNuSC show that FPNuNet achieves the highest AJI and bPQ among all evaluated methods trained on the same benchmark, with progressive ablation confirming complementary contributions from prompt adaptation, frequency-aware fusion, and decoder design.

**Figure 1:**
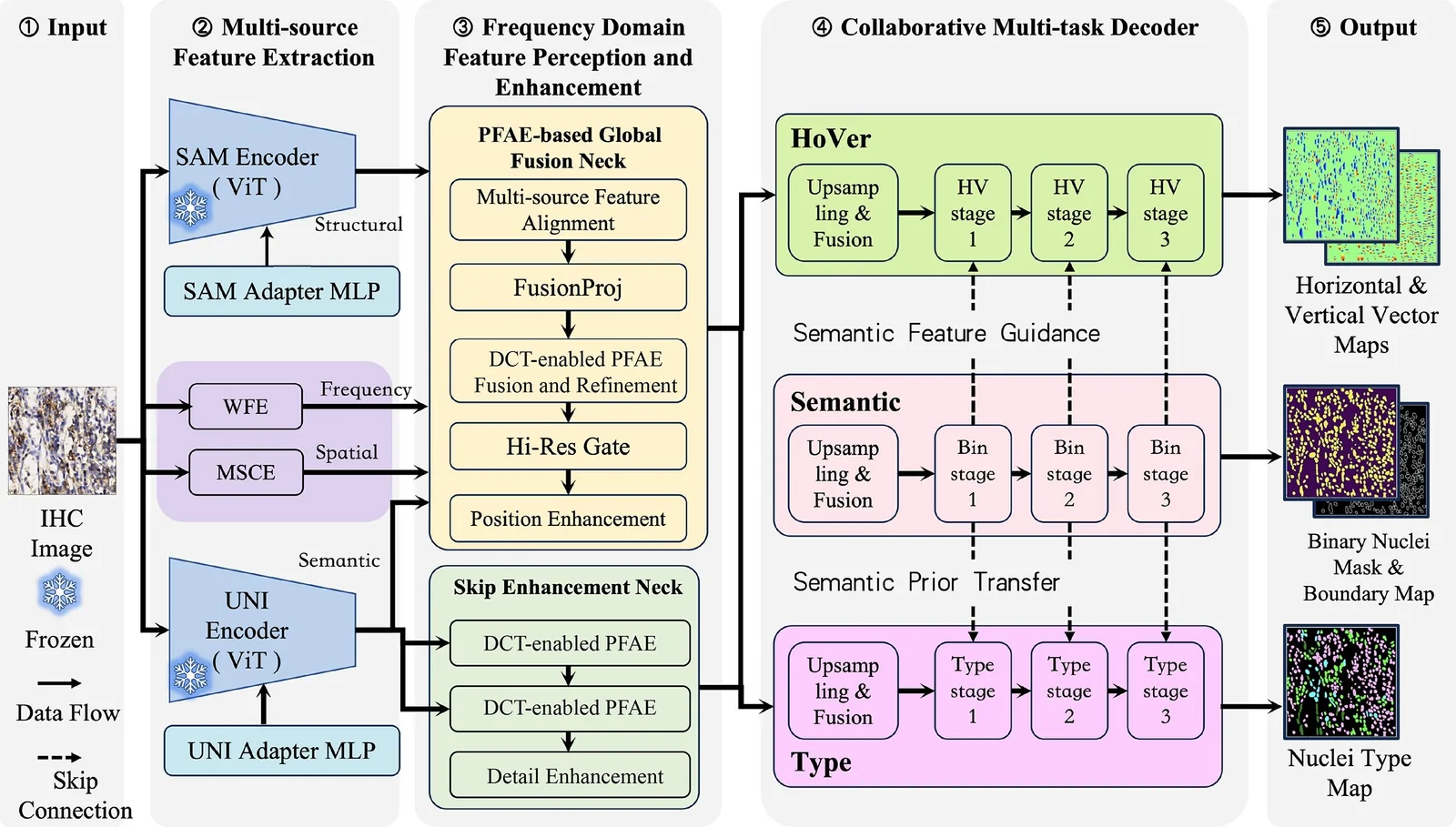
Overview of the FPNuNet architecture. RGB input patches are processed by a frozen SAM ViT-B encoder with DCT-based high-pass prompt adaptation, a frozen UNI ViT-L encoder with deep additive prompt injection, and lightweight trainable WFE and MSCE branches. A DCT-enabled 4-way PFAE fusion neck integrates all feature streams, while DCT-enabled skip necks refine UNI intermediate features. Three task-specific decoders—binary, HV, and type—are sequentially guided by binary decoder features through gated fusion, jointly predicting nuclei masks, boundaries, HV regression maps, and nuclear type labels.

## Materials and methods

### Data description

To enable rigorous evaluation of biomarker-aware NuSC under clinically realistic IHC conditions, we construct **CD47-IHCNuSC**, a new benchmark targeting CD47-stained esophageal cancer. Among publicly accessible resources, label granularity is typically limited to detection or binary positivity rather than fine-grained nuclear subtyping—for example, BCData [29] offers point detection with 2 classes, DeepLIIF [30] provides binary nuclear positivity labels without instance-level delineation, and the SRSA-Net TMA corpus [13] annotates nuclei with only 2 categories (positive/negative) on breast tissue microarray cores. Other efforts emphasize stain-domain robustness rather than subtype-level NuSC, and several influential works remain non-public or prioritize intensity scoring over subtype taxonomy [12, 23, 31]. **CD47-IHCNuSC** addresses these limitations by targeting esophageal tissue and the CD47 biomarker, providing expert-validated instance masks with 7 fine-grained, phenotype-aware nuclear subtypes under realistic IHC variability.

#### Construction, annotation, and quality control

All CD47-immunostained esophageal whole-slide images (WSIs) were processed using a DAB chromogen with hematoxylin counterstain and digitized at $0.25\, \mu$m/pixel (*40×*) on an Aperio AT2 scanner. A board-certified pathologist delineated tumor-enriched, morphologically diverse regions; patches with low tissue content were excluded. We sampled **86** non-overlapping *1024× 1024* patches (see Table 1 for image counts and class distributions). To preserve real-world appearance, no stain normalization was applied.

**Table 1:** Distribution of nuclear categories and image counts in the CD47-IHCNuSC dataset.

Nuclear category	Abbreviation	Train	Test	Total	Proportion
Positive-tumor	pTu	5,958	1,689	7,647	**41.37%**
Positive-immune	pIm	4,712	882	5,594	**30.27%**
Positive-others	pOth	17	8	25	0.14%
Negative-tumor	nTu	1,473	214	1,687	9.13%
Negative-immune	nIm	2,631	432	3,063	16.57%
Negative-stroma	nSt	410	54	464	2.51%
Negative-others	nOth	3	0	3	0.02%
Total nuclei	–	15,204	3,279	18,483	100%
Number of images	–	70	16	86	–

Abbreviation: P = positive, N = negative, Tu = tumor, Im = immune, St = stroma, Oth = others.

To generate instance-level and type-level annotations, a semi-automated human-in-the-loop pipeline was used, as shown in Fig. 2. Coarse nuclear masks were initially generated using HoverFast [32], a publicly available segmentation model that includes a variant pretrained for nuclear semantic segmentation on IHC-stained images. These preliminary masks were refined in QuPath through multiple rounds of split/merge editing and boundary correction. Trained scientists assigned preliminary subtypes; board-certified pathologists adjudicated ambiguities and harmonized criteria. Final annotations are released in standardized formats—OME-TIFF for images and mat for instance masks—to ensure reproducibility and compatibility with downstream pipelines.

**Figure 2:**
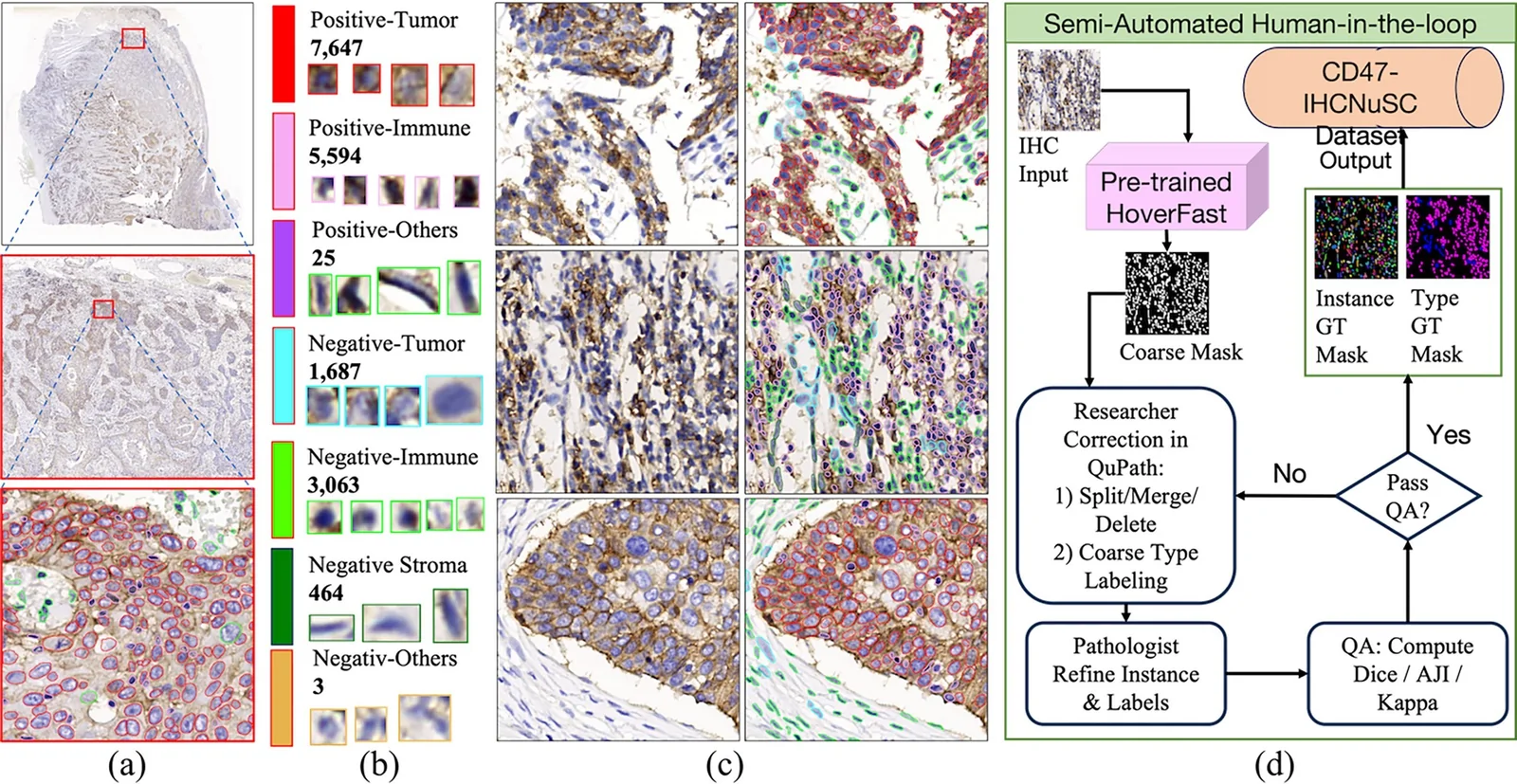
Overview of the CD47-IHCNuSC dataset and annotation workflow. (a) Representative CD47-stained WSIs, tumor-enriched regions, and extracted $1024{\times }1024$ patches. (b) Nuclear crops from 7 annotated subtypes, including biomarker-positive and biomarker-negative tumor, immune, stromal, and rare cells; border colors correspond to subtype labels. (c) Visualization of subtype-labeled nuclei overlaid on the original image. Colors match subtype labels shown in (b). (d) Semi-automated annotation pipeline: HoverFast-based pre-labeling, manual refinement in QuPath, and expert validation with quality assurance.

#### Statistics, benchmark characteristics, and availability

The final dataset comprises **18,483** quality-controlled nuclei assigned to **7** histologically and biomarker-informed subtypes spanning CD47-positive/negative tumor, immune, stromal, and rare/other categories (Table 1; Fig. 2). The distribution exhibits a pronounced long tail: positive-tumor and immune nuclei dominate, whereas rare categories (e.g., negative-stroma and negative-others) are sparsely represented. Beyond class imbalance, the benchmark captures 3 key challenges: (i) stain heterogeneity (DAB intensity and counterstain variability), (ii) nuclear crowding and adhesion in tumor regions, and (iii) shape and size diversity across tissue contexts. Collectively, these factors stress a model’s ability to jointly delineate instances and infer biomarker-sensitive types under real-world clinical heterogeneity.

Because 3 of the 7 annotated categories are either extremely rare or carry limited clinical specificity—pOth (0.14%, 25 instances) and nOth (0.02%, 3 instances) represent morphologically ambiguous nuclei, while nSt (2.51%, 464 instances) provides insufficient test-set representation ($n_{\mathrm{test}}\,{=}\,54$) for reliable per-class evaluation—they are merged into morphologically related categories before model training and evaluation: pOth is merged into pTu, and nSt is merged into nTu. The remaining negative-immune (nIm) and negative-others (nOth) categories are combined into a single **negative-others (nOth)** class, as both represent biomarker-negative non-tumor nuclei whose morphological distinction is unreliable at the annotation granularity of this study. All models therefore operate on **4 foreground classes** (pTu, pIm, nTu, nOth) plus background, and all evaluation is reported at this 4-class granularity.

CD47-IHCNuSC complements the current breast-centric datasets [12, 23, 29, 30] and multiplex tissue microarray (TMA) corpora [13] by broadening biomarker and organ coverage to esophageal IHC with fine-grained nuclear subtyping. The combination of biomarker specificity, fine-grained nuclear categorization, real-world stain variability, and expert-reviewed annotations makes CD47-IHCNuSC a challenging and representative benchmark. It provides a valuable resource for training and evaluating segmentation models under clinically realistic IHC conditions. In the following section, we evaluate FPNuNet and existing baselines on CD47-IHCNuSC to characterize its utility and difficulty as a benchmark for biomarker-aware NuSC.

### FPNuNet

As illustrated in Fig. 1, FPNuNet comprises 3 functional stages: (1) a multi-source feature extraction module with 4 parallel encoders for structural, semantic, spatial, and frequency-domain feature extraction, (2) DCT-enabled PFAE modules for multi-branch feature integration and spectral enhancement, and (3) multi-task decoders with hierarchical guidance for collaborative prediction. The 4 encoders consist of a frozen SAM ViT-B structural encoder and a frozen UNI ViT-L semantic encoder—both adapted through lightweight, input-dependent DCT-based prompt generators—together with WFE and MSCE branches, which extract complementary spatial and frequency-domain descriptors directly from the raw RGB input. All 4 feature streams are integrated within a 4-way DCT-enabled PFAE global fusion neck. The resulting representations are decoded through 3 task-specific branches—binary, HV regression, and nuclear type classification—that jointly produce binary masks, HV offset vectors for instance separation, and nuclear subtype maps, supporting joint learning for NuSC. Of the 394.8 M total parameters, only 5.4 M (1.37%) are trainable, demonstrating parameter-efficient foundation-model adaptation for histopathology nuclei analysis.

#### Multi-source feature extraction

As shown in Fig. 1, the multi-source feature extraction stage comprises 4 parallel encoders that independently extract complementary multi-domain, multi-scale representations from the raw input image. Two frozen pretrained encoder backbones provide high-level structural and semantic features, while 2 lightweight multi-domain feature encoders extract spatial and frequency-domain descriptors directly from the RGB input.

##### Structural encoder (SAM)

A pretrained SAM Vision Transformer (ViT)-B image encoder [24] serves as the structural feature backbone. The encoder backbone is frozen except for a trainable patch embedding projection (Conv2d, *3 → 768*, kernel *8 × 8*, stride 8; 148,224 parameters) and a lightweight high-pass prompt generator (49,488 parameters). The prompt generator operates in 2 branches: a DCT-based high-pass branch that applies a frequency-domain mask to suppress low-frequency content and reconstruct edge-sensitive features from the input image, and a patch-embedding branch that captures local structural cues. Both branches are fused through a shared bottleneck multi-layer perceptron (dimension 24) with per-block lightweight projections. For each of the 12 transformer blocks, the resulting token-wise prompt embedding is additively injected before the block, enabling deep, input-dependent visual prompt adaptation without modifying the pretrained attention weights. The SAM prompt encoder and mask decoder are not used; only the image encoder output at *16 × 16* spatial resolution is retained.

##### Semantic encoder (UNI)

A pretrained UNI ViT-L pathology foundation model [25] provides semantic embeddings. The UNI backbone is entirely frozen; domain adaptation is achieved through the same prompt generator architecture used for SAM, instantiated with a 1,024-dimensional embedding space and a bottleneck dimension of 32 (103,872 parameters). Token-wise additive prompts are injected into 12 of UNI’s 24 transformer layers (alternating layers 0, 2,..., 22), constituting deep visual prompt injection at half-depth granularity. Intermediate features from layers 3, 12, and 21 are extracted as skip features and reshaped to spatial resolutions of $32\, \times \, 32$, $64\, \times \, 64$, and $128\, \times \, 128$ for decoder use.

Both encoders share the same PromptGenerator class. Unlike low-rank adaptation [33], which applies input-agnostic weight perturbations, the prompt generator produces input-dependent, frequency-informed additive tokens, preserving the pretrained representation while injecting task-relevant boundary and texture cues.

##### Multi-domain feature encoders

The pretrained encoder backbones provide high-level structural and semantic representations, but their outputs are spatially coarse after repeated downsampling, and their feature statistics carry pretrained-domain bias that may not fully preserve the high-frequency textures and local contrast critical for nuclear boundary delineation in IHC images. To compensate, FPNuNet introduces 2 lightweight multi-domain feature encoders that operate directly on the raw RGB input, supplying complementary frequency-domain and spatial-domain descriptors that are orthogonal to the pretrained features.

As shown in Fig. 3a, WFE decomposes the input via Haar wavelet pooling convolution into 4 sub-bands—LL (low-frequency approximation), LH, HL, and HH (horizontal, vertical, and diagonal high-frequency detail)—which are concatenated along the channel dimension, upsampled, and fused back to the input resolution through a $3\, \times \, 3$ convolution followed by a $1\, \times \, 1$ projection (952 trainable parameters), producing a single full-resolution frequency descriptor $\mathbf {F}_{\mathrm{WFE}} \in \mathbb {R}^{8 \times 128 \times 128}$. Wavelet decomposition inherently supports multi-resolution analysis: it separates frequency components while preserving spatial localization, enabling the model to perceive nuclear boundaries, chromogen deposition textures, and other high-frequency structural cues that are often obscured by the stain heterogeneity and weak boundary contrast characteristic of IHC images.

**Figure 3:**
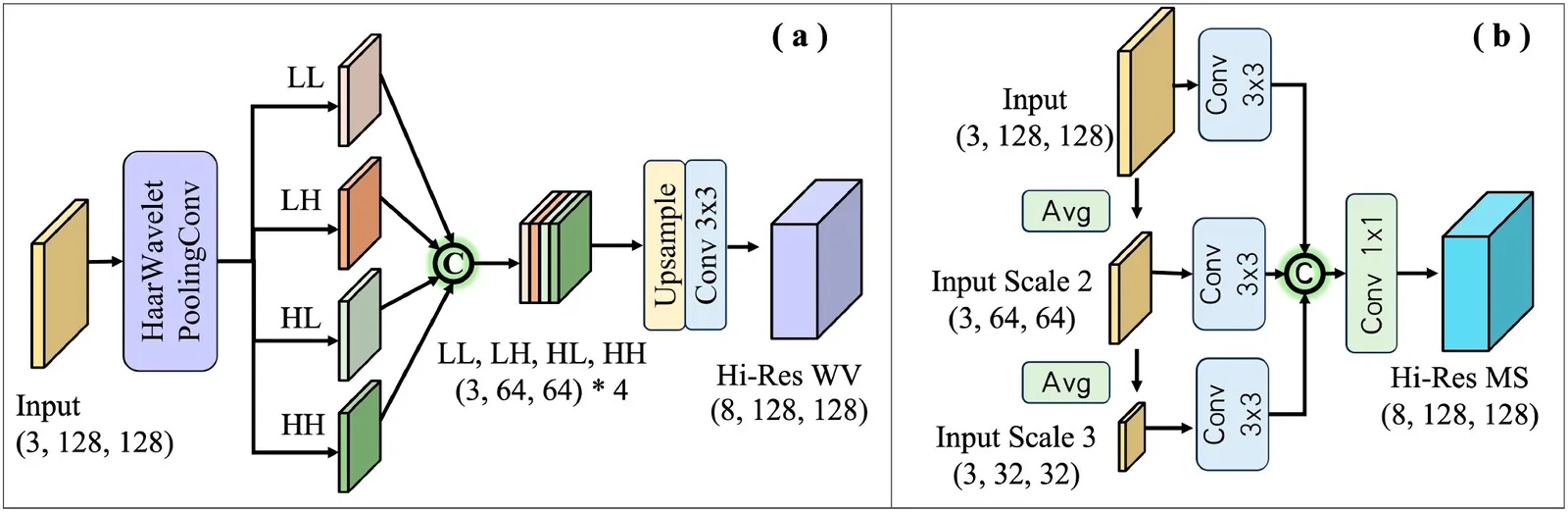
Multi-domain feature encoders. (a) WFE: Haar wavelet decomposition into LL/LH/HL/HH sub-bands, concatenation, and upsampled fusion. (b) MSCE: 3-scale parallel convolution with upsampled fusion.

As shown in Fig. 3b, MSCE extracts multi-granularity spatial context through 3 parallel branches. Each branch applies a $3\, \times \, 3$ convolution at a different spatial scale—original, *1/2*, and *1/4* resolution—to capture features ranging from fine-grained local textures to broader regional morphology. The branch outputs are upsampled to the original resolution, concatenated, and fused via a $1\, \times \, 1$ projection (7,384 trainable parameters), yielding a single full-resolution spatial descriptor $\mathbf {F}_{\mathrm{MSCE}} \in \mathbb {R}^{8 \times 128 \times 128}$. Together with $\mathbf {F}_{\mathrm{WFE}}$, these descriptors compensate for the fine-grained spatial detail that the pretrained backbones lose through downsampling.

The 4 encoders run in parallel, each independently extracting features from the raw input image. The pretrained backbones output structural features $\mathbf {F}_{\mathrm{SAM}}$ and semantic features $\mathbf {F}_{\mathrm{UNI}}$; the multi-domain encoders output $\mathbf {F}_{\mathrm{WFE}}$ and $\mathbf {F}_{\mathrm{MSCE}}$. All 4 feature streams are forwarded to the PFAE-based global fusion neck for alignment and integration.

#### Frequency-aware fusion modules

FPNuNet employs DCT-based frequency-domain processing at 2 distinct architectural stages. At the *encoder adaptation* stage, DCT-based high-pass filtering within the prompt generators extracts boundary and texture cues from the raw input image, compensating for the low-pass effect of ViT patch tokenization. At the *post-encoder fusion* stage, DCT-enabled PFAE modules refine learned feature representations in the frequency domain. DCT is adopted uniformly at both stages because it produces purely real-valued coefficients and concentrates spectral energy of smooth, localized structures—characteristic of IHC nuclear textures—into fewer low-frequency components, providing a compact and efficient spectral basis for both input-level prompt generation and feature-level enhancement. This uniform transform choice also eliminates the complex-number bookkeeping (magnitude/phase storage, complex-to-real conversion) that the fast Fourier transform (FFT) would impose, simplifying the gradient path through both stages. Empirical comparison of transform configurations confirms that the DCT-only design achieves the best overall performance.

The fusion stage comprises a PFAE-based global fusion neck that integrates encoded features from both encoders together with complementary raw-image features, and 2 PFAE-based skip enhancement necks that refine shallow features from the semantic encoder. The following sections detail the PFAE block architecture and the global fusion neck design.

##### PFAE block

Although many segmentation models directly use encoder outputs and skip connection features for decoding, they often fail to preserve high-frequency structural cues essential for accurate nuclear boundary delineation, particularly under stain variability and morphological ambiguity. To address this, we introduce the PFAE block, which combines multi-scale spatial context, DCT-based frequency-domain attention, and positional priors in a residual refinement pipeline.

The overall architecture of the PFAE block is illustrated in Fig. 4a. Let $\mathbf {X} \in \mathbb {R}^{C \times H \times W}$ denote the input feature map. The input undergoes an initial *1 × 1* projection:


\begin{eqnarray*}
\mathbf {F}_0 = \phi _{\mathrm{proj}}(\mathbf {X}),
\end{eqnarray*}

producing a compact embedding $\mathbf {F}_0 \in \mathbb {R}^{C \times H \times W}$ that anchors 3 progressive refinement stages. At each stage *s = 1, 2, 3*, a dilated convolution $\phi _{DC_k}(\cdot )$ with kernel size *3 × 3* and dilation rate $k \in \lbrace 3, 5, 7\rbrace$ increases the receptive field, followed by a lightweight frequency-aware attention (LWFA) module $\psi _{\mathrm{LWFA}}(\cdot )$:


(1) \begin{eqnarray*}
\mathbf {S}_s = \phi _{DC_k}(\mathbf {F}_{s-1}),
\end{eqnarray*}

(2) \begin{eqnarray*}
\mathbf {F}_s &= \mathbf {S}_s + \psi _{\mathrm{LWFA}}(\mathbf {S}_s).
\end{eqnarray*}

**Figure 4:**
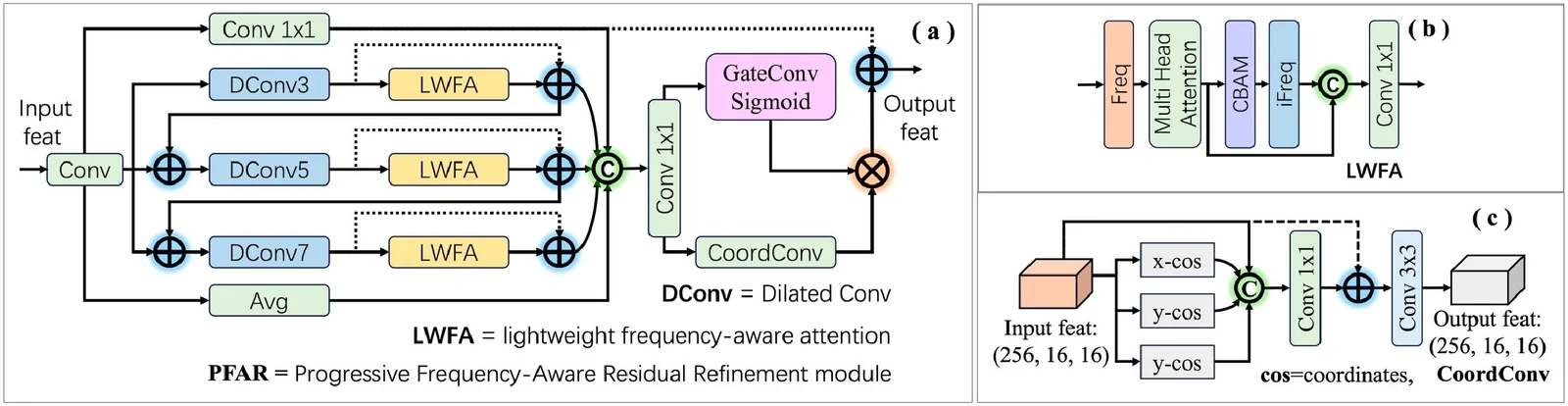
PFAE block architecture. (a) Overview: 3 progressive refinement stages with dilated convolutions, DCT-based LWFA, CBAM, multi-scale aggregation, and coordinate-aware gating. (b) LWFA module. (c) Coordinate-aware gating.

Each $\psi _{\mathrm{LWFA}}(\cdot )$ transforms the spatial feature $\mathbf {S}_s$ into the frequency domain via a real-valued DCT—chosen for the energy-compaction and real-valued coefficient properties discussed above—and applies multi-head attention directly on the resulting spectral embedding to focus on morphology-relevant frequency bands. A convolutional block attention module (CBAM) [34] further refines frequency responses through sequential channel and spatial attention, as shown in Fig. 4b.

To aggregate multi-scale outputs, a global context vector $\mathbf {G}$ is extracted from $\mathbf {F}_0$ via average pooling and fused with the outputs $\lbrace \mathbf {F}_1, \mathbf {F}_2, \mathbf {F}_3\rbrace$ and the initial embedding $\mathbf {F}_0$ through softmax-normalized attention weights $\lbrace \alpha _k\rbrace$:


(3) \begin{eqnarray*}
\mathbf {F} = \mathrm{Concat}\left[\alpha _k \cdot \mathbf {O}_k\right]_{k=0}^{4}, \quad \mathbf {O} = \lbrace \mathbf {F}_0, \mathbf {F}_1, \mathbf {F}_2, \mathbf {F}_3, \mathbf {G}\rbrace.
\end{eqnarray*}

Finally, coordinate-aware gating injects spatial priors, as shown in Fig. 4c:


(4) \begin{eqnarray*}
\mathbf {Y} = \phi (\mathbf {F}) + \delta _{\mathrm{coord}}(\tilde{\mathbf {F}}) \odot \sigma \bigl (\phi _{\mathrm{gate}}(\tilde{\mathbf {F}})\bigr ),
\end{eqnarray*}

where $\tilde{\mathbf {F}}$ is a *1 × 1* convolution of $\mathbf {F}$, $\delta _{\mathrm{coord}}(\cdot )$ injects coordinate priors through trigonometric position encodings, $\sigma (\cdot )$ is the sigmoid activation, and *⊙* denotes element-wise multiplication.

##### PFAE-based global fusion neck

The PFAE-based global fusion neck (Fig. 5) receives the 4 feature streams produced by the multi-source extraction stage: $\mathbf {F}_{\mathrm{SAM}} \in \mathbb {R}^{768 \times 16 \times 16}$, $\mathbf {F}_{\mathrm{UNI}} \in \mathbb {R}^{1024 \times 16 \times 16}$, $\mathbf {F}_{\mathrm{WFE}} \in \mathbb {R}^{8 \times 128 \times 128}$, and $\mathbf {F}_{\mathrm{MSCE}} \in \mathbb {R}^{8 \times 128 \times 128}$.

**Figure 5:**
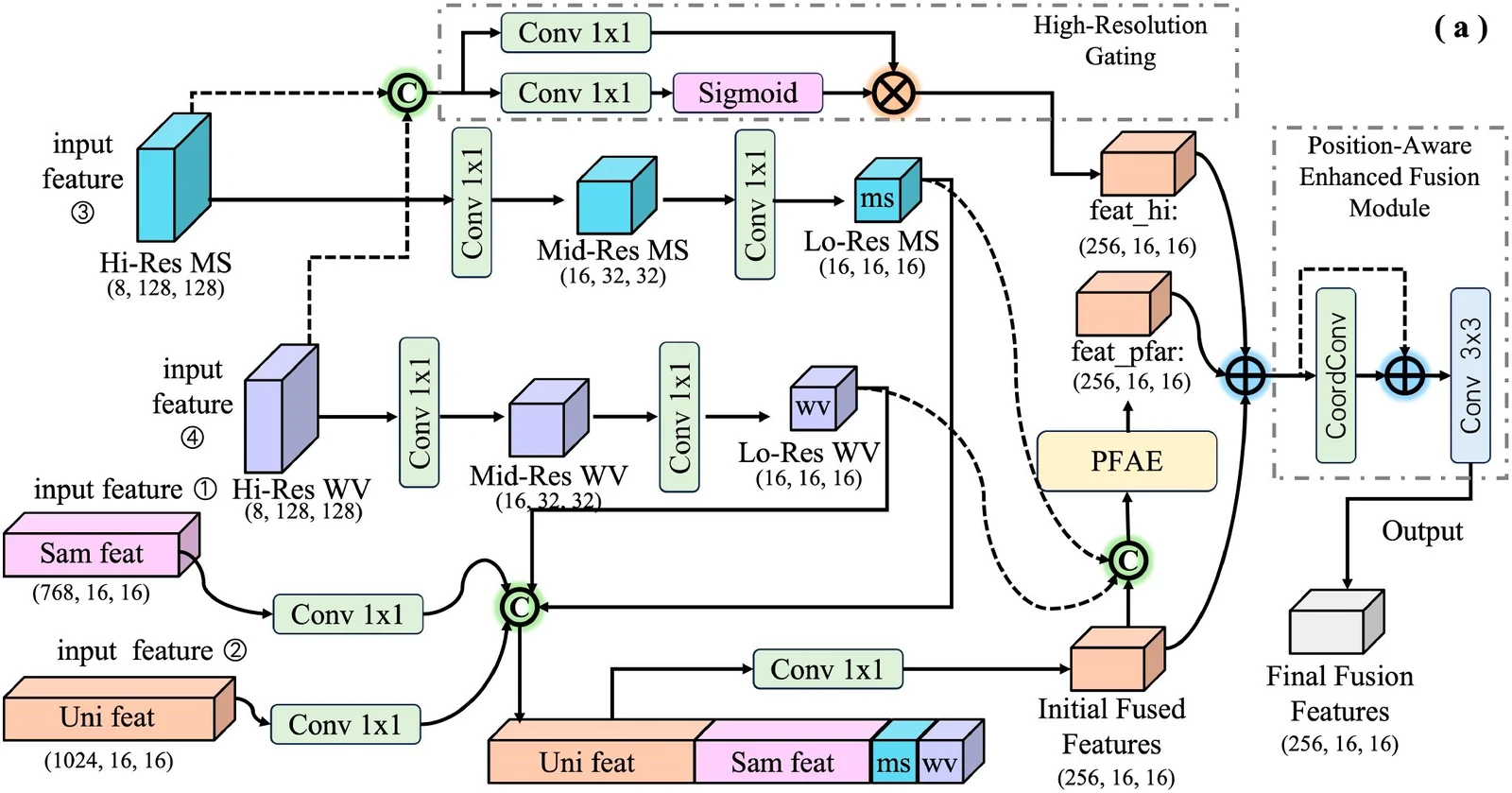
PFAE-based global fusion neck. The neck receives SAM, UNI, WFE, and MSCE features, aligns them at the encoder resolution via channel projection and concatenation, refines the fused representation with a DCT-enabled PFAE block, and injects full-resolution WFE/MSCE detail through a high-resolution sigmoid gate followed by position-aware enhanced fusion.

To align spatial resolutions, the full-resolution WFE and MSCE features are pooled and projected to match the encoder feature resolution. The 4 projected streams are concatenated and fused via a *1 × 1* convolution:


\begin{eqnarray*}
\mathbf {F}_{\mathrm{cat}} = \phi _{\mathrm{fuse}}\bigl (\mathrm{Concat}\bigl [ \phi _{\mathrm{s}}(\mathbf {F}_{\mathrm{SAM}}),\, \phi _{\mathrm{u}}(\mathbf {F}_{\mathrm{UNI}}),\, \tilde{\mathbf {F}}_{\mathrm{WFE}},\, \tilde{\mathbf {F}}_{\mathrm{MSCE}} \bigr ]\bigr ),
\end{eqnarray*}

where $\tilde{\mathbf {F}}_{\mathrm{WFE}}$ and $\tilde{\mathbf {F}}_{\mathrm{MSCE}}$ denote the pooled and projected WFE and MSCE features at the encoder resolution. The fused representation is refined by a DCT-enabled PFAE block with a multi-head attention stage (4 heads), yielding a spectrally enhanced representation $\mathbf {F}_{\mathrm{pfae}}$. To inject high-resolution spatial detail, the full-resolution WFE and MSCE features are concatenated, downsampled, and passed through a gating mechanism:


\begin{eqnarray*}
\mathbf {F}_{\mathrm{out}} = \mathrm{Pos}\bigl (\mathbf {F}_{\mathrm{cat}} + \mathbf {F}_{\mathrm{pfae}} + \sigma (\mathbf {g}) \odot \mathbf {v}\bigr ),
\end{eqnarray*}

where $\mathbf {g}$ and $\mathbf {v}$ are the gate and value projections of the concatenated high-resolution features, $\sigma (\cdot )$ is the sigmoid activation, and $\mathrm{Pos}(\cdot )$ applies position-aware enhancement. The output $\mathbf {F}_{\mathrm{out}} \in \mathbb {R}^{256 \times 16 \times 16}$ is forwarded to all 3 decoder branches.

In addition, 2 DCT-enabled PFAE skip enhancement necks (7,246 parameters total) refine UNI skip features at 2 spatial levels (*32 × 32* and *64 × 64*), strengthening shallow feature propagation to the decoders. The fusion neck accounts for 2,634,507 trainable parameters.

#### Multi-task decoders with hierarchical guidance

Accurate nuclear quantification necessitates the concurrent resolution of 3 interrelated tasks: binary segmentation to identify nuclear regions, instance separation to delineate individual cells, and phenotype classification to assign biologically meaningful subtypes. FPNuNet adopts a sequential, causally guided multi-task decoder inspired by HoVer-Net [7], consisting of 3 task-specific branches—binary, HV, and type decoders. The binary decoder executes first and produces multi-scale intermediate feature maps; these features (not final logits) are then propagated to both the HV and type decoders through sigmoid-gated fusion modules, reinforcing nuclear localization and cross-task consistency at the feature level. All branches receive multi-scale, DCT-enhanced features from the PFAE-based fusion neck and skip pathways, ensuring that both structural fidelity and semantic richness are preserved throughout decoding.

##### Binary segmentation decoder

The binary decoder (578,628 parameters; Fig. 6b) serves as the structural foundation for both instance separation and phenotype classification. It adopts a 3-stage hierarchical design where, at each stage, fused input features are upsampled via pixel-shuffle blocks (*1 × 1* convolution followed by PixelShuffle(2), batch normalization, and ReLU) and concatenated with DCT-enhanced skip features from the UNI encoder. The concatenated feature map is processed by a convolutional refinement block to produce resolution-aligned feature maps. The decoder outputs binary nuclei logits and boundary logits. Critically, it also outputs multi-scale intermediate feature maps that provide prior guidance for the downstream HV and type decoders.

**Figure 6:**
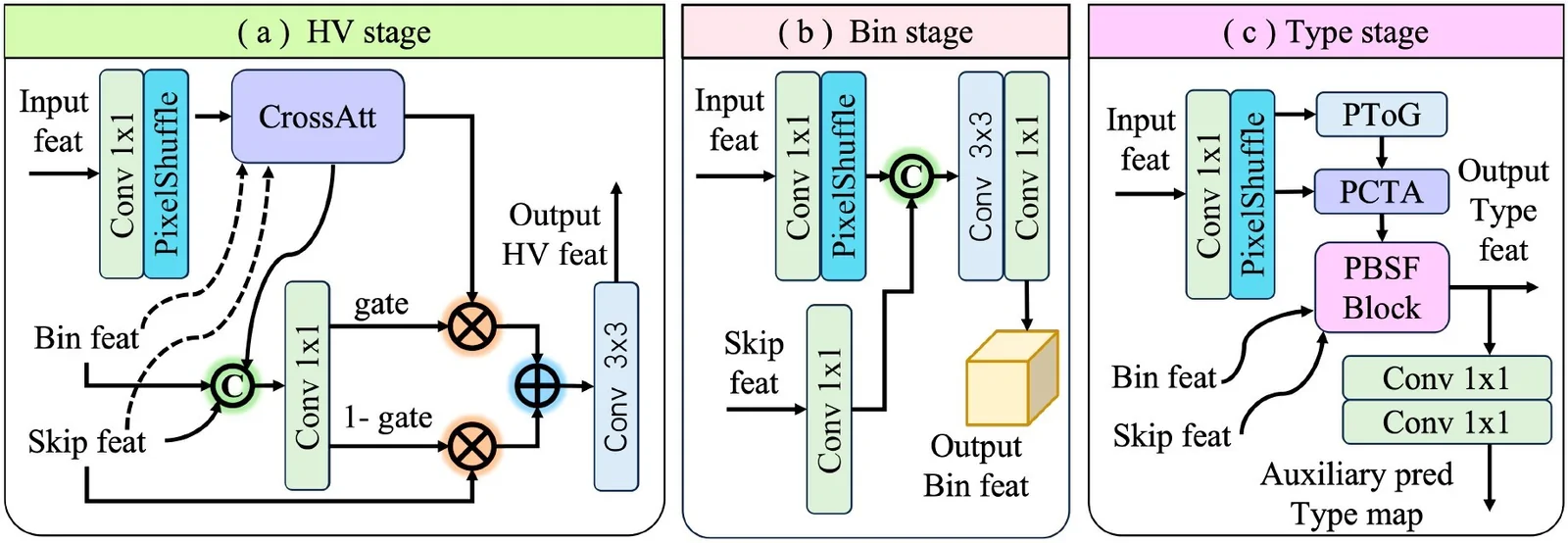
Collaborative multi-task decoder with hierarchical binary guidance. (a) HV vector regression decoder (binary-guided, bilinear upsampling, dense refinement). (b) Binary segmentation decoder (produces logits and intermediate feature guidance maps). (c) Nuclear type classification decoder (prompt-to-feature cross-attention, binary-guided skip fusion).

The binary branch is supervised by a composite loss:


(5) \begin{eqnarray*}
\mathcal {L}_{\mathrm{bin}} = \mathcal {L}_{\mathrm{BCE}} + \mathcal {L}_{\mathrm{Dice}} + \mathcal {L}_{\mathrm{Focal}} + 0.5 \cdot \mathcal {L}_{\mathrm{boundary}},
\end{eqnarray*}

where $\mathcal {L}_{\mathrm{boundary}}$ supervises the predicted boundary map to sharpen nuclear contour delineation, addressing class imbalance and providing reliable structural priors for the other branches.

##### HV vector regression decoder

The HV decoder (763,718 parameters; Fig. 6a) estimates pixel-wise horizontal and vertical offsets $\hat{\mathbf {V}} \in \mathbb {R}^{2 \times H \times W}$ pointing from each pixel to its corresponding nuclear centroid for instance-level separation. The HV decoder employs bilinear upsampling blocks (replacing pixel-shuffle), dense feature refinement blocks for local detail recovery, and a unified binary-guided skip fusion module at each stage. Each fusion module applies cross-attention with decoder features as queries and UNI skip features as keys and values, followed by a sigmoid-gated modulation using binary decoder intermediate features. This mechanism allows binary segmentation priors to adaptively control how skip features enter the HV prediction path.

Supervision adopts foreground-masked regression:


(6) \begin{eqnarray*}
\mathcal {L}_{\mathrm{hv}} = \mathcal {L}_{\mathrm{MSE}}^{\mathrm{fg}} + \mathcal {L}_{\mathrm{MSGE}}^{\mathrm{full-grad}},
\end{eqnarray*}

where $\mathcal {L}_{\mathrm{MSE}}^{\mathrm{fg}}$ applies mean squared error only within the foreground mask, and $\mathcal {L}_{\mathrm{MSGE}}^{\mathrm{full-grad}}$ denotes the full-gradient mean squared gradient error computed from Sobel derivatives, promoting smooth and spatially coherent HV maps. Both terms carry equal weight (1.0).

##### Nuclear type classification decoder

The type decoder (1,104,219 parameters; Fig. 6c) classifies each pixel into one of *K* nuclear subtypes through a dedicated prompt-to-feature cross-attention mechanism.

At each of the 3 decoding stages, the decoder receives the same tri-source input as the HV decoder: upsampled features from the previous stage, DCT-enhanced UNI skip features, and binary decoder intermediate feature maps. To introduce global semantic priors, a prompt token generator produces learnable type-specific tokens. In contrast to earlier decoder versions where spatial features queried prompt tokens, the type decoder reverses the attention direction: *learned prompt tokens serve as Query, while pooled decoder features serve as Key and Value* in multi-head cross-attention. This design enables each type prompt to actively retrieve image-specific evidence from the entire spatial feature map, producing a prompt-conditioned global vector that is projected back to the feature dimension and added residually to the decoder features. Subsequently, binary-guided skip fusion (identical to the HV decoder) modulates the contribution of structural and semantic information through sigmoid-gated feature integration.

The type branch is supervised by a multi-term classification loss with equal weighting (1.0) for all 4 terms:


(7) \begin{eqnarray*}
\mathcal {L}_{\mathrm{type}} = \mathcal {L}_{\mathrm{CE}}^{w} + \mathcal {L}_{\mathrm{Dice}} + \mathcal {L}_{\mathrm{Focal}} + \mathcal {L}_{\mathrm{IoU}},
\end{eqnarray*}

where $\mathcal {L}_{\mathrm{CE}}^{w}$ is class-weighted cross-entropy in which background is down-weighted (weight 0.25) relative to all foreground classes (weight 1.0), and $\mathcal {L}_{\mathrm{IoU}}$ is a soft IoU loss. The focal term uses a focusing parameter of $\gamma {=}2.0$. As described in the “Data description,” section rare and morphologically ambiguous categories are merged before training, yielding 4 foreground classes (pTu, pIm, nTu, nOth) plus background.

##### Overall loss

The overall training objective combines all 3 branch losses with equal weight:


(8) \begin{eqnarray*}
\mathcal {L}_{\mathrm{total}} = \mathcal {L}_{\mathrm{bin}} + \mathcal {L}_{\mathrm{hv}} + \mathcal {L}_{\mathrm{type}}.
\end{eqnarray*}

At inference, individual nuclear instances are extracted from the predicted binary mask and HV map using the same Sobel-gradient-based watershed post-processing as HoVer-Net [7], and each instance is assigned the majority predicted type from the type map.

## Results

### Experimental setup

We evaluated FPNuNet on the curated CD47-IHCNuSC dataset, processed into overlapping $128\, \times \, 128$ patches (stride 64) with augmentation via flips, rotations, color jittering, and stain perturbation. FPNuNet was trained with the AdamW optimizer (batch size 8, weight decay $1 \times 10^{-2}$) using bf16 mixed precision, adopting a dual-rate learning schedule: $1 \times 10^{-3}$ for prompt generator parameters and $5 \times 10^{-4}$ for all other trainable components. Both learning-rate groups followed a step-level schedule with linear warmup over 1,000 steps and multiplicative decay by 0.5 at steps 20,000 and 27,000, for a total of 30,000 optimizer steps (*∼*12 epochs). The pretrained SAM (natural images) and UNI (pathology images) encoders provided initial representations, with prompt generators fine-tuned jointly. All experiments were conducted using PyTorch 2.0 and MONAI on a workstation equipped with 4 NVIDIA RTX 4090 GPUs.

#### Model complexity

FPNuNet contains 394.8 M total parameters, of which only 5.4 M (1.37%) are trainable. The majority of parameters reside in the frozen UNI backbone (303.4 M) and the frozen SAM backbone (86.1 M). Among trainable components, the fusion neck is the largest (2.6 M), followed by the type decoder (1.1 M), HV decoder (0.8 M), binary decoder (0.6 M), and SAM/UNI prompt generators (0.2 M combined). Table 2 compares model complexity for all baselines amenable to THOP-based profiling. Despite having the largest total parameter count, FPNuNet has the lowest proportion of trainable parameters among the compared methods (5.4 M, 1.37%)—over $10{\times }$ fewer trainable parameters than HoVer-Net (54.7 M) and over $56{\times }$ fewer than Cellpose-SAM (304.6 M). Its FLOPs (111.2 G) are likewise lower than those of HoVer-Net (394.1 G) and Cellpose-SAM (621.1 G). On a single NVIDIA RTX 4090 GPU with a $128{\times }128$ input, the sequential 4-encoder forward pass averages 106.7 ms latency and 1.62 GB peak memory, higher than single-backbone baselines (e.g., HoVer-Net: 13.6 ms) owing to the serial execution of 2 frozen ViT encoders.

**Table 2:** Model complexity comparison.

Model	Input	Total	Trainable	FLOPs
		Params	Params	
HoverFast	$256^2$	0.95 M	0.95 M (100%)	33.2 G
HoVer-Net	$270^2$	54.7 M	54.7 M (100%)	394.1 G
Cellpose-SAM	$256^2$	304.6 M	304.6 M (100%)	621.1 G
FPNuNet	$128^2$	394.8 M	5.4 M (1.37%)	111.2 G

FLOPs are reported as *2×*multiply–accumulate operations on a single NVIDIA RTX 4090 GPU; profiled with THOP. StarDist is omitted owing to its TensorFlow backend (incompatible with THOP); SRSA-Net is omitted because the publicly released code does not expose a standalone model class suitable for THOP profiling.

FPNuNet is compared against 5 baseline methods: **HoVer-Net** [7], **SRSA-Net** [13], **StarDist** [35], **HoverFast** [32], and **Cellpose-SAM** [36]. To evaluate the contribution of key components, several FPNuNet variants are included in the ablation study: **FPNuNet-F** (dual encoders SAM+UNI with FFT-based frequency-aware enhancement, concatenation fusion neck, standard binary/HV/type decoders, no prompt adapter on UNI), **FPNuNet-A** (the baseline augmented with symmetric prompt generators on both encoders for cross-domain feature alignment), and **FPNuNet-AP** (the adapter variant further augmented with a prompt-guided type decoder that injects learnable subtype tokens via cross-attention into the type branch). The full **FPNuNet** model additionally replaces the FFT-based prompt generators with DCT-based variants, upgrades the fusion and skip necks to DCT-enabled PFAE, upgrades the HV and type decoder architectures, and adds skip-connection enhancement necks.

For a controlled comparison, the FPNuNet series, HoVer-Net, SRSA-Net, HoverFast, and Cellpose-SAM were trained on the same CD47-IHCNuSC training split using consistent training and evaluation protocols whenever applicable. Since StarDist, HoverFast, and Cellpose-SAM are instance-segmentation–oriented methods, they were included in the instance-level segmentation comparisons but not in subtype classification evaluation. StarDist was evaluated with its publicly released pretrained model as an off-the-shelf instance segmentation baseline.

### Evaluation and metrics

The performance of nuclei analysis models is assessed along 2 complementary axes: (1) nuclear instance segmentation, which evaluates the recovery of object identities and boundaries; and (2) nuclear classification, which covers both dense, pixel-wise semantic segmentation (treated as per-pixel classification) and instance-level subtype recognition.

#### Nuclear instance segmentation evaluation

Nuclear instance segmentation is quantified using complementary criteria that assess how well a method detects, separates, and delineates individual instances. Given a set of ground-truth (GT) nuclear masks $\mathcal {G}$ and a set of predicted nuclear masks $\mathcal {S}$ for an image, region-overlap metrics such as the Intersection-over-Union (IoU) and Dice coefficient (**Dice**) [37] measure pixel-wise agreement between the corresponding regions:


(9) \begin{eqnarray*}
\mathrm{IoU} = \frac{|G \cap S|}{|G \cup S|},
\end{eqnarray*}

(10) \begin{eqnarray*}
\mathrm{Dice}= \frac{2\, |G \cap S|}{|G| + |S|}.
\end{eqnarray*}

The pairwise aggregate variant (**Dice2**) [38] computes Dice over all matched GT–prediction pairs and averages them, providing a more instance-sensitive estimate of segmentation accuracy.

To further incorporate unmatched regions, the aggregated Jaccard index (AJI) [39] aggregates intersections and unions over all matched pairs, penalizing unpaired GT or prediction regions:


(11) \begin{eqnarray*}
\mathrm{AJI} = \frac{ \sum |G \cap S| }{ \sum |G \cup S| + \sum |G_{\mathrm{unmatched}}| + \sum |S_{\mathrm{unmatched}}| }.
\end{eqnarray*}

Its assignment-robust variant AJI+ replaces greedy matching with optimal bipartite (Hungarian) assignment to better handle clumped or overlapping nuclei [7].

Following the HoVer-Net [7] and SRSA-Net evaluation protocol [13], we report panoptic quality (PQ) [40] together with its decomposition into detection quality (DQ) and segmentation quality (SQ). Binary PQ (bPQ) is computed across all nuclei regardless of type, while the type-aware counterpart evaluates per-class panoptic quality. Let $\mathrm{TP}$ (true positives), $\mathrm{FP}$ (false positives), and $\mathrm{FN}$ (false negatives) represent correctly matched nuclei, unmatched predictions, and missed GT nuclei, respectively. Then DQ and SQ are defined as


(12) \begin{eqnarray*}
\mathrm{DQ} = \frac{\mathrm{TP}}{\mathrm{TP} + 0.5\, \mathrm{FP} + 0.5\, \mathrm{FN}},
\end{eqnarray*}

(13) \begin{eqnarray*}
\mathrm{SQ} = \frac{1}{\mathrm{TP}} \sum \mathrm{IoU}(G, S),
\end{eqnarray*}

and the overall PQ is obtained as


(14) \begin{eqnarray*}
\mathrm{PQ} = \mathrm{DQ} \times \mathrm{SQ}.
\end{eqnarray*}

In addition, we evaluate model performance using precision ($\mathbf {P}$), recall ($\mathbf {R}$), and *F*_1_-score ($\mathbf {F}_1$), which are mathematically defined as


(15) \begin{eqnarray*}
\mathbf {P} = \frac{\mathrm{TP}}{\mathrm{TP} + \mathrm{FP}}, \quad \mathbf {R} = \frac{\mathrm{TP}}{\mathrm{TP} + \mathrm{FN}}, \quad \mathbf {F}_1 = \frac{2\, \mathbf {P}\, \mathbf {R}}{\mathbf {P} + \mathbf {R}}.
\end{eqnarray*}

To distinguish these from pixel-level measures, we denote the object-level precision ($\mathbf {P}_{\mathrm{obj}}$), recall ($\mathbf {R}_{\mathrm{obj}}$), and *F*_1_-score ($\mathbf {F}_{1, \mathrm{obj}}$), computed from nucleus-level TP, FP, and FN counts. Together, these metrics cover pixel-wise overlap, instance separation, boundary accuracy, and assignment robustness.

#### Nuclear classification evaluation

Nuclear type classification is evaluated using instance-level metrics that assess the correctness of subtype labels assigned to individual nuclei while accounting for detection performance. Given the GT set with type annotations $\mathcal {G}=\lbrace (g_i, y_i)\rbrace _{i=1}^{N}$ and the predicted set with assigned types $\mathcal {S}=\lbrace (s_j, \hat{y}_j)\rbrace _{j=1}^{M}$, a one-to-one correspondence $\mathcal {M} \subseteq \mathcal {G} \times \mathcal {S}$ is established by matching predicted and GT nuclei. Each matched prediction is then assigned the class with the highest predicted score. Background (class 0) is excluded.

For each matched pair $(g_i, s_j)$, a TP is counted if $\hat{y}_j = y_i$. A label mismatch contributes a FP for the predicted class $\hat{y}_j$ and a FN for the GT class $y_i$. Unmatched predictions and unmatched GT instances are counted as FP and FN, respectively. This matching procedure yields the per-class counts $\lbrace \mathrm{ TP}^{(k)}, \mathrm{ FP}^{(k)}, \mathrm{ FN}^{(k)}\rbrace _{k=1}^{K}$, which form the basis for the macro-, micro-, and support-weighted F$_1$ scores, as well as accuracy variants outlined below.

For each class $k \in \lbrace \mathrm{pTu}, \mathrm{pIm}, \mathrm{nTu}, \mathrm{nOth}\rbrace$ (excluding background), the instance-level precision (${P}^{(k)}_{\mathrm{inst}}$), recall (${R}^{(k)}_{\mathrm{inst}}$), and *F*_1_-score (${F}^{(k)}_{1,\mathrm{inst}}$) are calculated as follows:


(16) \begin{eqnarray*}
{P}^{(k)}_{\mathrm{inst}} = \frac{\mathrm{ TP}^{(k)}}{\mathrm{ TP}^{(k)} + \mathrm{ FP}^{(k)}}, \quad {R}^{(k)}_{\mathrm{inst}} = \frac{\mathrm{ TP}^{(k)}}{\mathrm{ TP}^{(k)} + \mathrm{ FN}^{(k)}},
\end{eqnarray*}

(17) \begin{eqnarray*}
{F}^{(k)}_{1,\mathrm{inst}} = \frac{2\, {P}^{(k)}_{\mathrm{inst}}\, {R}^{(k)}_{\mathrm{inst}}}{{P}^{(k)}_{\mathrm{inst}} + {R}^{(k)}_{\mathrm{inst}}}.
\end{eqnarray*}

The macro-averaged scores $\mathbf {P}_{\text{cls, macro}}$ and $\mathbf {F}_{1, \mathrm{macro}}$ are obtained by averaging the per-class instance-level precision and *F*$_1$ with equal weights. The support-weighted metrics $\mathbf {P}_{\mathrm{w}}$ and $\mathbf {F}_{1,\mathrm{w}}$ weight per-class scores by class support, with $w_k=n^{(k)}/N$ where $n^{(k)}$ is the number of GT nuclei in class *k* and $N=\sum _k n^{(k)}$. The micro-averaged *F*$_1$ score $\mathbf {F}_{1, \mathrm{micro}}$ is computed from the TP, FP, and FN counts aggregated across all classes.

For further evaluation, the overall metrics include the mean panoptic quality (mPQ) over non-background classes, computed as $\mathrm{mPQ} = \frac{1}{K}\sum _{k=1}^{K} \mathrm{PQ}^{(k)}$, where $\mathrm{PQ}^{(k)} = \mathrm{DQ}^{(k)} \times \mathrm{SQ}^{(k)}$ as defined in the instance segmentation section. A match is counted as $\mathrm{ TP}^{(k)}$ only when the predicted type equals the GT type *k* (type-consistent PQ).

### Nuclear instance segmentation performance

FPNuNet achieves the strongest overall instance-segmentation performance on CD47-IHCNuSC across region-overlap, boundary, and detection metrics (Table 3; Figs. 7 and 8). The box plots in Fig. 8 reveal higher medians and tighter interquartile ranges for AJI, AJI+, and bPQ, indicating stable gains across individual test images rather than improvements confined to a small subset. Qualitatively, FPNuNet produces cleaner splits in crowded clusters, smoother yet well-aligned contours along membrane staining, and fewer missed small nuclei than competing methods (Fig. 7).

**Figure 7:**
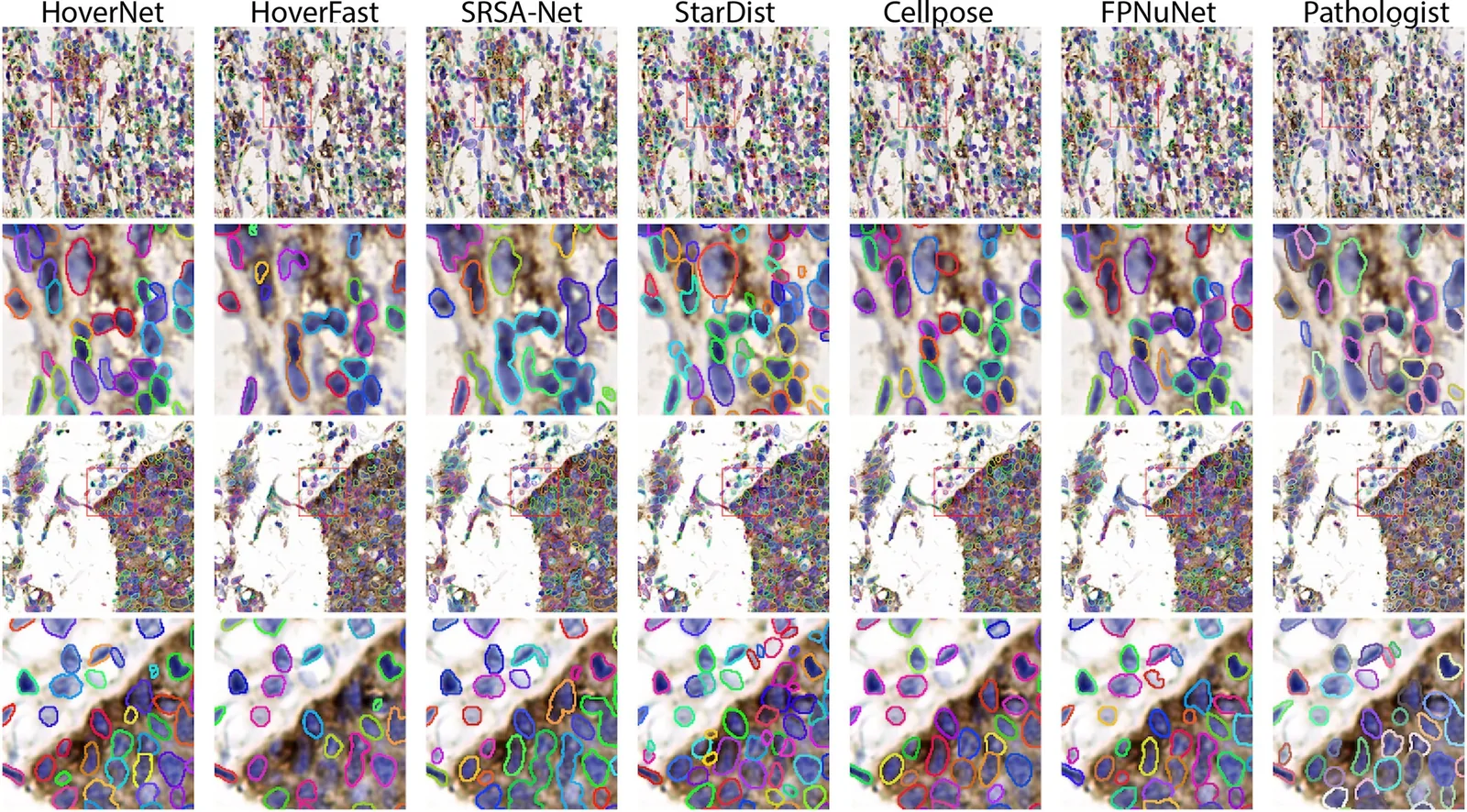
Qualitative comparisons of instance segmentation models on representative CD47-IHCNuSC test samples. Colors are used solely to distinguish individual nuclei. Cellpose denotes Cellpose-SAM.

**Figure 8:**
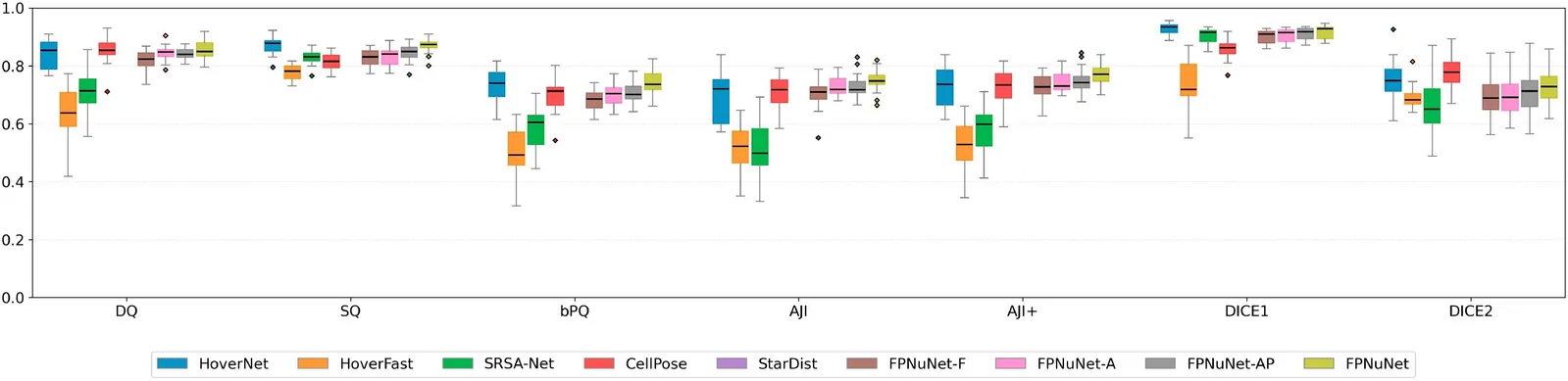
Box plots highlighting the performance of competing instance segmentation methods on the CD47-IHCNuSC dataset. Each box summarizes the distribution of evaluation metrics (*DQ, SQ, bPQ, AJI, AJI+, Dice*, and *Dice2*) across all test images.

**Table 3:** Quantitative evaluation of instance segmentation on the CD47-IHCNuSC test set.

Model	Dice	Dice2	AJI	AJI+	bPQ	DQ	SQ	${F}_{1,\mathrm{ inst}}$	${P}_{\mathrm{ inst}}$	${R}_{\mathrm{ inst}}$
HoVer-Net	**0.9264**	0.7541	0.6962	0.7307	0.7303	0.8387	**0.8700**	0.8438	0.8647	0.8264
SRSA-Net	0.9051	0.6640	0.5101	0.5822	0.5872	0.7076	0.8292	0.7189	0.7964	0.6588
HoverFast	0.7403	0.6944	0.5144	0.5256	0.5057	0.6483	0.7785	0.6524	0.7395	0.5903
StarDist	0.7019	0.5751	0.4503	0.4697	0.3529	0.4737	0.7402	0.4753	0.3784	0.7114
Cellpose-SAM	0.8585	**0.7766**	0.7101	0.7263	0.6970	0.8542	0.8147	0.8594	**0.9130**	0.8147
FPNuNet-F	0.9017	0.6917	0.7048	0.7256	0.6814	0.8221	0.8286	0.8266	0.7882	0.8723
FPNuNet-A	0.9055	0.6992	0.7298	0.7443	0.7018	0.8443	0.8310	0.8490	0.8109	**0.8921**
FPNuNet-AP	0.9100	0.7118	0.7303	0.7502	0.7114	0.8431	0.8436	0.8477	0.8122	0.8880
FPNuNet	0.9215	0.7296	**0.7472**	**0.7717**	**0.7458**	**0.8589**	0.8677	**0.8637**	0.8462	0.8834

Best result per metric in **bold**.

To quantify the statistical reliability of observed differences given the limited test set ($N\,{=}\,16$), we conducted paired per-image bootstrap 95% confidence interval estimation (10,000 resamples) and two-sided Wilcoxon signed-rank tests with Holm–Bonferroni correction for multiple comparisons. On AJI, FPNuNet yields higher mean values than every compared method and ablation variant, with statistically significant differences after correction only for SRSA-Net, HoverFast, StarDist, and FPNuNet-F. The mean differences relative to StarDist and HoverFast are *+0.2969*, (95% CI $[0.2544,\,\, 0.3434]$) and *+0.2328* (95% CI $[0.2111,\,\, 0.2532]$), respectively. On bPQ and SQ, FPNuNet yields higher values than all methods except HoVer-Net, for which the observed differences are small (bPQ: *+0.0155*, *P = 0.6343*; SQ: *-0.0024*, *P = 0.9908*). Conversely, on Dice2, FPNuNet scores lower than HoVer-Net (*-0.0245*, 95% CI $[-0.0368,\,\, -0.0127]$) and Cellpose-SAM (*-0.0470*, 95% CI $[-0.0651,\,\, -0.0278]$), suggesting that this pairwise overlap measure favors methods with dedicated semantic segmentation heads. On DQ, FPNuNet yields higher values than SRSA-Net (*+0.1513*), HoverFast (*+0.2106*), StarDist (*+0.3852*), and FPNuNet-F (*+0.0368*; corrected *P < 0.01*), whereas the smaller margins over HoVer-Net (*+0.0202*, *P = 0.5607*), Cellpose-SAM (*+0.0048*, *P = 0.9908*), FPNuNet-A, and FPNuNet-AP remain above the corrected threshold. On Dice, FPNuNet scores 0.008 lower than HoVer-Net (*-0.0049*, 95% CI $[-0.0075,\,\, -0.0025]$) and higher than every other compared method and ablation variant, including Cellpose-SAM (*+0.0630*, 95% CI $[0.0479,\,\, 0.0822]$, *P = 0.0017*). Within the ablation family, all 3 partial configurations (FPNuNet-F, -A, -AP) have lower mean AJI, bPQ, and SQ than the full model. After correction, the bPQ and SQ differences remain significant for all 3 configurations, whereas the AJI difference remains significant only for FPNuNet-F (corrected *P < 0.05*).

Ablation across the FPNuNet family reveals progressive and complementary contributions from each module. Introducing adapters (FPNuNet-F *→* FPNuNet-A) yields the largest detection-quality gain in the ablation sequence (*+2.22* percentage points [pp] DQ; Table 4), with concurrent improvements across all overlap metrics, suggesting that adapters primarily improve instance recovery and contact handling under weak IHC signal. Adding the prompt-guided decoder (FPNuNet-AP) further strengthens boundary quality (*+0.96* pp bPQ) and shape fidelity (*+1.26* pp SQ), while detection quality is essentially maintained. The complete FPNuNet configuration produces the largest single-step gain, with broad-based improvements led by bPQ (*+3.44* pp) and SQ (*+2.41* pp), accompanied by higher $\mathbf {P}_{\mathrm{inst}}$ at negligible cost to $\mathbf {R}_{\mathrm{inst}}$ (Table 4). These trends are also reflected in the distributions shown in Fig. 8, as well as the qualitative overlays in Fig. 7, which show fewer missed detections, reduced residual merges in crowded regions, and more stable boundary delineation across the progressively enhanced configurations.

**Table 4:** Ablation study on CD47-IHCNuSC.

Config	Added	AJI	bPQ	DQ	SQ
FPNuNet-F	Baseline	0.7048	0.6814	0.8221	0.8286
FPNuNet-A	+Adapter	0.7298	0.7018	0.8443	0.8310
	*Δ*	*+2.50*	*+2.04*	*+2.22*	*+0.24*
FPNuNet-AP	+Type Dec.	0.7303	0.7114	0.8431	0.8436
	*Δ*	*+0.05*	*+0.96*	*–0.12*	*+1.26*
FPNuNet	+DCT-PFAE	**0.7472**	**0.7458**	**0.8589**	**0.8677**
	*Δ*	*+1.69*	*+3.44*	*+1.58*	*+2.41*

Each row adds one module to the preceding configuration. Metrics use the same pipeline as Table 3. *Δ* denotes thepp changefrom the previous row. Bold values indicates the best-performing values, and italics values indicate the *Δ* values.

The transition from FPNuNet-AP to the full FPNuNet encompasses 4 coupled changes: replacing FFT with DCT in both the prompt generators and the frequency-aware fusion modules, upgrading the PFAE block architecture, and introducing the skip-connection enhancement neck. Although the ablation design does not isolate each factor individually, the combined effect constitutes the largest single-step improvement in the sequence (Table 4), consistent with DCT’s energy-compaction advantage on localized IHC nuclear textures. A separate internal ablation suggests that the FFT-to-DCT substitution alone yields consistent gains; however, because this intermediate configuration was not included in the main evaluation protocol, we report the combined effect and note the design coupling as a limitation.

Among baselines trained on CD47-IHCNuSC under identical protocols, FPNuNet achieves the strongest instance-segmentation performance (Table 3). Relative to HoVer-Net, FPNuNet improves AJI by 5.1 pp, reflecting primarily recall-driven gains ($\mathbf {R}_{\mathrm{inst}}$: 0.883 vs. 0.826) at a modest precision trade-off ($\mathbf {P}_{\mathrm{inst}}$: 0.846 vs. 0.865), while maintaining comparable shape fidelity (SQ). These gains indicate more complete recovery of occluded and touching nuclei without sacrificing contour quality, as confirmed by tighter interquartile ranges in Fig. 8 and cleaner cluster splits in Fig. 7. Against SRSA-Net, margins are substantially larger (e.g., AJI *+23.7* pp), consistent with SRSA-Net’s limited recall under dense packing; Fig. 7 further shows residual merges and contour bleeding for SRSA-Net, whereas FPNuNet maintains well-delineated boundaries.

Among the instance-only baselines retrained on CD47-IHCNuSC, FPNuNet recorded higher AJI and bPQ than Cellpose-SAM and HoverFast (Table 3); StarDist was evaluated separately using its publicly released pretrained weights. Taken together, these results summarize the detection, separation, and contour performance of FPNuNet on CD47-IHCNuSC (Table 3; Figs. 8 and 7).

### Nuclear type classification performance

FPNuNet achieves the strongest aggregate subtype classification performance on CD47-IHCNuSC (Tables 5 and 6; Fig. 9), leading all compared methods on mPQ, $\mathbf {P}_{\mathrm{macro}}$, $\mathbf {F}_{1,\mathrm{macro}}$, $\mathbf {F}_{1,\mathrm{micro}}$, and $\mathbf {F}_{1,w}$. HoVer-Net retains the highest prevalence-weighted precision $\mathbf {P}_{w}$ (0.8761; FPNuNet: 0.8586), yet FPNuNet’s higher macro-averaged precision and F$_1$ point to better cross-class balance rather than reliance on majority categories. Qualitative overlays in Fig. 9 corroborate these trends, with fewer subtype flips at tumor–stroma interfaces and more coherent immune predictions under FPNuNet.

**Figure 9:**
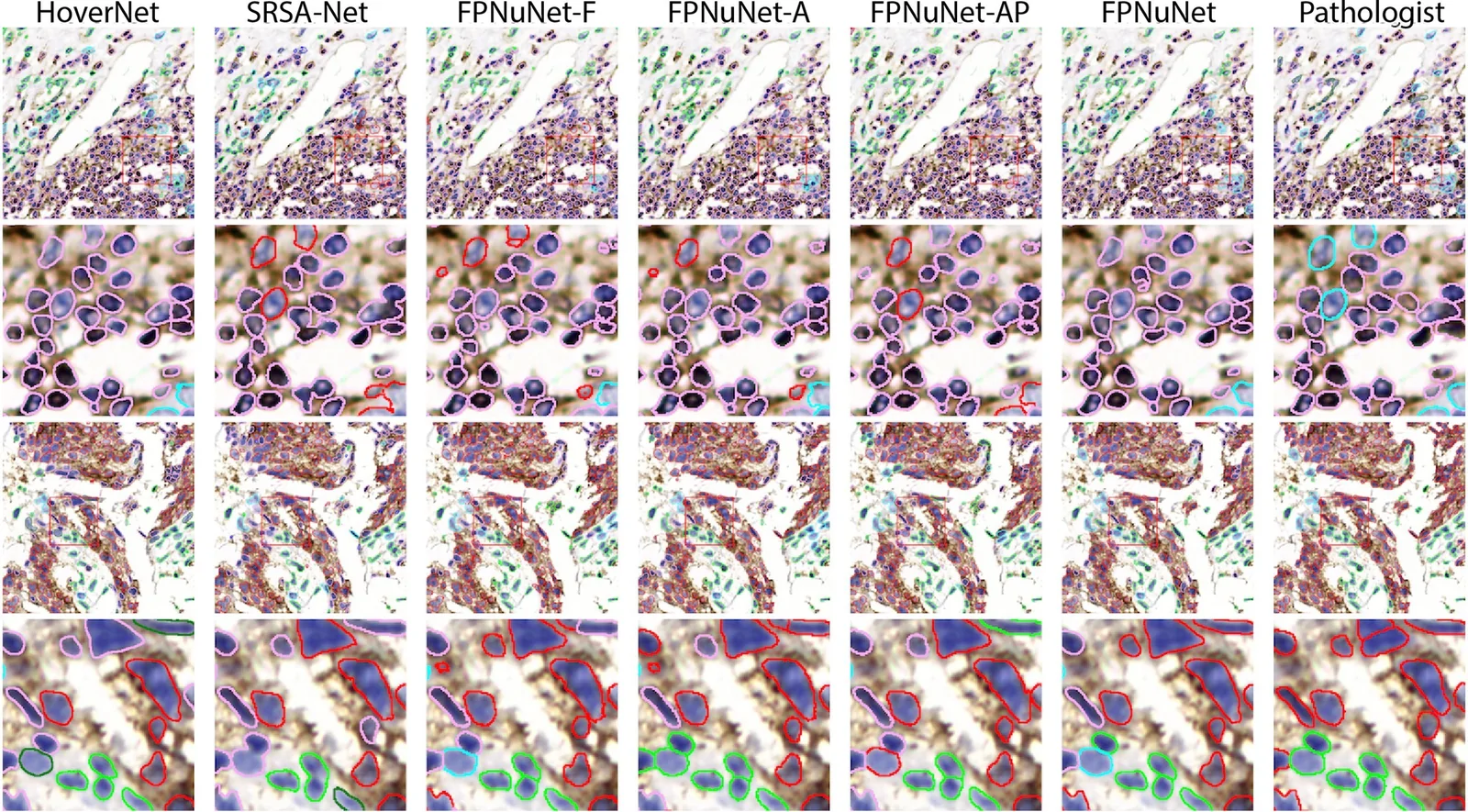
Qualitative comparisons of NuSC-capable models on representative CD47-IHCNuSC test samples. All models output instance masks; for models that support classification, subtype predictions are color-coded according to the legend below. Cellpose denotes Cellpose-SAM.

**Table 5:** Quantitative evaluation of classification on the CD47-IHCNuSC test set (overall metrics).

Model	${\mathrm{ mPQ}}$	${P}_{\mathrm{ macro}}$	${P}_{w}$	${F}_{1,\mathrm{ macro}}$	${F}_{1,\mathrm{ micro}}$	${F}_{1,w}$
HoVer-Net	0.4801	0.6966	**0.8761**	0.6349	0.7984	0.8172
SRSA-Net	0.3661	0.7029	0.8648	0.5779	0.7410	0.7668
FPNuNet-F	0.3664	0.5985	0.7929	0.5028	0.7767	0.7634
FPNuNet-A	0.3520	0.5534	0.7788	0.4653	0.7647	0.7513
FPNuNet-AP	0.4365	0.6888	0.8458	0.5879	0.7906	0.8000
FPNuNet	**0.5074**	**0.7097**	0.8586	**0.6481**	**0.8239**	**0.8317**

Best in **bold**.

**Table 6:** Per-class classification on the CD47-IHCNuSC test set.

	pTu				pIm				nTu				nOth			
Model	PQ	*P*	*R*	*F* $_1$	PQ	*P*	*R*	*F* $_1$	PQ	*P*	*R*	*F* $_1$	PQ	*P*	*R*	*F* $_1$
HoVer-Net	0.6649	**0.9496**	0.8577	0.9013	**0.5564**	0.7324	**0.8534**	0.7883	0.3046	0.5575	**0.5808**	0.5689	0.3945	**0.6547**	0.6282	0.6412
SRSA-Net	0.5207	0.8984	0.8395	0.8679	0.4124	0.6640	0.8130	0.7310	0.2385	0.5766	0.4812	0.5246	0.2927	0.6314	0.5533	0.5897
FPNuNet-F	0.6157	0.8724	**0.9353**	0.9028	0.2760	0.7344	0.7526	0.7434	0.2291	0.5753	0.5153	0.5437	0.3448	0.6409	0.5505	0.5923
FPNuNet-A	0.6297	0.8712	0.9171	0.8935	0.2236	0.7439	0.7066	0.7248	0.2313	0.5105	0.5243	0.5173	0.3234	0.5430	0.5459	0.5445
FPNuNet-AP	0.6691	0.9098	0.8972	0.9035	0.3657	0.7591	0.7931	0.7758	0.3334	0.6108	0.5667	0.5879	0.3776	0.5649	0.6458	0.6027
FPNuNet	**0.6894**	0.9239	0.9308	**0.9273**	0.5103	**0.8268**	0.8344	**0.8306**	**0.3823**	**0.6623**	0.5397	**0.5948**	**0.4476**	0.6093	**0.7225**	**0.6611**

All 4 foreground classes are shown; rare and morphologically ambiguous categories are merged before training (see the “Data description” section). Best in **bold**.

The ablation study within the FPNuNet family quantifies each module’s contribution to type recognition. Relative to FPNuNet-F, the adapter-only variant FPNuNet-A degrades both detection-aware type quality (mPQ: 0.3520 vs. 0.3664) and macro-level discrimination, indicating that cross-domain alignment alone does not suffice for phenotype separability. Incorporating the prompt-guided decoder in FPNuNet-AP substantially reverses this trend, lifting mPQ by *+24.0%* with concurrent improvements across all macro-averaged metrics (Table 5), confirming that explicit type supervision is essential for phenotype discrimination. The complete FPNuNet configuration yields further broad-based gains over FPNuNet-AP, most notably in mPQ (*+16.2%*) and $\mathbf {F}_{1,\mathrm{macro}}$ (*+10.3%*), consistent with the stabilizing effect of DCT-based frequency fusion on minority-class decisions. These stepwise gains align with the per-class trends in Table 6: the prompt decoder curbs cross-class confusion in crowded fields (e.g., higher $\mathbf {F}^{\mathrm{ pIm}}_{1,\mathrm{inst}}$), while the final fusion stabilizes decisions under stain heterogeneity (e.g., higher $\mathrm{PQ}^{\mathrm{ nOth}}_{\mathrm{inst}}$). Qualitatively, these trends are visible in Fig. 9: adapters reduce missed immune instances at low-contrast membranes, the prompt decoder suppresses residual subtype flips in crowded fields, and the final fusion stabilizes assignments under heterogeneous staining.

These advantages are also evident in the baseline comparison under identical training protocols (Table 5). Although HoVer-Net retains an edge in $\mathbf {P}_{w}$, this advantage is driven by the prevalent tumor class; FPNuNet’s consistently higher macro-averaged metrics indicate more balanced performance across all subtypes, including clinically relevant minority categories.

At the class level (Table 6), FPNuNet and HoVer-Net exhibit a consistent recall–precision trade-off on the dominant pTu class: FPNuNet achieves higher PQ and *F*$_1$ through stronger recall, whereas HoVer-Net retains higher precision. For pIm, FPNuNet improves precision and *F*$_1$ but falls slightly short of HoVer-Net in type-consistent $\mathrm{PQ}_{\mathrm{inst}}$, suggesting residual assignment sensitivity in crowded lymphocyte clusters. The largest gains emerge on minority subtypes: for nTu, FPNuNet substantially raises precision (0.662 vs. 0.558) and reduces false positives at tumor–non-tumor interfaces; for nOth, markedly improved recall reflects better recovery of diffuse negative non-tumor nuclei. Overall, the gains concentrate on minority and morphologically challenging subtypes (nTu, nOth) while maintaining strong pTu performance, which accounts for the improvements in macro/micro averages and mPQ. HoVer-Net’s advantage in $\mathbf {P}_{w}$ is consistent with its weighting toward the prevalent tumor class. Remaining gaps, particularly in pIm $\mathrm{PQ}_{\mathrm{inst}}$ (Table 6), indicate headroom in small-object detection and assignment (e.g., class-specific matching thresholds or small-nucleus priors) that could convert improved typing into higher panoptic quality.

We note that paired statistical tests were not applied to the classification metrics because the per-image type-level counts are sparse (several subtypes have fewer than 5 instances in individual test images), which violates the assumptions of rank-based tests and renders per-image bootstrap intervals unreliable. The reported aggregate differences should therefore be interpreted as descriptive rather than confirmatory.

Finally, the classification gains are consistent with the instance-level improvements reported above. Higher-quality instance masks and improved separation reduce label noise at object boundaries, yielding cleaner per-object features that benefit subtype discrimination. In particular, more accurate boundary localization lessens tumor–stroma contamination and improves immune typing in densely packed regions, as qualitatively illustrated in Fig. 9.

### Frequency transform ablation

To isolate the effect of frequency transform selection, we compare 4 FPNuNet variants that differ only in the transform used in the prompt generators and/or the PFAE fusion modules (Table 7). All variants share the same architecture, training schedule, and hyperparameters; only the frequency transform is varied.

**Table 7:** Frequency transform ablation on CD47-IHCNuSC.

Variant (prompt/PFAE)	AJI	bPQ	DQ	SQ
No freq. prompt/DCT	0.7136	0.7221	0.8264	**0.8738**
FFT/FFT	0.7308	0.7055	0.8214	0.8589
FFT/DCT	0.7249	0.7326	0.8439	0.8681
DCT/DCT (FPNuNet)	**0.7472**	**0.7458**	**0.8589**	0.8677

All variants share the same architecture; only the transform in the prompt generators (Prompt) and PFAE modules is varied. Best in **bold**.

The variant employing DCT in both the prompt generators and the PFAE modules (i.e., the full FPNuNet) achieves the highest AJI (0.7472) and bPQ (0.7458), followed by the variant using FFT uniformly in both stages (AJI  =  0.7308) and the baseline without frequency-based prompts (AJI  =  0.7136). The variant pairing FFT-based prompt generators with DCT-based PFAE modules attains competitive bPQ (0.7326) and DQ (0.8439), but does not surpass the consistently DCT-based configuration, suggesting that using a unified frequency transform across the prompt and fusion stages may provide more effective feature integration.

Removing the frequency-based prompt yields the lowest AJI, confirming that input-level frequency cues contribute meaningfully to instance recovery. Employing DCT uniformly in both the prompt generation and feature fusion stages achieves the best overall balance, corroborating the design choice adopted in the full FPNuNet model.

In summary, FPNuNet achieves overall performance gains on CD47-IHCNuSC by integrating DCT-based frequency-aware refinement, multi-source prompt-adapted feature extraction, and prompt-guided subtype decoding. The ablation results show that these components provide complementary contributions, particularly to subtype classification under challenging IHC staining conditions. These findings suggest that FPNuNet provides a promising framework for IHC-focused NuSC, with potential utility in tumor cellularity estimation, immune phenotyping, and biomarker-driven pathology analysis.

## Discussion

FPNuNet’s performance gains on CD47-IHCNuSC can be attributed to 2 complementary mechanisms. First, the frozen SAM and UNI backbones, adapted through lightweight DCT-based prompt generators (1.37% trainable parameters), retain generalizable structural and semantic priors while acquiring IHC-specific boundary and texture sensitivity. Second, the supplementary WFE and MSCE branches recover fine-grained spatial and frequency-domain detail that the pretrained encoders lose through repeated downsampling, and the DCT-enabled PFAE fusion neck selectively amplifies morphology-relevant spectral components. The ablation results confirm that these contributions are additive: prompt adaptation improves instance detection, the prompt-guided decoder strengthens subtype discrimination, and DCT-based fusion refines boundary calibration.

CD47-IHCNuSC fills a gap in publicly available IHC benchmarks by coupling biomarker-specific, expert-validated instance annotations with 7 fine-grained subtype labels under deliberately preserved staining heterogeneity. Two data-centric factors, however, constrain the conclusions that can be drawn. First, no formal inter-annotator agreement metric (e.g., Cohen’s *κ*) was recorded during the annotation campaign; residual label noise is therefore unquantified and likely concentrated at morphologically ambiguous boundaries and in rare subtypes, contributing to the performance ceiling on minority classes. Second, the pronounced long-tail class distribution (Table 1) is only partially addressed by the current focal-loss and background-down-weighting strategy: per-class analysis shows clear gains on nTu and nOth, yet the gap on morphologically challenging subtypes such as pIm remains substantial, suggesting that inverse-frequency weighting, class-balanced sampling, or prototype-based learning for rare categories warrant investigation.

From a computational perspective, FPNuNet trades inference speed for parameter efficiency: although it has the largest total parameter count, only 5.4 M parameters (1.37%) are trainable (Table 2), substantially reducing training memory and optimizer-state overhead. The principal deployment cost is the sequential 4-encoder forward pass, which is inherently slower than single-backbone architectures. FPNuNet is therefore best positioned as an empirical validation that frozen foundation models can be effectively adapted to specialized IHC tasks with minimal trainable overhead; lighter encoder configurations can be substituted when deployment latency is the binding constraint.

Several limitations should be acknowledged. The dataset is restricted to the CD47 biomarker in esophageal carcinoma from a single institution, which limits assessment of cross-antibody, cross-organ, and inter-institutional generalizability. External validation was not performed, and no compatible public IHC NuSC benchmark was identified; cross-dataset generalizability therefore remains untested. The test set ($N\,{=}\,16$) further limits statistical power; only large-effect comparisons meet the corrected *P*-value threshold, and narrower margins should be interpreted as directional trends. Although the annotations were expert-verified, formal inter-rater agreement was not quantified; residual label ambiguity may therefore affect performance estimates, particularly for morphologically challenging minority classes. The multi-encoder architecture increases memory footprint and latency relative to single-backbone baselines, and end-to-end whole-slide-image throughput—including tile extraction, stitching, and post-processing—was not benchmarked, precluding direct assessment of clinical deployment feasibility.

These limitations suggest several directions for future work. Extending CD47-IHCNuSC to additional biomarkers, organs, and institutions—accompanied by multi-rater annotation with explicit agreement quantification—would strengthen both the benchmark and the evidence base. Domain-adaptive fine-tuning and stain augmentation are promising avenues for improving robustness across diverse IHC staining conditions. At the application level, linking tile- and cell-level outputs to slide-level clinical endpoints such as tumor purity estimation, immune contexture quantification, and biomarker-driven risk stratification would provide a more rigorous assessment of translational utility.

## Availability of source code and requirements

Project name: FPNuNet

Project home page: https://github.com/LuluQin766/FPNuNet

Operating system(s): Linux

Programming language: Python (*≥*3.7)

Other requirements: PyTorch (*≥*1.13.0), torchvision (*≥*0.14.0), PyTorch Lightning, NumPy (*≥*1.20), SciPy (*≥*1.10), Pillow (*≥*9.4), OpenCV (*≥*4.7), scikit-learn (*≥*1.2), PyYAML (*≥*6.0), timm (*≥*0.3.2), matplotlib (*≥*3.1), tqdm, tensorboardX, onnxruntime (*≥*1.14), imageio (*≥*2.9), h5py

License: MIT License

RRID: SCR_027659

biotools: fpnunet

WorkflowHub: https://doi.org/10.48546/workflowhub.workflow.2166.1

## List of abbreviations

AJI: aggregated Jaccard index; BCE: binary cross-entropy; bPQ: binary panoptic quality; CBAM: convolutional block attention module; CD47: cluster of differentiation 47; DAB: 3,3$^\prime$-diaminobenzidine; DQ: detection quality; FFT: fast Fourier transform; FPNuNet: frequency-aware prompt-guided network; H&E: hematoxylin and eosin; HV: horizontal–vertical; IHC: immunohistochemistry; mPQ: mean panoptic quality; MSE: mean squared error; MSGE: mean squared gradient error; NuSC: nuclear segmentation and classification; PFAE: prompt-guided frequency-aware enhancement; PQ: panoptic quality; SAM: Segment Anything Model; SQ: segmentation quality; TMA: Tissue Microarray; UNI: universal pathology foundation model; ViT: Vision Transformer; WSI: whole-slide image.

## Supplementary Material

giag091_Authors_Response_To_Reviewer_Comments_original_submission

giag091_GIGA-D-25-00479_original_submission

giag091_GIGA-D-25-00479_revision_1

giag091_Reviewer_1_Report_original_submission Reviewer 1 -- 1/5/2026

giag091_Reviewer_1_Report_revision_1 Reviewer 1 -- 6/8/2026

giag091_Reviewer_2_Report_original_submission Reviewer 2 -- 2/1/2026

giag091_Reviewer_2_Report_revision_1 Reviewer 2 -- 7/5/2026

## Data Availability

The CD47-IHCNuSC dataset—including image patches, instance-level masks, nuclear type maps, and metadata—is available in the GigaScience repository, GigaDB [41], under the dataset entry [42]. The DOME-ML annotations for this work can be found in the DOME Registry [43].
