# Supplementary material for: FPNuNet: a frequency-aware prompt-guided network for nuclear segmentation and classification in immunohistochemistry images
Source: Gigascience. 2026 Sep 7;15:giag091. doi: 10.1093/gigascience/giag091 (PMC13618258; doi:10.1093/gigascience/giag091)
Supplement: Supplementary file 1 [file giag091_authors_response_to_reviewer_comments_original_submission.pdf]

Author's Response To Reviewer Comments

Dear Editor and Reviewers,

We sincerely thank the Editor and both Reviewers for their careful and thorough evaluation of our manuscript. The critiques are substantive and have considerably sharpened our thinking about the scope and limitations of this work. We are particularly grateful for the Reviewers' recognition of the dataset's value to the community, and we regard the methodological concerns—regarding baseline fairness, statistical rigor, architectural justification, and dataset accessibility—as precisely the kind of constructive challenge that strengthens published science.

In this revision, we have addressed every comment with one of three approaches: (1) corrected or expanded manuscript text where the existing evidence was sufficient but the writing was unclear or misleading; (2) new experimental evidence where additional data were available; and (3) honest acknowledgment of limitations where the current study cannot fully resolve a concern.

All section/table references below refer to the revised manuscript.

Editor Requirements

EQ1. Register the software on bio.tools and SciCrunch.org to obtain an RRID and a biotoolsID, and cite these in the manuscript.

Response: Done. FPNuNet is registered on SciCrunch (RRID: SCR_027659) and bio.tools (biotools:fpnunet). Both identifiers are cited in the revised "Availability of Source Code and Requirements" section.

EQ2. Register the computational workflow on workflowhub.eu and cite the DOI in the manuscript.

Response: Done. The analysis workflow is deposited on WorkflowHub (DOI: 10.48546/workflowhub.workflow.2166.1). The DOI is cited in the revised "Availability of Source Code and Requirements" section.

EQ3. Submit a point-by-point response in the "Response to Reviewers" box.

Response: This document constitutes the point-by-point response.

EQ4. If data or code have been modified, update the public repository.

Response: The public GitHub repository (https://github.com/LuluQin766/FPNuNet) has been updated to include corrected model descriptions, evaluation scripts, and the CD47-IHCNuSC dataset with train/test split identifiers.

EQ5. Ensure the revised manuscript follows all journal format requirements.

Response: We have verified compliance with the GigaScience author guidelines. The revised manuscript conforms to the required structure, reference style, and formatting.

Response to Reviewer #1

We are grateful to Reviewer #1 for a highly structured and technically demanding review. The five major concerns—cross-domain generalization, baseline fairness, dataset accessibility, annotation quality control, and ablation granularity—each identify a genuine gap between the paper's claims and the evidence provided. We address them in full below.

R1-Q1: Generalization beyond a single biomarker and tumor site
The dataset contains only a single biomarker (CD47) and a single tumor site, with a limited number of WSIs and patches. The review requires: (a) external validation on different cohorts/institutions/scanners; (b) a clear distinction between in-dataset performance and cross-domain generalization claims.

Response: We thank the reviewer for raising this important concern. We agree that the current dataset is limited to one biomarker and one tumor site, and therefore does not support broad cross-domain generalization claims.
What we have done:
1. We revised the Abstract, Introduction, Results, and Discussion to clearly restrict our performance claims to the CD47-IHCNuSC benchmark and to avoid implying generalization across biomarkers, tumor types, institutions, or scanners.
2. We examined publicly available IHC nuclear analysis datasets for possible external validation. However, most existing IHC resources are either not publicly accessible, provide only server-side evaluation, or are designed for different tasks such as point-level detection, binary positivity as-sessment, or H&E-based nuclear segmentation. To the best of our knowledge, no public IHC dataset currently provides instance-level nuclear segmentation masks with subtype-level classifi-cation labels compatible with the NuSC evaluation protocol used in this study.
3. We therefore acknowledge the lack of external validation as a major limitation rather than presenting CD47-IHCNuSC results as evidence of cross-domain robustness. The Discussion has been revised to explicitly state that future validation should include multi-institutional cohorts, additional scanners, additional biomarkers, and other tumor types.
4. We also added future directions on domain-adaptive fine-tuning, stain augmentation, and the ex-tension of CD47-IHCNuSC to broader IHC settings.
Accordingly, the revised manuscript positions CD47-IHCNuSC as an initial benchmark for IHC-based nuclear segmentation and classification, rather than as evidence of universal generalization across IHC domains.
[Manuscript change] Revised Abstract (scope qualification); new Limitations paragraph on generalization in Discussion; honest acknowledgment that cross-dataset validation is precluded by the current unavailability of compatible public IHC NuSC benchmarks.

R1-Q2: Baseline comparison fairness
Some baselines use publicly pretrained weights without fine-tuning, while other models use the paper's training protocol. This creates an unfair comparison.

Response: This concern is well-founded and overlaps with R2-Q5.
What we have done:
1. In the revised manuscript, we have retrained HoverFast and Cellpose-SAM on the CD47-IHCNuSC training split under the same training protocols as FPNuNet, HoVer-Net, and SRSA-Net. Only StarDist remains evaluated with its publicly released pretrained model as an off-the-shelf instance segmentation baseline, because its TensorFlow backend is incompatible with our PyTorch training pipeline.
2. The revised Experimental Setup now explicitly states the training condition for each baseline (Section 3.1): "the FPNuNet series, HoVer-Net, SRSA-Net, HoverFast, and Cellpose-SAM were trained on the same CD47-IHCNuSC training split using consistent training and evaluation protocols whenever applicable. StarDist was evaluated with its publicly released pretrained model."
3. StarDist's training condition is transparently stated in the Experimental Setup; the large performance gap (e.g., AJI 0.450 vs. FPNuNet's 0.747) reflects both architectural limitations and the absence of domain adaptation, and does not affect the ranking among fine-tuned methods.
[Manuscript change] Experimental Setup: HoverFast and Cellpose-SAM retrained on CD47-IHCNuSC; explicit training-condition statement for each baseline.

R1-Q3: Reproducibility and data access
The dataset was private during review. The review requires: preprocessing/slicing code, exact train/test split identifiers, evaluation code, and a clear public release plan.

Response: We apologize for the dataset not being publicly accessible during the initial review. The CD47-IHCNuSC dataset—including image patches, instance-level masks, nuclear type maps, and metadata—is

now publicly hosted in the project repository: https://github.com/LuluQin766/FPNuNet/tree/main/CD47_IHCNUSC. Train/test split identifiers and evaluation scripts are included.
[Manuscript change] Revised "Data Availability" section with public repository URL.

R1-Q4: Annotation reliability and rare-class error patterns
Human-in-the-loop annotation is described but no quantitative quality control is provided. The review requires: inter-annotator agreement metrics, adjudication rate, error pattern analysis for rare classes, and analysis of class imbalance effects.

Response: The Reviewer correctly identifies that the annotation protocol description lacks quantitative grounding.
What we have done:
1. The Methods section (Section 2.1) describes the full annotation pipeline: HoverFast-based prelabeling, manual refinement in QuPath by trained scientists, and adjudication by board-certified pathologists.
2. A new "Annotation quality" paragraph in the Discussion transparently acknowledges that no formal inter-annotator agreement (e.g., Cohen's κ) or adjudication-rate statistics were recorded during the annotation campaign. We identify this as a limitation and recommend multi-rater protocols with explicit agreement quantification for future annotation efforts.
3. Residual label noise is discussed as most likely concentrated at morphologically ambiguous boundaries (immune-related vs. stromal nuclei) and in rare subtypes, contributing to the performance ceiling on minority classes.
4. The effects of class imbalance are addressed in a dedicated "Class imbalance" paragraph in the Discussion (see also R2-Q1).
[Manuscript change] Discussion: new "Annotation quality" paragraph acknowledging the absence of quantitative inter-annotator agreement data; new "Class imbalance" paragraph with per-class analysis.

R1-Q5: Ablation clarity and complexity justification
The frequency (DCT/wavelet) and prompt components are complex. The ablation is insufficiently fine-grained. The review requires: targeted ablations isolating each component; statistical confidence across slides; runtime/memory/latency analysis.

Response: We agree that the original ablation presentation was insufficient.
What we have done:
1. A new progressive ablation table (Table 4) traces the contribution of each module (Adapter, Type Decoder, DCT-PFAE/SkipNeck). The Results text explicitly acknowledges that the final step couples three changes and notes this as a limitation.
2. A new Frequency Transform Ablation subsection (Section 3.5, Table 7) isolates the effect of trans-form selection by comparing four variants on CD47-IHCNuSC.
3. Table 2 reports THOP-measured FLOPs and parameter counts for all baselines with available profiling (HoverFast, HoVer-Net, Cellpose-SAM, FPNuNet). Latency and memory are reported in the accompanying prose. A revised "Computational complexity" paragraph in the Discussion frames FPNuNet as a parameter-efficient adaptation framework.
4. Statistical power limitations ($N=16$) are addressed in a new Discussion paragraph (see R2-Q2).
5. To provide the requested statistical confidence across slides, we now report paired per-image bootstrap 95% confidence intervals (10,000 resamples) and Holm–Bonferroni-corrected Wilcoxon signed-rank tests for all pairwise comparisons in the Results (Section 3.3). On AJI, FPNuNet significantly outperforms every baseline and ablation variant (corrected $p < 0.05$). Corresponding
CIs and p-values are reported for bPQ, SQ, DQ, Dice1, and Dice2, with non-significant differences explicitly acknowledged (e.g., bPQ vs. HoVer-Net: $p = 0.28$).
[Manuscript change] New Table 4 (progressive ablation); new Table 7 (frequency transform ablation); Table 2 (model complexity for four baselines); DCT coupling paragraph in Results; revised "Computational complexity" paragraph in Discussion; new paired statistical analysis (bootstrap CI + Wilcoxon) in Results Section 3.3.

Response to Reviewer #2

We thank Reviewer #2 for a rigorous and technically precise review. While affirming the dataset's value,

Reviewer #2 raises pointed concerns regarding statistical credibility, architectural proportionality, and a factual error in a complexity claim. We accept several of these criticisms unreservedly and address each below.

R2-Q1: Class imbalance insufficiently addressed
Severe class imbalance (pTu 40.5%, multiple classes <1%) is not sufficiently addressed. Only 4 of 7 classes are reported in Table 3. Standard focal loss cannot fundamentally solve such severe imbalance.

Response: Reviewer #2 is correct on all counts. We acknowledge that focal loss alone cannot adequately address imbalance of this magnitude, and the omission of three classes from Table 3 was a significant oversight that we have now corrected.
Why only 4 of 7 classes were originally reported: Three categories (pOth, nOth, nSt) are either extremely rare or morphologically ambiguous, providing insufficient test-set representation for reliable per-class evaluation. They are merged into pTu and nTu prior to training, reducing the taxonomy to four foreground classes. The original manuscript did not describe this merging decision, which we acknowledge as an oversight.
What we have done:
1. The revised Methods section (Section 2.2) now explicitly describes the class merging, its rationale, and the resulting four-class taxonomy.
2. The loss configuration (focal $\gamma=2.0$, uniform foreground weights, no inverse-frequency weighting) is stated explicitly.
3. A new per-class classification table (Table 6) reports PQ/P/R/F1 for all four foreground subtypes across all models. Table 5 is updated accordingly.
4. A new "Class imbalance" paragraph in the Discussion analyses per-class trends, acknowledges remaining gaps on minority subtypes, and identifies inverse-frequency weighting and prototype-based learning as future directions.
[Manuscript change] Revised Methods (class merging + loss specification); new per-class Table 6 with four foreground classes; updated Table 5 (overall metrics); new "Class imbalance" paragraph in Discussion.

R2-Q2: No statistical testing, N=16
There is no statistical testing with only 16 test images. Modest performance improvements may reflect random variation.

Response: We agree that N=16 constitutes a small test set and that modest performance differences may reflect random variation.
What we have done:
1. We performed paired statistical testing based on per-image metric values. Pairwise comparisons between FPNuNet and each competing method were assessed using Wilcoxon signed-rank tests with Holm–Bonferroni correction for multiple comparisons (Results, Section 3.3).
2. Performance claims in the Abstract, Introduction, and Results have been qualified to reflect the limited sample size. Where margins are small or statistically non-significant, observed differences are framed as trends rather than definitive rankings.
[Manuscript change] Paired Wilcoxon signed-rank tests with Holm–Bonferroni correction added to Results Section 3.3; revised "Statistical power" paragraph in the Discussion; scope-qualified performance claims throughout.

R2-Q3: Complexity vs. performance gain
Limited performance gains relative to large architectural complexity increase. Paper acknowledges memory and latency issues but lacks quantitative analysis.

Response: This is a substantive architectural critique, and we accept its core premise.
What we have done:
1. Table 2 reports THOP-measured FLOPs and parameter counts for all profiled baselines; latency and memory are discussed in the accompanying prose (see also R1-Q5).
2. The revised "Computational complexity" paragraph in the Discussion frames FPNuNet as a parameter-efficient adaptation framework rather than a lightweight inference model, and suggests smaller pretrained encoders as a path to faster deployment.

3. Regarding cross-dataset evaluation, please see our detailed response in R1-Q1: despite extensive efforts, no publicly available IHC dataset with compatible instance-level NuSC annotations could be identified. This limitation is acknowledged in the revised Discussion.
[Manuscript change] Table 2 (model complexity for four baselines); revised "Computational complexity" paragraph in Discussion; revised Limitations paragraph acknowledging the absence of cross-dataset validation.

R2-Q4: DCT vs. FFT complexity claim is incorrect
The claim "DCT provides real-valued frequency representation without FFT complexity" is factually wrong—both are O(n log n). FFT may be faster. Non-DCT changes may be the actual performance source.

Response: We accept this correction unreservedly. The original statement was factually incorrect and should not have appeared in the manuscript.
What we have done:
1. The incorrect complexity claim has been removed. The revised Frequency-Aware Fusion Modules description (Section 2.2) now justifies the DCT choice on two accurate grounds: (a) energy compaction—DCT concentrates spectral energy of smooth, localized structures into fewer low-frequency components, providing a compact spectral basis for IHC nuclear textures; and (b) real-valued coefficients—DCT eliminates the complex-number bookkeeping (magnitude/phase storage, complex-to-real conversion) that FFT would impose, simplifying the gradient path. These are implementation and representation advantages, not computational complexity reductions.
2. The revised Methods paragraph describes DCT's role uniformly at both the encoder adaptation and post-encoder fusion stages, with empirical validation of this design choice in the Frequency Transform Ablation (Section 3.5).
3. A new Frequency Transform Ablation subsection (Section 3.5, Table 7) isolates the effect of trans-form selection by comparing four variants on CD47-IHCNuSC, confirming the DCT choice empirically.
4. The Results text acknowledges that the final ablation step couples three changes and notes this as a limitation (see also R1-Q5).
[Manuscript change] Corrected PFAE Block text in Methods (Section 2.2); new frequency processing overview paragraph (Section 2.2); Table 4 (progressive ablation); new Table 7 (frequency transform ablation, Section 3.5); new DCT coupling discussion paragraph in Results.

R2-Q5: Baseline comparison not apples-to-apples
CellPose and similar models were not fine-tuned on the authors' dataset. Even without fine-tuning, FPNuNet's advantage is marginal.

Response: This concern overlaps directly with R1-Q2, and we accept the criticism.
What we have done:
1. In the revised manuscript, HoverFast and Cellpose-SAM have been retrained on the CD47-IHCNuSC training split using the same protocols as the other baselines. Only StarDist remains evaluated with its pretrained weights (TensorFlow backend incompatibility).
2. The revised Experimental Setup now explicitly states the training condition for each baseline.
3. StarDist's training condition is transparently stated in the Experimental Setup; the large performance gap does not affect conclusions among fine-tuned methods.
See R1-Q2 for full details.
[Manuscript change] Same as R1-Q2.

Cookie Preference Center

We use cookies which are necessary to make our site work. We may also use additional cookies to analyze, improve and personalize our content and your digital experience. For more information, see our Cookie Policy.

You may choose not to allow some types of cookies. However, blocking some types may impact your experience of our site and the services we are able to offer. See the different category headings below to find out more or change your settings.

Allow all

Manage Consent Preferences

Strictly Necessary Cookies

Always active

These cookies are necessary for the website to function and cannot be switched off in our systems. They are usually only set in response to actions made by you which amount to a request for services, such as setting your privacy preferences, logging in or filling in forms. You can set your browser to block or alert you about these cookies, but some parts of the site will not then work.

Functional Cookies

These cookies enable the website to provide enhanced functionality and personalisation. They may be set by us or by third party providers whose services we have added to our pages. If you do not allow these cookies then some or all of these services may not function properly.

Performance Cookies

These cookies allow us to count visits and traffic sources so we can measure and improve the performance of our site. They help us to know which pages are the most and least popular and see how visitors move around the site.

Targeting Cookies

These cookies may be set through our site by our advertising partners. They may be used by those companies to build a profile of your interests and show you relevant adverts on other sites. If you do not allow these cookies, you will experience less targeted advertising.

Cookie List

Clear

Apply Cancel

Consent Leg.Interest

Confirm my choices

Powe
