# Supplementary material for: FPNuNet: a frequency-aware prompt-guided network for nuclear segmentation and classification in immunohistochemistry images
Source: Gigascience. 2026 Sep 7;15:giag091. doi: 10.1093/gigascience/giag091 (PMC13618258; doi:10.1093/gigascience/giag091)
Supplement: Supplementary file 2 [file giag091_giga-d-25-00479_original_submission.pdf]

GigaScience

FPNuNet: A Frequency-Aware Prompt-Guided Network for Nuclear Segmentation and Classification in Immunohistochemistry Images

--Manuscript Draft--

**Manuscript Number:**	GIGA-D-25-00479
**Full Title:**	FPNuNet: A Frequency-Aware Prompt-Guided Network for Nuclear Segmentation and Classification in Immunohistochemistry Images
**Article Type:**	Research
**Funding Information:**	National Key Technologies Research and Development Program of China (2022YFF1202104) — Prof. Zexuan Zhu<br>National Natural Science Foundation of China (62471310) — Prof. Zexuan Zhu
**Abstract:**	Accurate nuclear segmentation and classification (NuSC) of immunohistochemistry (IHC)-stained whole-slide images are essential for reliable biomarker quantification in computational pathology. While NuSC methods excel with Hematoxylin and Eosin images, they frequently underperform on IHC-stained images due to domain-specific challenges such as stain heterogeneity, low nuclear contrast, and the lack of biomarker-specific annotations. Prior attempts leveraging stain normalization or cross-domain knowledge transfer have yielded limited improvements under these conditions. To address these limitations, we introduce FPNuNet, a frequency-aware prompt-guided network tailored for robust NuSC of IHC-stained images. FPNuNet first employs a color fusion stem to integrate RGB and Hematoxylin-Eosin-Diaminobenzidine channels, enhancing feature representation across diverse staining variations. This enriched input is jointly processed by a segment-anything-model-based structural encoder and a vision-transformer-based semantic encoder pretrained on large-scale pathology data, with both encoders modulated via lightweight prompt adapters. A progressive frequency-aware residual global fusion neck then is applied to aggregate multi-scale and frequency-sensitive features. Finally, three collaborative decoder branches are jointly used to predict the binary masks, horizontal-vertical vectors, and nuclear types. FPNuNet is evaluated on a CD47-IHCNuSC dataset that comprises 86 CD47-stained esophageal cancer patches with 18,494 manually annotated nuclei spanning seven clinically meaningful subtypes. Experimental results demonstrate that FPNuNet consistently outperforms state-of-the-art baselines in both segmentation accuracy and classification robustness, particularly under challenging IHC staining conditions.
**Corresponding Author:**	Zexuan Zhu<br>Shenzhen University<br>Shenzhen, CHINA
**Corresponding Author Secondary Information:**	
**Corresponding Author's Institution:**	Shenzhen University
**Corresponding Author's Secondary Institution:**	
**First Author:**	Lulu Qin
**First Author Secondary Information:**	
**Order of Authors:**	Lulu Qin<br>Zhigang Pei<br>Xudong He<br>Jiarui Zhou<br>Xianhong Xu

Powered by Editorial Manager® and ProduXion Manager® from Aries Systems Corporation

	Zexuan Zhu
**Order of Authors Secondary Information:**	
**Additional Information:**	
**Question**	**Response**
Are you submitting this manuscript to a special series or article collection?	No
**Experimental design and statistics**<br><br>Full details of the experimental design and statistical methods used should be given in the Methods section, as detailed in our Minimum Standards Reporting Checklist. Information essential to interpreting the data presented should be made available in the figure legends.<br><br>Have you included all the information requested in your manuscript?	Yes
**Resources**<br><br>A description of all resources used, including antibodies, cell lines, animals and software tools, with enough information to allow them to be uniquely identified, should be included in the Methods section. Authors are strongly encouraged to cite Research Resource Identifiers (RRIDs) for antibodies, model organisms and tools, where possible.<br><br>Have you included the information requested as detailed in our Minimum Standards Reporting Checklist?	Yes
**Availability of data and materials**<br><br>All datasets and code on which the conclusions of the paper rely must be either included in your submission or deposited in publicly available repositories (where available and ethically appropriate), referencing such data using a unique identifier in the references and in	Yes

the “Availability of Data and Materials” section of your manuscript.<br><br>Have you have met the above requirement as detailed in our Minimum Standards Reporting Checklist?	
GigaScience has policies and guidelines in place for the use of generative AI-writing tools such as ChatGPT. If you have used such writing tools to assist with writing the manuscript this must be declared and cited in the text. Authors should not list AI-writing tools and other AI-assisted technologies as an author or co-author and should acknowledge that they are fully responsible for text generated or refined by AI-writing tools.<p><br><br>A summary of use (particularly in the introduction or among methods) needs to be included at the end of the paper, and the outputs should also be included as a supplementary file hosted in GigaDB or other open repositories. Please read our guidelines for more information. <p><br><br>By submitting to GigaScience, you are aware of the journal's AI-writing tools policy, and if you have declared use of such tools below, you have acknowledged this where appropriate in your manuscript and have made a summary of use and outputs available. <p><br><b>AI-assisted writing tools have been used in the preparation of this manuscript?	No

Powered by Editorial Manager® and ProduXion Manager® from Aries Systems Corporation

Manuscript
Click here to access/download;Manuscript;FPNuNet-gigascience-v8.pdf

Placeholder for OUP logo oup.pdf

(GIGA)n SCIENCE

GigaScience, 2025, 1–13

doi: xx.xxxx/xxxx
Manuscript in Preparation
Paper

PAPER

FPNuNet: A Frequency-Aware Prompt-Guided Network for Nuclear Segmentation and Classification in Immunohistochemistry Images

Lulu Qin[1], Zhigang Pei[2], Xudong He[3], Jiarui Zhou[4], Xianhong Xu[5] and Zexuan Zhu[3,*]

[1]College of Computer Science and Software Engineering, Shenzhen University, China and [2]Pathology Department, Chongqing University Jiangjin Hospital, China and [3]School of Artificial Intelligence, Shenzhen University, China and [4]School of Biosciences, University of Birmingham, UK and [5]Department of Oncology, University of Oxford, UK

*Corresponding author. zhuzx@szu.edu.cn

Abstract

Accurate nuclear segmentation and classification (NuSC) of immunohistochemistry (IHC)-stained whole-slide images are essential for reliable biomarker quantification in computational pathology. While NuSC methods excel with Hematoxylin and Eosin images, they frequently underperform on IHC-stained images due to domain-specific challenges such as stain heterogeneity, low nuclear contrast, and the lack of biomarker-specific annotations. Prior attempts leveraging stain normalization or cross-domain knowledge transfer have yielded limited improvements under these conditions. To address these limitations, we introduce FPNuNet, a frequency-aware prompt-guided network tailored for robust NuSC of IHC-stained images. FPNuNet first employs a color fusion stem to integrate RGB and Hematoxylin-Eosin-Diaminobenzidine channels, enhancing feature representation across diverse staining variations. This enriched input is jointly processed by a segment-anything-model-based structural encoder and a vision-transformer-based semantic encoder pretrained on large-scale pathology data, with both encoders modulated via lightweight prompt adapters. A progressive frequency-aware residual global fusion neck then is applied to aggregate multi-scale and frequency-sensitive features. Finally, three collaborative decoder branches are jointly used to predict the binary masks, horizontal-vertical vectors, and nuclear types. FPNuNet is evaluated on a CD47-IHCNuSC dataset that comprises 86 CD47-stained esophageal cancer patches with 18,494 manually annotated nuclei spanning seven clinically meaningful subtypes. Experimental results demonstrate that FPNuNet consistently outperforms state-of-the-art baselines in both segmentation accuracy and classification robustness, particularly under challenging IHC staining conditions.

Key words: Medical Imaging; Computational Pathology; Nuclear Segmentation and Classification; Immunohistochemistry; Frequency-Aware Fusion

Introduction

Automated histopathology is reshaping clinical workflows by enabling reproducible, scalable biomarker quantification and large-cohort analysis [1, 2]. Robust nuclear segmentation and classification (NuSC) is central to these pipelines, underpinning cell-level readouts (e.g., counts, densities, and phenotypes) that inform diagnosis, prognosis, and treatment monitoring [3]. Yet clinical slide heterogeneity and lab-specific protocols demand NuSC models that remain accurate across staining variability and acquisition differences [4, 5, 6].

Deep learning has driven rapid advances in NuSC on Hematoxylin and Eosin (H&E) slides, enabled by sustained algorithmic innovation and the standardization of evaluation on widely

Compiled on: November 17, 2025.
Draft manuscript prepared by the author.

Key Points

- A frequency-aware, prompt-guided dual-encoder framework namely FPNuNet is proposed for robust nuclear segmentation and classification in IHC-stained pathology images.
- A new benchmark dataset, CD47-IHCNuSC, is curated for biomarker-aware analysis of CD47-stained esophageal carcinoma.
- Comprehensive evaluations show that FPNuNet achieves superior segmentation and classification performance, outperforming state-of-the-art baselines under challenging IHC conditions.

adopted public benchmarks. Representative architectures like HoVer-Net [7] unifies pixel-wise masks with horizontal–vertical distance regression to split touching instances while enabling joint nuclear typing. TransUNet [8] combines a transformer encoder with a U-Net style decoder to fuse long-range dependencies with multi-scale detail. Triple U-Net [9] adds hematoxylin-aware pathways and progressive dense aggregation to stabilize boundaries and reduce type confusion. Swin-UNet [10] employs hierarchical windowed self-attention with shifted windows to capture multi-scale context efficiently. Collectively, these designs deliver reliable instance delineation and nuclear typing on H&E slides, yet they are calibrated to H&E-specific stain appearance and morphology, which hinders direct carry-over to immunohistochemistry (IHC) stained images [11]. IHC introduces heterogeneous diaminobenzidine (DAB) deposition that distorts local spectra and weakens the hematoxylin counterstain, degrading edges and intra-nuclear contrast. Antibody and protocol variability reshapes texture and class balance, amplifying ambiguity among positive, weak-positive, and negative phenotypes. These shifts induce characteristic errors—boundary leakage, over- or under-segmentation, and unstable typing—motivating the IHC-specific remedies introduced next.

Recent research on IHC-oriented NuSC has converged on two directions. The first learns directly on IHC with coupled objectives so that segmentation and phenotype or intensity estimation co-adapt within a single model. For example, IHC-Net [12] combines a fully convolutional network segmenter with watershed splitting and a classical ensemble for intensity scoring on estrogen receptor IHC. SRSA-Net [13] modernizes the triple-head design with a lightweight encoder, decoder-level self-attention, and seamless stitching and also provides public assets. PH-ScoreNet [14] uses dual branches and dual attention to predict IHC-scores end-to-end for proliferating cell nuclear antigen. The second reduces the stain gap prior to segmentation through generative translation or morphology normalization. For instance, GeNSeg-Net [15] performs in-domain generative adversarial network (GAN) based enhancement followed by a lightweight U-Net and watershed, achieving strong cross-tissue transfer. NuclSeg [16] bridges from IHC to multiplex immunofluorescence images using semi-supervised stain deconvolution with multi-GAN fusion and thus depends on co-registered pairs. UPHGAN [17] translates IHC to virtual H&E with a U-Net–PatchGAN generator–discriminator trained with a Huber-augmented objective and then fuses masks from both domains. The work [18] similarly translates IHC to virtual H&E to unlock H&E-specialized segmenters such as HoVer-Net, while offering limited gains for cross-modal models like StarDist [18]. ISC-GAN integrates CycleGAN with Mask R-CNN in a curriculum to adapt from labeled H&E to unlabeled IHC [19]. A pragmatic variant leverages IHC primarily for supervision rather than inference by using destain–restain registration to transfer IHC-derived labels back to H&E, thereby improving immune-cell recognition [20]. Despite tangible gains from task coupling, in-domain enhancement, and cross-stain supervision, progress is still limited by the lack of publicly accessible, biomarker-specific IHC benchmarks with subtype-level NuSC labels and by the scarcity of architectures explicitly tailored to heterogeneous IHC staining.

Another major obstacle to progress is the paucity of high-quality, publicly available, and expertly annotated IHC datasets [3]. While established benchmarks such as CoNSeP [7], MoNuSAC [21], and PanNuke [22] have catalyzed advances in H&E-based NuSC, they do not capture IHC-specific complexity—heterogeneous 3,3′-diaminobenzidine deposition, diminished nuclear contrast, and the absence of biomarker context. By contrast, IHC resources remain fragmented, i.e., most widely cited datasets are private or request-only [12, 23, 15, 16, 20, 14], and others are image-only test corpora without publicly released ground truth, with scoring available only via server-side submission [18, 17]. Even where labels exist, they are often restricted to binary positivity rather than per-nucleus subtypes and frequently lack fixed splits and standardized tiling/stitching protocols, complicating fair instance-level evaluation and cross-study replication [17, 18]. Taken together, the field still lacks publicly accessible, expert-verified IHC benchmarks with subtype-level annotations and standardized evaluation protocols, suitable for rigorous, reproducible NuSC on IHC.

To address the aforementioned issues and facilitate biomarker-aware NuSC under the severe stain variability of IHC, we present FPNuNet a frequency-aware, prompt-guided network that couples a segment-anything-model (SAM) -based structural encoder [24] with a vision transformer (ViT) based semantic encoder UNI [25] and refines their outputs through progressive frequency-enhanced fusion, as shown in Fig. 1. FPNuNet was validated on the IHC-stained images of a representative biomarker namely the tumor-associated antigen *cluster of differentiation 47* (CD47), whose overexpression enables tumour cells to evade macrophage-mediated phagocytosis and is correlated with aggressive behaviour and poor prognosis in oesophageal carcinoma and other malignancies [26, 27, 28]. To support this task, we curate CD47-IHCNuSC, a publicly available, expert-annotated benchmark for CD47-stained oesophageal carcinoma. Extensive experiments on CD47-IHCNuSC and multiple H&E benchmarks demonstrate that FPNuNet delivers state-of-the-art accuracy and superior cross-stain generalisation, establishing a new foundation for IHC-centric computational pathology. The main contributions of this work are highlighted as follows:

- A frequency-aware prompt-guided architecture namely FPNuNet is proposed for NuSC of IHC-stained images. FPNuNet is featured by a SAM structural encoder, a UNI pathology encoder, a Progressive Frequency-Aware Residual Refinement (PFAR)-based fusion neck, and tri-branch decoders for simultaneous binary, horizontal-vertical (HV), and type predictions
- The first IHC dataset CD47-IHCNuSC dedicated to CD47-stained oesophageal cancer is released for public access. The dataset comprise 86 high-resolution patches (18,494 nuclei across seven clinically meaningful subtypes) annotated via a semi-automated pretrained model-assisted protocol and expert verification.
- Systematic experiments were conducted to demonstrate the effectiveness of FPNuNet. It improves Panoptic Quality by up to 6.3 percentage points in the comparison with other state-of-the-art methods, establishing a strong foundation for IHC-

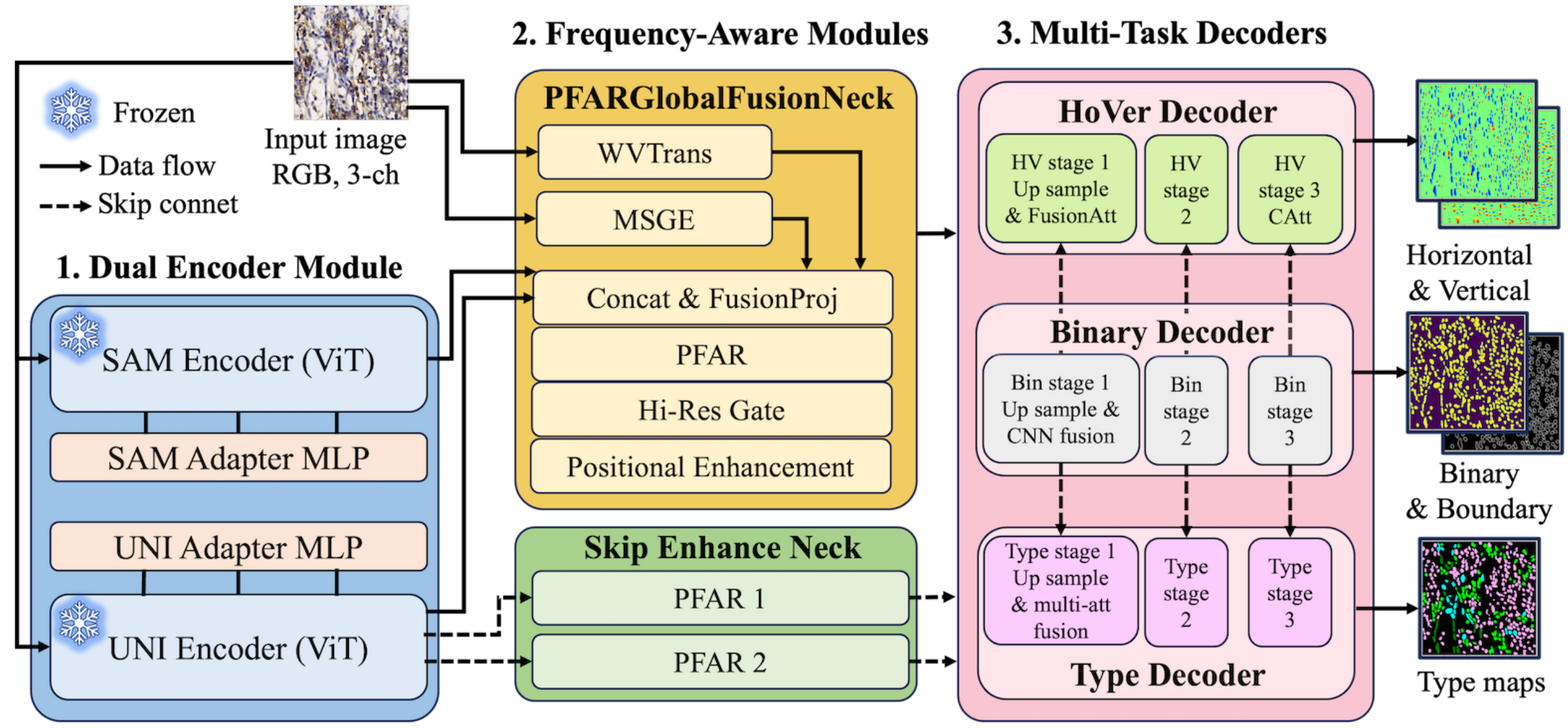

Figure 1. Architecture of the proposed FPNuNet.

specific NuSC.

Materials and Methods

Data Description

To enable rigorous evaluation of biomarker-aware NuSC under clinically realistic IHC conditions, we construct **CD47-IHCNuSC**, a new benchmark for the representative CD47-stained esophageal cancer. Among publicly accessible resources, label granularity is typically limited: many provide detection only or binary typing—for example, BCData [32] offers point detection with two classes, DeepLIIF [33] provides registered IHC-multiplex-immunofluorescence tiles with binary Ki-67 status, and SRSA-Net [13] supplies instance masks with positive/negative labels. Other efforts emphasize stain-domain robustness rather than fine-grained nuclear subtyping, and several influential works remain non-public or prioritize intensity scoring over subtype taxonomy [12, 23, 34]. **CD47-IHCNuSC** addresses these limitations by targeting esophageal tissue and the CD47 biomarker, providing expert-validated instance masks with seven fine-grained, phenotype-aware nuclear subtypes under realistic IHC variability.

Construction, Annotation, and Quality Control

All CD47-immunostained esophageal whole-slide images (WSIs) were processed using a diaminobenzidine chromogen with hematoxylin counterstain and digitized at 0.25 μm/pixel (40$\times$) on an Aperio AT2 scanner. A board-certified pathologist delineated tumor-enriched, morphologically diverse regions; patches with low tissue content were excluded. We sampled **86** non-overlapping 1024 $\times$ 1024 patches (see Table 1 for image counts and class distributions). To preserve real-world appearance, no stain normalization was applied.

To generate instance-level and type-level annotations, a semi-automated human-in-the-loop pipeline was adopted as depicted in Fig. 2. Coarse nuclear masks were initially generated using HoverFast [36], a publicly available segmentation model that includes a variant pretrained for nuclear semantic segmentation on IHC-stained images. These preliminary masks were refined through multiple rounds of manual correction in QuPath through split/merge operations and boundary correction. Trained scientists assigned preliminary subtypes; board-certified pathologists adjudicated ambiguities and harmonized criteria. Final annotations are released in standardized formats—OME-TIFF for images and mat for instance masks—to ensure reproducibility and compatibility with downstream pipelines.

Statistics, Benchmark Characteristics, and Availability

The final dataset comprises **18,483** quality-controlled nuclei assigned to **seven** histologically and biomarker-informed subtypes spanning CD47-positive/negative tumor, immune, stromal, and rare/other categories (Table 1; Fig. 2). The distribution exhibits a pronounced long tail: positive-tumor and immune nuclei dominate, whereas rare categories (e.g., negative-immune and stroma) are sparsely represented. Beyond class imbalance, the benchmark captures three key challenges: (i) stain heterogeneity (Diaminobenzidine intensity and counterstain variability), (ii) nuclear crowding and adhesion in tumor regions, and (iii) shape and size diversity across tissue contexts. Collectively, these factors stress a model's ability to jointly delineate instances and infer biomarker-sensitive types under real-world clinical heterogeneity.

CD47-IHCNuSC complements the current breast-centric datasets [12, 23, 32, 33] and multiplex Tissue Microarray corpora [13] by broadening biomarker and organ coverage to esophageal IHC with fine-grained nuclear subtyping. The combination of biomarker specificity, fine-grained nuclear categorization, real-world stain variability, and expert-reviewed annotations makes CD47-IHCNuSC a challenging and representative benchmark. It provides a valuable resource for training and evaluating segmentation models under clinically realistic IHC conditions. In the following section, we validate the proposed FPNuNet and existing baselines on CD47-IHCNuSC to demonstrate its utility and difficulty as a benchmark for biomarker-aware NuSC.

FPNuNet

As illustrated in Fig. 1, FPNuNet comprises three key components, i.e., 1) a dual-encoder module for structural and semantic feature extraction, 2) frequency-aware fusion modules for multi-features fusion and enhancement, and 3) a multi-task decoders for collaborative prediction. The dual encoders consists of a SAM-based structural encoder pretrained on natural images to capture fine-grained boundary details, and a UNI-based semantic encoder pre-

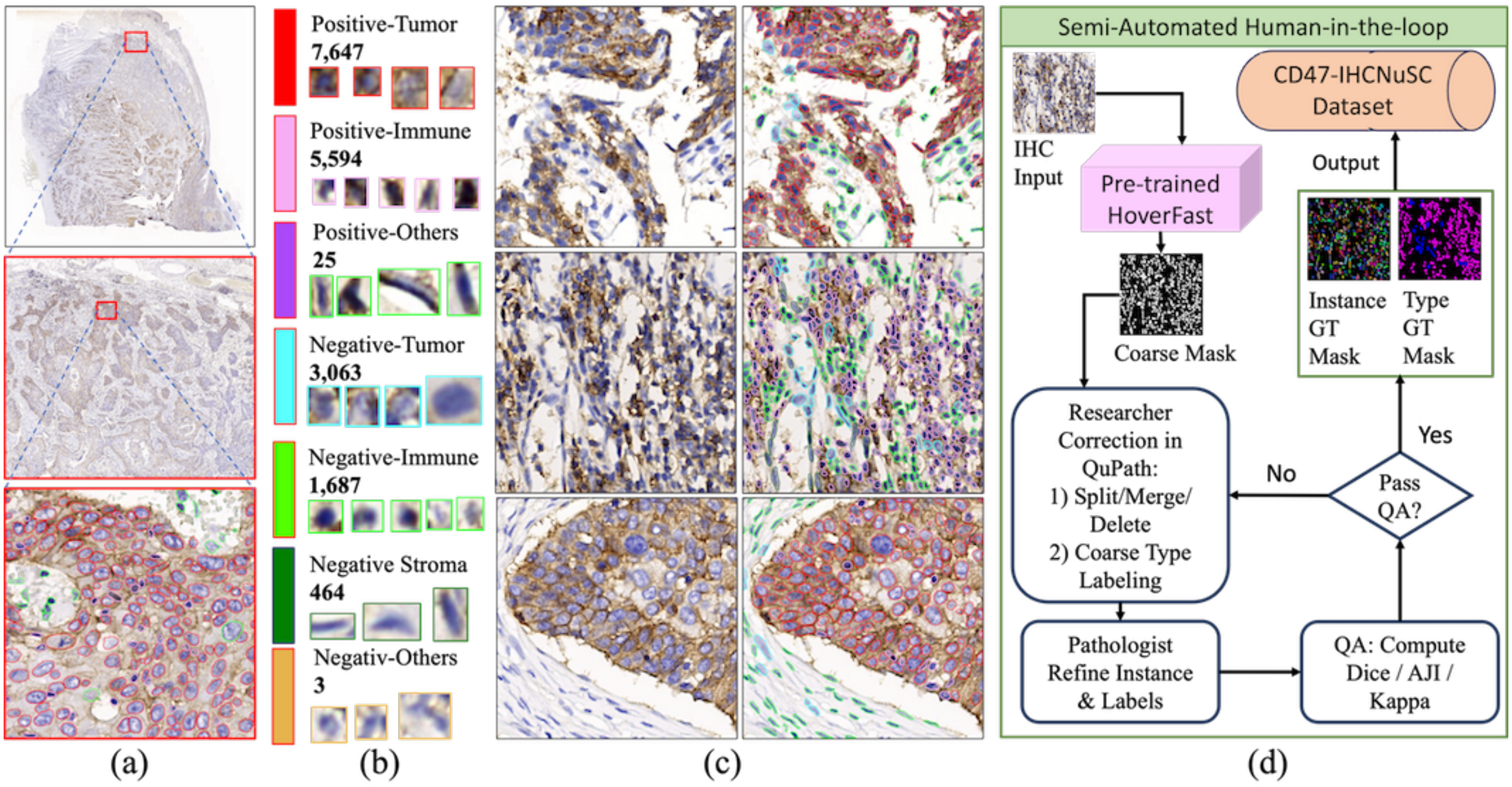

Figure 2. Overview of the CD47-IHCNuSC dataset and annotation workflow. (a) Representative CD47-stained WSIs, tumor-enriched regions, and extracted 1024×1024 patches. (b) Nuclear crops from seven annotated subtypes, including biomarker-positive and biomarker-negative tumor, immune, stromal, and rare cells; border colors correspond to subtype labels. (c) Visualization of subtype-labeled nuclei overlaid on the original image. Colors match subtype labels shown in (b). (d) Semi-automated annotation pipeline: HoverFast-based pre-labeling, manual refinement in QuPath, and expert validation with quality assurance.

Table 1. Distribution of nuclear categories and image counts in the CD47-IHCNuSC dataset. *Abbrev.*: P = Positive, N = Negative, Tu = Tumor, Im = Immune, St = Stroma, Oth = Others.

Nuclear Category	Abbrev.	Train	Test	Total	Proportion
Positive-Tumor	pTu	5,958	1,689	7,647	40.5%
Positive-Immune	pIm	4,712	882	5,594	29.6%
Positive-Others	pOth	17	8	25	0.13%
Negative-Tumor	nTu	2,631	432	3,063	16.2%
Negative-Immune	nIm	1,473	214	1,687	8.9%
Negative-Stroma	nSt	410	54	464	2.5%
Negative-Others	nOth	3	0	3	0.02%
**Total Nuclei**	–	15,204	3,279	18,483	100%
**Number of Images**	–	70	16	86	–

trained on large-scale pathology data to model contextual tissue semantics. Feature representations from both encoders are integrated within a PFAR-based global fusion neck. In addition to fusing multi-resolution encoder features, FPNuNet incorporates a multi-scale global encoder and a wavelet-based spectral extractor to enhance raw image modeling in both spatial and frequency domains. The resulting representations are decoded through three task-specific branches that jointly produce binary masks, horizontal-vertical offset vectors for instance separation, and nuclear subtype maps, enabling end-to-end learning for comprehensive NuSC.

Dual Encoder Module

Inspired by SAM2-PATH [29] and SAM2-Adapter [30], FPNuNet employs dual pre-trained encoders, i.e., Structural Encoder and Semantic Encoder, with trainable adapter layers to capture complementary features essential for robust NuSC tasks as shown in Fig. 1. Particularly, the Structural Encoder is built upon the SAM model, adept at delineating nuclear boundaries owning to its pre-trained emphasis on high-resolution structures in natural images. The Semantic Encoder uses a ViT-based UNI encoder pretrained extensively on pathology-specific datasets, enriching the model with semantic histopathological priors. During training, we freeze both encoder backbones and fine-tune only lightweight prompt adapter modules, preserving generalized pretrained knowledge and ensuring efficient parameter utilization.

Frequency-Aware Fusion Modules

To enhance both deep encoder outputs and intermediate skip connections, FPNuNet employs a series of frequency-aware fusion modules centered on the PFAR block. These modules are designed to refine multi-resolution features in the spectral domain by leveraging local context and frequency-selective representations. Specifically, a PFAR-based global fusion neck is constructed to integrate encoded features from both the SAM and UNI encoders, along with complementary information extracted from the raw input image. This fusion process is preceded by spatial and spectral enrichment of the raw image via a multi-scale module and a frequency-domain module. In parallel, PFAR blocks are also applied directly to the skip connections from the semantic encoder to strengthen shallow feature propagation. The following subsections detail the PFAR block architecture and the global fusion neck design.

PFAR Block. Although many segmentation models directly use encoder outputs and skip connection features for decoding, they often fail to preserve high-frequency structural cues essential for accurate nuclear boundary delineation particularly under stain variability and morphological ambiguity. To address this limitation, we propose a PFAR module, which integrates multi-scale spatial context, frequency-domain attention, and positional priors into a unified residual refinement framework.

The overall architecture of the PFAR module is illustrated in Fig. 3(a). Let $\mathbf{X} \in \mathbb{R}^{C\times H\times W}$ denote the input feature map, where C, H, and W represent the number of channels, height, and width, respectively. The output $\mathbf{Y}$ has the same spatial dimensions as $\mathbf{X}$. The input $\mathbf{X}$ undergoes an initial projection:

$$\mathbf{F}_0 = \phi_{\text{proj}}(\mathbf{X}),$$

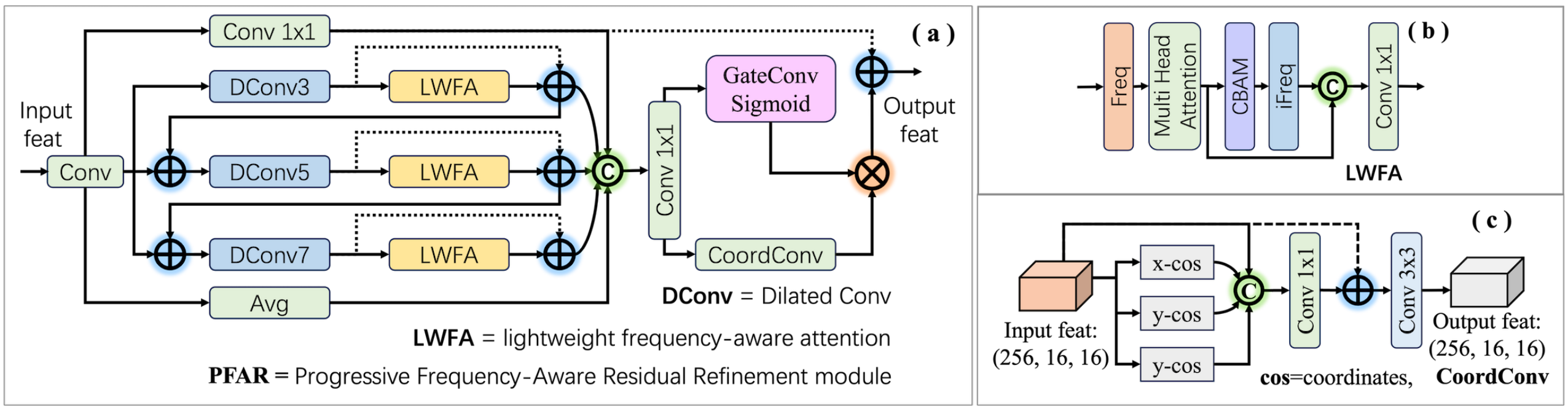

Figure 3. Illustration of the PFAR block. (a) Overview of the PFAR block. (b) Lightweight frequency-aware attention module. (c) Coordinate-aware gating.

where $\phi_{\text{proj}}(\cdot)$ denotes a 1×1 convolution used to reduce channel redundancy and produce a compact feature embedding $\mathbf{F}_0 \in \mathbb{R}^{C\times H\times W}$. This embedding anchors three progressive refinement stages. At each stage $s = 1, 2,$ and 3, a dilated convolution $\phi_{DC_k}(\cdot)$ with dilation rate $k \in \{3, 5, 7\}$ is applied to increase receptive field, followed by a lightweight frequency-aware attention module $\psi_{\text{LWFA}}(\cdot)$:

$$\mathbf{S}_s = \phi_{DC_k}(\mathbf{F}_{s-1}), \tag{1}$$

$$\mathbf{F}_s = \mathbf{S}_s + \psi_{\text{LWFA}}(\mathbf{S}_s). \tag{2}$$

here, $\phi_{DC_k}(\cdot)$ is a dilated convolution with kernel size 3×3. Each $\psi_{\text{LWFA}}(\cdot)$ first transforms the spatial feature $\mathbf{S}_s$ into the frequency domain via a real-valued discrete cosine transform (DCT), offering a compact spectral representation without the complexity of complex-valued fast Fourier transform (FFT). The DCT provides real-valued frequency representation without FFT complexity, enabling multi-head attention to focus on structure-relevant frequency components. A Convolutional Block Attention Module (CBAM) [31] further refines frequency responses through sequential channel and spatial attention. This structure enhances morphology-relevant signals while preserving computational efficiency, as shown in Fig.3(b).

To aggregate multi-scale outputs, a global context vector $\mathbf{G}$ is extracted from $\mathbf{F}_0$ via average pooling, and fused with the outputs $\{\mathbf{F}_1, \mathbf{F}_2, \mathbf{F}_3\}$ and the initial embedding $\mathbf{F}_0$ through softmax-normalized attention weights $\{\alpha_k\}$:

$$\mathbf{F} = \text{Concat}\left[\alpha_k \cdot \mathbf{O}_k\right]_{k=0}^{4}, \quad \mathbf{O} = \{\mathbf{F}_0, \mathbf{F}_1, \mathbf{F}_2, \mathbf{F}_3, \mathbf{G}\} \tag{3}$$

Finally, coordinate-aware gating injects spatial priors, as shown in Fig. 3(c):

$$\mathbf{Y} = \phi(\mathbf{F}) + \delta_{\text{coord}}(\tilde{\mathbf{F}}) \odot \sigma(\phi_{\text{gate}}(\tilde{\mathbf{F}})), \tag{4}$$

where $\tilde{\mathbf{F}}$ denotes a transformed version of $\mathbf{F}$ via a 1×1 convolution, $\phi(\cdot)$ and $\phi_{\text{gate}}(\cdot)$ are 3×3 and 1×1 convolutional layers respectively, $\delta_{\text{coord}}(\cdot)$ injects coordinate priors through trigonometric position encodings, $\sigma(\cdot)$ is the sigmoid activation function, and $\odot$ denotes the element-wise product.

PFAR-Based Global Fusion Neck. Although PFAR provides effective frequency-guided refinement for fused features, its success largely depends on the quality and semantic complementarity of encoder outputs. In multi-encoder architectures, there exists a significant challenge in harmonizing features of different spatial resolutions, semantic capacities, and domain sensitivities. Moreover, histological structures exhibit both low-frequency semantic regions (e.g., tissue context) and high-frequency cues (e.g., nuclear boundaries, biomarker deposits). To accommodate these characteristics, we design the PFAR-based global fusion neck, a multi-branch fusion module that integrates hierarchical semantic features, frequency-domain cues, and high-resolution contextual signals into a unified representation, enabling better downstream NuSC.

As illustrated in Fig. 4(a), the PFAR-based global fusion neck integrates multi-source information from three complementary inputs: 1) $\mathbf{F}_{\text{SAM}} \in \mathbb{R}^{C_s\times H\times W}$, the output of the SAM-based structural encoder; 2) $\mathbf{F}_{\text{UNI}} \in \mathbb{R}^{C_u\times H\times W}$, the output of the UNI-based semantic encoder; and 3) the multi-scale raw-image features comprising spatial descriptors $\{\mathbf{M}_{h,m,l}\}$ from a multi-scale global encoder (MSGE) as shown in Fig. 4(b) and spectral descriptors $\{\mathbf{W}_{h,m,l}\}$ from a wavelet-based frequency extractor (WVTrans) as shown in Fig. 4(c). The subscript h, m, and l indicates the high, medium, and low resolution, respectively.

To reduce computational overhead while retaining global context, the lowest-resolution spatial and frequency features, denoted as $\tilde{\mathbf{M}}_l$ and $\tilde{\mathbf{W}}_l$, are projected to match the encoder dimensions via a 1×1 convolution $\phi(\cdot)$ and concatenated with the encoder features:

$$\mathbf{F}_{\text{fusion}} = \text{Concat}\left[\phi(\mathbf{F}_{\text{SAM}}),\ \phi(\mathbf{F}_{\text{UNI}}),\ \tilde{\mathbf{M}}_l,\ \tilde{\mathbf{W}}_l\right],$$

The concatenated tensor $\mathbf{F}_{\text{fusion}} \in \mathbb{R}^{C_f\times H\times W}$ (with C_f representing the fused channel dimension) is subsequently refined by the PFAR block, yielding a spectrally enhanced representation $\mathbf{F}_{\text{pfar}}$. To further inject high-resolution detail, a lightweight gating branch modulates $\mathbf{F}_{\text{pfar}}$ using the highest-resolution spatial descriptor $\mathbf{M}_h$:

$$\mathbf{F}_{\text{hi}} = \mathbf{F}_{\text{pfar}} + \sigma(\phi_{\text{gate}}(\mathbf{M}_h)) \odot \phi(\mathbf{M}_h),$$

where $\phi_{\text{gate}}(\cdot)$ is a 1×1 convolution used to generate spatial gating weights, $\phi(\cdot)$ is a 3×3 convolution for residual enhancement, $\sigma(\cdot)$ denotes the sigmoid activation, and $\odot$ represents the element-wise product. Finally, a position-aware enhanced fusion module processes the gated feature to generate the global fusion neck output $\mathbf{F}_{\text{out}} \in \mathbb{R}^{C_o\times H\times W}$, which is forwarded to the binary, HV, and type decoder branches.

Multi-Tasks Decoders

Accurate nuclear quantification necessitates the concurrent resolution of three interrelated tasks: binary segmentation to identify nuclear regions, instance separation to delineate individual cells, and phenotype classification to assign biologically meaningful subtypes. To meet these demands, FPNuNet adopts a collaborative multi-task decoders inspired by HoverNet [7], consisting of three task-specific branches–binary, HV, and type decoders–hat operate in parallel. Beyond the HoverNet design, inter-branch feature exchange is explicitly introduced, i.e., intermediate features from the binary segmentation branch are propagated to the HV and type decoders to reinforce nuclear localization and cross-task consistency. All branches are conditioned on multi-scale, frequency-aware features from the PFAR-based fusion neck and

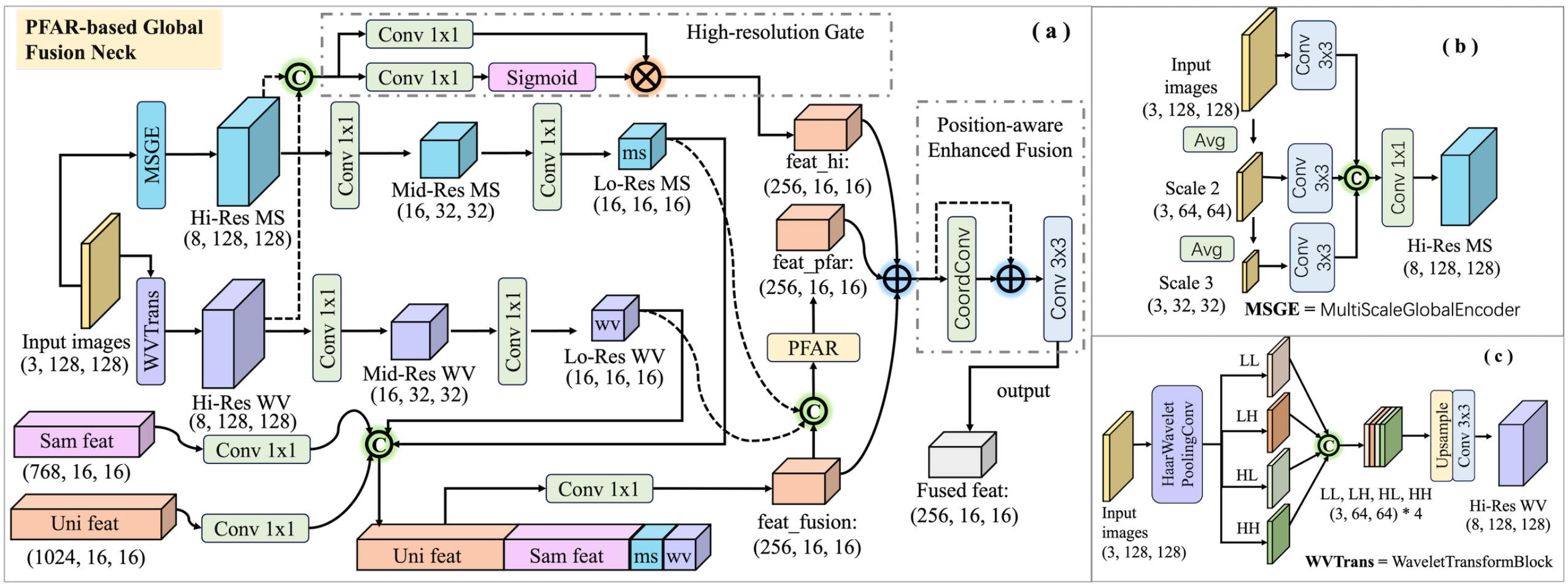

Figure 4. Illustration of the PFAR-based global fusion neck. (a) Overview of the PFAR-based global fusion neck. (b) Multi-scale global encoder. (c) Wavelet-based frequency extractor.

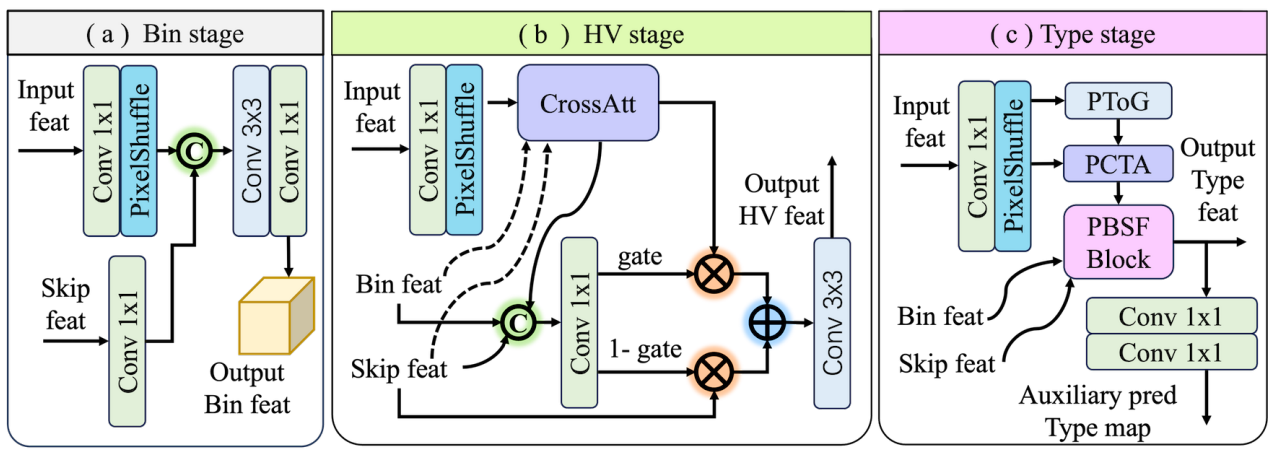

Figure 5. Collaborative multi-task decoder with three specialized branches. (a) Binary Segmentation Decoder, (b) HV Vector Regression Decoder, (c) Nuclear Type Classification Decoder (PCTA: Prompt Cross-Transformer Attention, PBSF: Prompt Binary Skip Fusion, PToG = Prompt token generators).

skip pathways, ensuring that both structural fidelity and semantic richness are preserved throughout decoding. This unified strategy supports joint optimization across tasks and enhances performance on dense and heterogeneous histopathological images.

Binary Segmentation Decoder. The binary decoder as depicted in Fig. 5(a) serves as the structural foundation for both instance separation and phenotype classification. It adopts a three-stage hierarchical design, where at each stage the fused input features are first upsampled using a 1×1 convolution followed by pixel shuffle, then concatenated with frequency-enhanced skip features from the UNI encoder. The concatenated tensor is processed by a convolutional refinement block consisting of a 3×3 convolution followed by a 1×1 projection, producing resolution-aligned feature maps for downstream tasks. The final output is a binary probability map $\hat{\mathbf{B}} \in [0,1]^{H\times W}$ indicating nuclear semantic regions.

A composite loss function combining pixel-wise and region-aware objectives is employed to supervise the training as follows:

$$\mathcal{L}_{\text{bin}} = \lambda_{(\text{bin},1)} \cdot \mathcal{L}_{\text{BCE}} + \lambda_{(\text{bin},2)} \cdot \mathcal{L}_{\text{Dice}} + \lambda_{(\text{bin},3)} \cdot \mathcal{L}_{\text{Focal}}, \quad (5)$$

where $\mathcal{L}_{\text{BCE}}$, $\mathcal{L}_{\text{Dice}}$, and $\mathcal{L}_{\text{Focal}}$ denote the binary cross-entropy (BCE), Dice, and focal loss, respectively; $\lambda_{(\text{bin},1)}$, $\lambda_{(\text{bin},2)}$, and $\lambda_{(\text{bin},3)}$ are empirically determined weighting coefficients in the binary branch. This formulation mitigates class imbalance and sharpens nuclear boundary delineation, providing reliable structural priors for the other decoding branches.

HV Vector Regression Decoder. The HV decoder as depicted in Fig. 5(b) estimates pixel-wise displacement horizontal-vectors and vertical offsets pointing from each pixel to its corresponding nuclear centroid to enable accurate instance-level separation. Let the output be $\hat{\mathbf{V}} \in \mathbb{R}^{2\times H\times W}$, where the two channels correspond to horizontal h and vertical v offsets, respectively. Each of its three decoding stages integrates three feature streams, i.e., upsampled decoder features, PFAR-enhanced skip connections, and intermediate features from the binary segmentation branch. These inputs are fused through a cross-attention mechanism, followed by a gating module that adaptively balances contextual and structural information. The aggregated features are then refined by a 3×3 convolution to produce the final HV vector map.

Supervision adopts HoverNet's regression objective:

$$\mathcal{L}_{\text{hv}} = \lambda_{(\text{hv},1)} \cdot \mathcal{L}_{\text{MSE}} + \lambda_{(\text{hv},2)} \cdot \mathcal{L}_{\text{MSGE}}, \quad (6)$$

where $\mathcal{L}_{\text{MSE}}$ is the mean squared error between predicted and ground-truth displacement vectors, and $\mathcal{L}_{\text{MSGE}}$ denotes the mean squared gradient error that promotes smooth and spatially coherent transitions across nuclear instances. $\lambda_{(\text{hv},1)}$ and $\lambda_{(\text{hv},2)}$ are weighting factors in the HV branch.

Nuclear Type Classification Decoder. The type decoder as depicted in Fig. 5(c) classifies each segmented nucleus into biologically meaningful subtypes by integrating semantic prompts and structure-aware spatial representations. Let $\hat{\mathbf{T}} \in \mathbb{R}^{K\times H\times W}$ denote the predicted type probability map across K nuclear subtypes. At each decoding stage, it receives the same tri-source input as the HV decoder: upsampled decoder features, PFAR-enhanced skip connections, and intermediate features from the binary segmentation decoder.

To introduce global semantic priors, the decoder leverages a type prompt-based encoder to generate learnable tokens per batch. We construct dedicated prompt token generators to produce learnable prompt tokens, which subsequently interact with spatial decoder features via a prompt cross-transformer attention module, infusing prompt-guided contextual cues into the decoding stream. Subsequently, the output is refined through a prompt binary skip fusion module, where skip features are aligned via prompt cross-transformer attention and modulated by a binary-guided gating mechanism that controls the contribution of structural and semantic information.

To support deep supervision, auxiliary output heads are attached to each decoding stage. The final loss aggregates outputs across stages as:

$$\mathcal{L}_{\text{type}} = \mathcal{L}_3 + 0.25(\mathcal{L}_2 + \mathcal{L}_1), \quad (7)$$

where each stage-wise loss $\mathcal{L}_i$ combines multiple classification

objectives: BCE loss ($\mathcal{L}_{\text{BCE}}$), Dice loss ($\mathcal{L}_{\text{Dice}}$), categorical cross-entropy (CE, $\mathcal{L}_{\text{CE}}$), and focal loss ($\mathcal{L}_{\text{Focal}}$), i.e.,

$$\mathcal{L}_i = \sum_j \lambda_{(\text{type},j)} \cdot \mathcal{L}_j, \quad j \in \{\text{BCE, Dice, CE, Focal}\}.$$

Here, $\lambda_{(\text{type},j)}$ are empirically selected weighting coefficients that balance pixel-wise accuracy, region-level consistency, and robustness to rare subtype predictions. This composite formulation encourages reliable classification under complex staining and class imbalance.

Overall Loss. A final objective is defined as a weighted sum of individual task losses to optimize the network jointly across all tasks:

$$\mathcal{L}_{\text{total}} = \mathcal{L}_{\text{bin}} + \mathcal{L}_{\text{hv}} + \mathcal{L}_{\text{type}} \tag{8}$$

Information flows bidirectionally across decoding branches to enhance both structural and semantic prediction. Binary segmentation maps provide spatial priors that guide the HV and type decoders via gated attention, improving instance separation and subtype boundary delineation. Conversely, semantic cues from the type decoder refine high-level binary representations during joint optimization, particularly in challenging foreground background scenarios. This collaborative mechanism promotes complementary learning, enabling FPNuNet to generate spatially aligned, structurally precise, and semantically coherent outputs.

Results

Experimental Setup

We evaluated FPNuNet on the curated CD47-IHCNuSC dataset, and processed the dataset into overlapping 124 × 124 patches (stride=64) and augmented via flips, rotations, color jittering, and stain perturbation. FPNuNet was trained with AdamW optimizer (batch size=8, weight decay=0.1), adopting learning rates of 1×10^{-3} for prompt adapters and 5×10^{-4} for other components. The learning rate was warmed up over 72 iterations and decayed at 20 and 25 epochs by factors of 0.1 and 0.01, respectively, totaling 30 epochs. The pretrained SAM (natural images) and UNI (pathology images) encoders provide initial representations, with prompt adapters fine-tuned jointly. All experiments utilize PyTorch 2.0 and MONAI on a workstation equipped with four NVIDIA RTX 4090 GPUs.

FPNuNet is compared against a broader set of related state-of-the-art methods, including **HoverNet**[7], **SRSA-Net**[13], **StarDist**[37], **HoverFast**[36], and **Cellpose-SAM** [38]. To evaluate the effects of the key components of FPNuNet, several variants are included in the ablation: **FPNuNet-F** (dual encoders SAM+UNI, an FFT-based frequency PFAE block, a concatenation-based fusion neck employing the PFAE-FFT block, and three task-specific decoders), **FPNuNet-A** (the baseline augmented with lightweight adapter layers inserted between the SAM encoder and the UNI encoder to improve cross-domain feature alignment), and **FPNuNet-AP** (the adapter variant further augmented with a type prompt-based decoder that injects learnable subtype tokens into the type branch). For a controlled comparison, the FPNuNet series, HoverNet, and SRSA-Net were trained on the same dataset using identical training splits and protocols. By contrast, StarDist, HoverFast, and Cellpose-SAM are instance-segmentation–only baselines. They were evaluated using the official publicly released pretrained weights without fine-tuning on CD47-IHCNuSC dataset and therefore participated in the semantic/instance segmentation comparisons but not in subtype classification.

Evaluation and Metrics

The performance of nuclei analysis models is assessed along two complementary axes: 1) nuclear instance segmentation, which evaluates the recovery of object identities and boundaries; and 2) nuclei classification, which covers both dense, pixel-wise semantic segmentation (treated as per-pixel classification) and instance-level subtype recognition.

Nuclear Instance Segmentation Evaluation

Nuclei instance segmentation is quantified using complementary criteria that assess how well a method detects, separates, and delineates individual instances. Given a set of ground-truth (GT) nuclei mask $\mathcal{G}$ and a set of predicted nuclei mask $\mathcal{S}$ in an image. Region-overlap metrics such as the Intersection-over-Union (IoU) and Dice coefficient (**Dice**) [39] measure pixel-wise agreement between the corresponding regions:

$$\text{IoU} = \frac{|G \cap S|}{|G \cup S|}, \tag{9}$$

$$\text{Dice} = \frac{2\,|G \cap S|}{|G| + |S|}. \tag{10}$$

The pairwise aggregate variant (**Dice2**) [40] computes Dice over all matched ground-truth–prediction pairs and averages them, providing a more instance-sensitive estimate of segmentation accuracy.

To further incorporate unmatched regions, the Aggregated Jaccard Index (AJI) [41] aggregates intersections and unions over all matched pairs, penalizing unpaired ground-truth or prediction regions:

$$\text{AJI} = \frac{\sum |G \cap S|}{\sum |G \cup S| + \sum |G_{\text{unmatched}}| + \sum |S_{\text{unmatched}}|}. \tag{11}$$

Its assignment-robust variant AJI+ replaces greedy matching with optimal bipartite (Hungarian) assignment to better handle clumped or overlapping nuclei [7].

Following the HoVer-Net [7] and SRSN-Net evaluation protocol [13], Panoptic Quality (PQ) [42] is reported together with its decomposition into Detection Quality (DQ) and Segmentation Quality (SQ). Let TP (true positives), TN (true negatives), FP (false positives), and FN (false negatives) represent, respectively, the correctly detected nuclei, unmatched predictions, and missed GT nuclei. Then DQ and SQ are defined as:

$$\text{DQ} = \frac{\text{TP}}{\text{TP} + 0.5\,\text{FP} + 0.5\,\text{FN}}, \tag{12}$$

$$\text{SQ} = \frac{1}{\text{TP}} \sum \text{IoU}(G, S), \tag{13}$$

and the overall PQ is obtained as:

$$\text{PQ} = \text{DQ} \times \text{SQ}. \tag{14}$$

In addition, we evaluate model performance using Precision (**P**), Recall (**R**), and F1-score ($\mathbf{F}_1$), which are mathematically defined as:

$$\mathbf{P} = \frac{\text{TP}}{\text{TP} + \text{FP}}, \quad \mathbf{R} = \frac{\text{TP}}{\text{TP} + \text{FN}}, \quad \mathbf{F}_1 = \frac{2\,\mathbf{P}\,\mathbf{R}}{\mathbf{P} + \mathbf{R}}. \tag{15}$$

Here, to differentiate the instance-level metrics, we define the object-level Precision ($\mathbf{P}_{\text{obj}}$), Recall ($\mathbf{R}_{\text{obj}}$), and F1-score ($\mathbf{F}_{1,\text{obj}}$) using the nuclei-level TP, FP, FN, and TN. Taken together, these metrics furnish a holistic view spanning pixel-wise overlap, in-

Table 2. Quantitative Evaluation of Instance Segmentation on the CD47-IHCNuSC Test Set.

Model	Dice1	Dice2	AJI	AJI+	bPQ	DQ	SQ	$F_{1,inst}$	P_{inst}	R_{inst}
Hovernet	**0.9264**	0.7541	0.6962	0.7307	0.7303	0.8387	**0.8700**	0.8438	0.8647	0.8264
SRSA-Net	0.9051	0.6640	0.5101	0.5822	0.5872	0.7076	0.8292	0.7189	0.7964	0.6588
HoverFast	0.7403	0.6944	0.5144	0.5256	0.5057	0.6483	0.7785	0.6524	0.7395	0.5903
StarDist	0.7019	0.5751	0.4503	0.4697	0.3529	0.4737	0.7402	0.4753	0.3784	0.7114
Cellpose	0.8585	**0.7766**	0.7101	0.7263	0.6970	0.8542	0.8147	0.8594	**0.9130**	0.8147
FPNuNet-F	0.9017	0.6917	0.7048	0.7256	0.6814	0.8221	0.8286	0.8266	0.7882	0.8723
FPNuNet-A	0.9055	0.6992	0.7298	0.7443	0.7018	0.8443	0.8310	0.8490	0.8109	**0.8921**
FPNuNet-AP	0.9100	0.7118	0.7303	0.7502	0.7114	0.8431	0.8436	0.8477	0.8122	0.8880
FPNuNet	0.9215	0.7296	**0.7472**	**0.7717**	**0.7458**	**0.8589**	0.8677	**0.8637**	0.8462	0.8834

stance separation, boundary accuracy, and assignment robustness.

Nuclear Classification Evaluation

Nuclear type classification is evaluated using instance-level metrics that assess the correctness of subtype labels assigned to individual nuclei, taking into account the detection performance. Given the GT set with type annotations $\mathcal{G} = \{(g_i, y_i)\}_{i=1}^{N}$ and the predicted set with assigned types $\mathcal{S} = \{(s_j, \hat{y}_j)\}_{j=1}^{M}$, a one-to-one correspondence $\mathcal{M} \subseteq \mathcal{G} \times \mathcal{S}$ is established by matching predicted and GT nuclei using the *argmax* assignment strategy, which selects the predicted label with the highest probability for each ground-truth nucleus. Background (class 0) is excluded.

For each matched pair (g_i, s_j), a TP is counted if $\hat{y}_j = y_i$. A label mismatch contributes a FP for the predicted class $\hat{y}_j$ and a FN for the GT class y_i. Unmatched predictions and unmatched GT instances are counted as FP and FN, respectively. This matching procedure yields the per-class counts $\{TP^{(k)}, FP^{(k)}, FN^{(k)}\}_{k=1}^{K}$, which form the basis for the macro-, micro-, and support-weighted F_1 scores, as well as accuracy variants outlined below.

For each class $k \in \{\text{pTu}, \text{pIm}, \text{nTu}, \text{nIm}\}$ (excluding background), the instance-level Precision ($\mathrm{P}_{\text{inst}}^{(k)}$), Recall ($\mathrm{R}_{\text{inst}}^{(k)}$), and F1-score ($\mathrm{F}_{1,\text{inst}}^{(k)}$) are calculated as follows:

$$\mathrm{P}_{\text{inst}}^{(k)} = \frac{TP^{(k)}}{TP^{(k)} + FP^{(k)}}, \quad \mathrm{R}_{\text{inst}}^{(k)} = \frac{TP^{(k)}}{TP^{(k)} + FN^{(k)}}, \tag{16}$$

$$\mathrm{F}_{1,\text{inst}}^{(k)} = \frac{2\,\mathrm{P}_{\text{inst}}^{(k)}\,\mathrm{R}_{\text{inst}}^{(k)}}{\mathrm{P}_{\text{inst}}^{(k)} + \mathrm{R}_{\text{inst}}^{(k)}}. \tag{17}$$

The macro-averaged scores $\mathbf{P}_{\text{cls, macro}}$ and $\mathbf{F}_{1,\text{macro}}$ are obtained by averaging the per-class instance-level precision and F_1 with equal weights. The support-weighted metrics $\mathbf{P}_{\mathrm{W}}$ and $\mathbf{F}_{1,\mathrm{W}}$ weight per-class scores by class support, with $w_k = n^{(k)}/N$ where $n^{(k)}$ is the number of GT nuclei in class k and $N = \sum_k n^{(k)}$. The micro-averaged F_1 score $\mathbf{F}_{1,\text{micro}}$ is computed by aggregating the TP, FP, and FN across all classes and then calculating the F_1 score based on the total counts.

For further evaluation, the overall metrics include mPQ, which is the mean Panoptic Quality over non-background classes. mPQ is computed as $\text{mPQ} = \frac{1}{K}\sum_{k=1}^{K} \text{PQ}^{(k)}$, where $\text{PQ}^{(k)} = \text{DQ}^{(k)} \times \text{SQ}^{(k)}$ as defined in the instance segmentation subsection. A match is counted as $TP^{(k)}$ only when the predicted type equals the ground-truth type k (type-consistent PQ).

Nuclear Instance Segmentation Performance

Table 2, Fig. 6 and Fig. 7 report the instance-level performance of the compared methods on CD47-IHCNuSC in terms of DQ, SQ, AJI/AJI+ and bPQ. FPNuNet attains the best overall results, with AJI = 0.8133, AJI+ = 0.7331, bPQ = 0.7595, and the highest $\mathbf{F}_{1,\text{inst}}$/DQ = 0.8751 alongside competitive SQ = 0.8673. The box plots in Fig. 7 show higher medians and tighter interquartile ranges for AJI, AJI+, and bPQ, indicating stable gains across individual test images rather than improvements confined to a small subset. Consistent qualitative patterns are visible in Fig. 6, where FPNuNet produces cleaner splits in crowded clusters, smoother yet well-aligned contours along membrane staining, and fewer missed small nuclei than competing methods.

Ablation over the FPNuNet family demonstrates progressive and complementary contributions. From FPNuNet-F to the adapter variant FPNuNet-A, DQ increases from 0.8221 to 0.8443 (+2.70%), with concurrent gains in AJI (+3.55%), AJI^{+} (+2.58%), and bPQ (+2.99%), indicating that adapters primarily improve instance recovery and contact handling under weak IHC signal. Introducing the prompt-guided decoder (FPNuNet-AP) further strengthens separation and contour reliability over FPNuNet-A: bPQ +1.37%, AJI+ +0.79%, and SQ +1.52%, while DQ is essentially maintained (−0.14%). Integrating the complete fusion design (FPNuNet) yields additional gains over FPNuNet-AP: bPQ +4.83%, AJI +2.31%, AJI+ +2.86%, DQ/$\mathbf{F}_{1,\text{inst}}$ +1.87%/ + 1.89%, and SQ +2.86%, accompanied by higher $\mathbf{P}_{\text{inst}}$ (+4.19%) with a negligible change in $\mathbf{R}_{\text{inst}}$ (−0.52%). These effects are evidenced by upward shifts in the medians and reduced interquartile ranges in Fig. 7, and by the qualitative overlays in Fig. 6, where adapters reduce missed detections at low-contrast boundaries, the prompt-guided decoder suppresses residual merges in crowded regions, and the final fusion stabilizes boundaries.

Compared with strong external baselines trained under the same protocol, FPNuNet achieves superior instance segmentation on CD47-IHCNuSC. Relative to HoverNet, region–overlap and boundary metrics improve (Table 2): AJI = 0.7472 vs. 0.6962 (+7.34%), AJI+ = 0.7717 vs. 0.7307 (+5.61%), and bPQ = 0.7458 vs. 0.7303 (+2.12%), with essentially unchanged shape fidelity (SQ = 0.8677 vs. 0.8700, −0.26%) and higher detection quality ($\mathbf{F}_{1,\text{inst}}$/DQ = 0.8637/0.8589 vs. 0.8438/0.8387, +2.36%/ + 2.41%). These gains are recall-driven, with $\mathbf{R}_{\text{inst}}$ = 0.8834 vs. 0.8264 (+6.90%) at slightly lower $\mathbf{P}_{\text{inst}}$ = 0.8462 vs. 0.8647 (−2.14%), indicating more complete recovery of difficult and touching nuclei without compromising contour fidelity; correspondingly, the AJI/AJI+ box plots in Fig. 7 show upward shifts in medians and tighter interquartile ranges, and the qualitative overlays in Fig. 6 reveal fewer small-object misses and cleaner cluster splits. Against SRSA-Net, margins are larger across all axes—AJI (+46.49%), AJI+ (+32.55%), bPQ (+27.01%), and DQ (+21.39%)—consistent with SRSA-Net's lower $\mathbf{R}_{\text{inst}}$ = 0.6588 and broader dispersion in Fig. 7; Fig. 6 further shows residual merges and contour bleeding under dense packing for SRSA-Net, whereas FPNuNet maintains well-delineated boundaries. Collectively, the evidence points to a recall- and separation-oriented operating point with calibrated boundaries, consistent with the intended effect

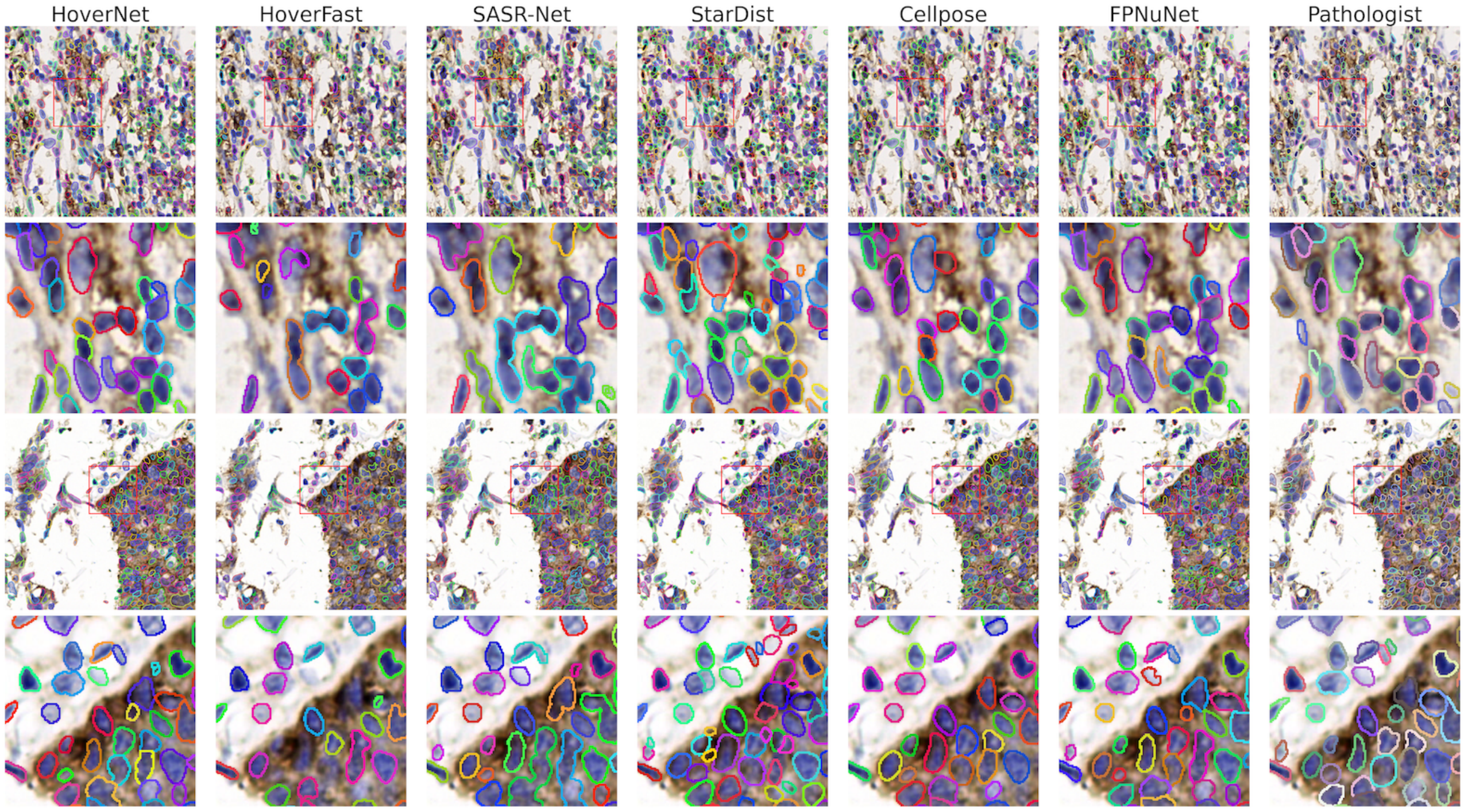

Figure 6. Qualitative comparisons of instance segmentation models on representative CD47-IHCNuSC test samples. Colors are used solely to distinguish individual nuclei. HoVerFast, Cellpose and StarDist are incorporated for comparison, both evaluated using their pretrained weights.

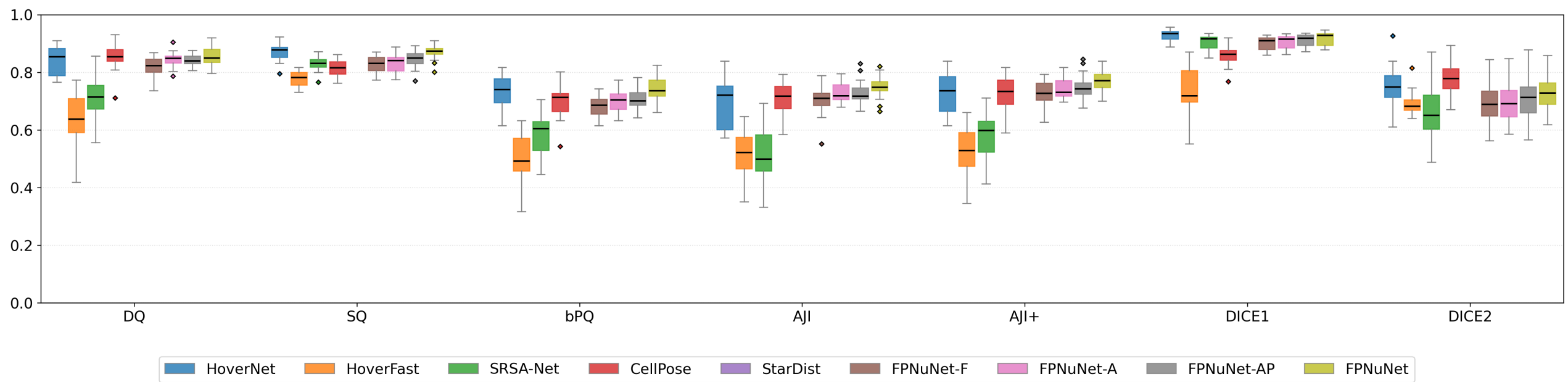

Figure 7. Box plots highlighting the performance of competing instance segmentation methods on the CD47-IHCNuSC dataset. Each box summarizes the distribution of evaluation metrics (*DQ*, *SQ*, *bPQ*, *AJI*, *AJI+*, *DICE1*, and *DICE2*) across all test images.

of frequency-aware refinement and prompt-guided decoding for IHC data.

Relative to instance-only baselines evaluated with public pretrained weights, FPNuNet exceeds Cellpose on overlap and boundary measures—AJI (+5.23%), AJI+ (+6.25%), bPQ (+7.00%), SQ (+6.51%), and DQ (+0.55%)—while trading lower $\mathbf{P}_{\text{inst}}$ (−7.32%) for higher $\mathbf{R}_{\text{inst}}$ (+8.43%), indicating a more recall- and separation-oriented operating point with better boundary fidelity in IHC settings. Improvements over HoverFast and StarDist are more pronounced, reflecting the sensitivity of these designs to IHC appearance shifts and irregular nuclear morphology. Overall, the numerical margins, tighter dispersion in Fig. 7, and the overlays in Fig. 6 jointly indicate that FPNuNet achieves a more balanced trade-off among detection (DQ/$\mathbf{R}_{\text{inst}}$), separation (AJI+), and contour fidelity (bPQ/SQ) on CD47-IHCNuSC.

Nuclear Type Classification Performance

Table 3 and Fig. 8 summarize the subtype classification performance of the compared methods on CD47-IHCNuSC. FPNuNet delivers the strongest aggregate performance across type-aware panoptic quality (*mPQ*) and class-averaged metrics, attaining mPQ = 0.4838 (HoverNet: 0.4730, SRSA-Net: 0.3617), $\mathbf{P}_{\text{macro}}$ = 0.7097 (vs. 0.6966/0.7029), $\mathbf{F}_{1,\text{macro}}$ = 0.6481 (vs. 0.6349/0.5779), $\mathbf{F}_{1,\text{micro}}$ = 0.8239 (vs. 0.7984/0.7410), and $\mathbf{F}_{1,w}$ = 0.8317 (vs. 0.8172/0.7668). HoverNet retains the highest prevalence-weighted precision $\mathbf{P}_w$ (0.8761; FPNuNet: 0.8586), but FPNuNet's higher macro-averaged precision and F_1 indicate better across-class balance rather than reliance on majority categories. Qualitative overlays in Fig. 8 show fewer subtype flips at tumor–stroma interfaces and more coherent immune predictions under FPNuNet, supporting these aggregate trends.

The ablation study within the FPNuNet family systematically clarifies the contribution of each module on type recognition. Starting from FPNuNet-F, the adapter-only variant (FPNuNet-A) reduces detection-aware type quality and macro-level classification (mPQ: 0.3334 vs. 0.3488, −4.4%; $\mathbf{F}_{1,\text{macro}}$: 0.4653 vs. 0.5028), indicating that cross-domain alignment alone is insufficient for phenotype separability. Adding the prompt-guided type decoder (FPNuNet-AP) substantially improves discrimination over FPNuNet-A, lifting $\mathbf{P}_{\text{macro}}$ to 0.6888 (+24.5%), $\mathbf{F}_{1,\text{macro}}$ to 0.5879 (+26.4%), $\mathbf{F}_{1,w}$ to 0.8000 (+6.5%), and mPQ to 0.4129 (+23.9%). Integrating the complete fusion design (FPNuNet) yields further gains over FPNuNet-AP: $\mathbf{F}_{1,\text{micro}}$ = 0.8239 (+4.2%), $\mathbf{P}_{\text{macro}}$ = 0.7097 (+3.0%), $\mathbf{F}_{1,\text{macro}}$ = 0.6481 (+10.3%), $\mathbf{F}_{1,w}$ = 0.8317 (+4.0%), and mPQ = 0.4838 (+17.2%). These step-wise

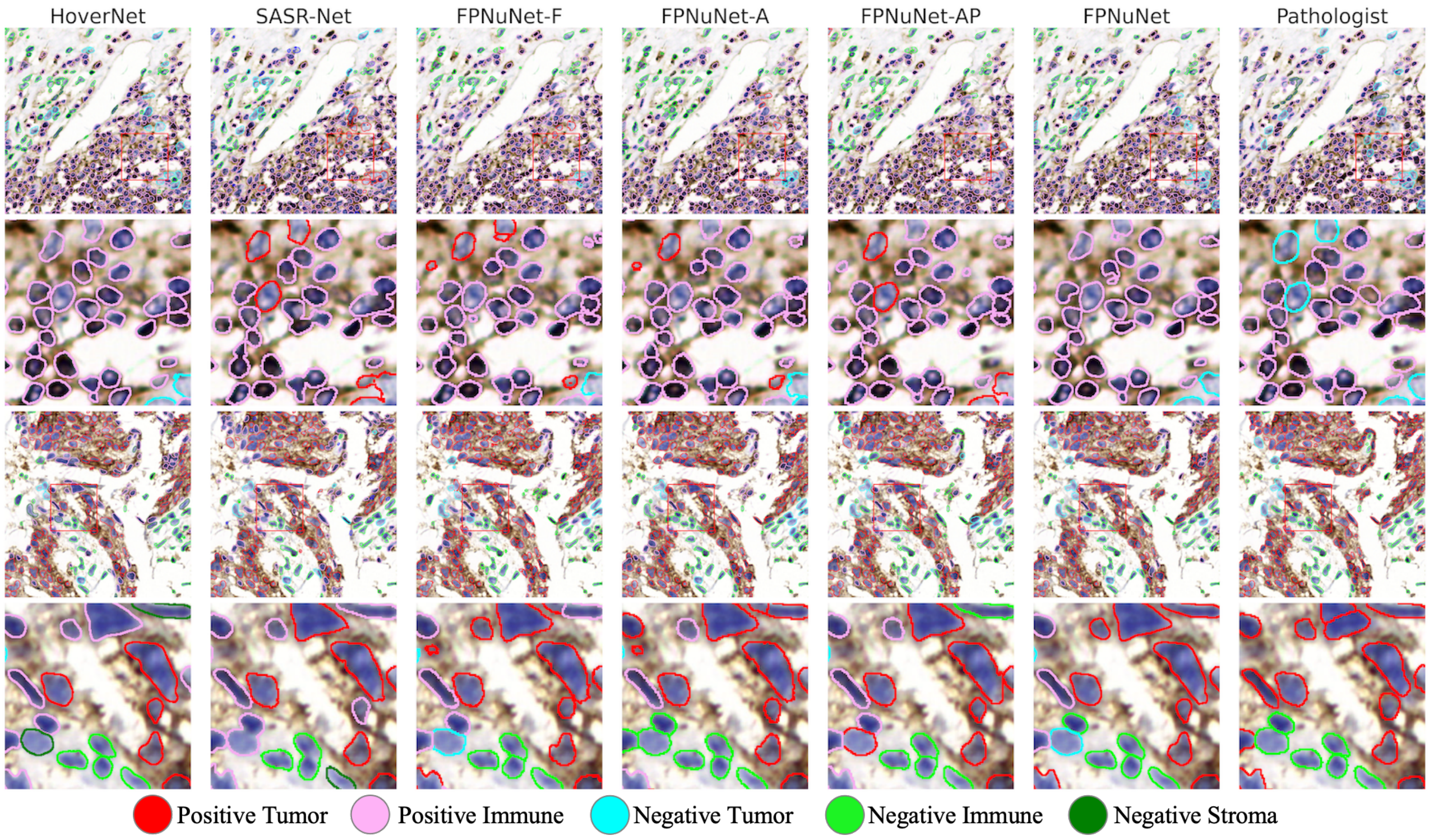

Figure 8. Qualitative comparisons of NuSC-capable models on representative CD47-IHCNuSC test samples. All models output instance masks, and subtype classifications are color-coded for models supporting classification according to the defined color mapping.

Table 3. Quantitative Evaluation of Classification on the CD47-IHCNuSC Test Set.

Metric	HoverNet	SRSA-Net	FPNuNet-F	FPNuNet-A	FPNuNet-AP	FPNuNet
**Overall**						
mPQ	0.4730	0.3617	0.3488	0.3334	0.4129	**0.4838**
P_{macro}	0.6966	0.7029	0.5985	0.5534	0.6888	**0.7097**
P_{w}	**0.8761**	0.8648	0.7929	0.7788	0.8458	0.8586
$F_{1,cls}$	0.6349	0.5779	0.5028	0.4653	0.5879	**0.6481**
$F_{1,micro}$	0.7984	0.7410	0.7767	0.7647	0.7906	**0.8239**
$F_{1,w}$	0.8172	0.7668	0.7634	0.7513	0.8000	**0.8317**
**Per-class: Positive Tumor (pTu)**						
PQ_{inst}^{pTu}	0.6649	0.5207	0.6157	0.6297	0.6691	**0.6894**
P_{inst}^{pTu}	**0.9496**	0.8984	0.8724	0.8712	0.9098	0.9239
R_{inst}^{pTu}	0.8577	0.8395	**0.9353**	0.9171	0.8972	0.9308
$F_{1,inst}^{pTu}$	0.9013	0.8679	0.9028	0.8935	0.9035	**0.9273**
**Per-class: Positive Immune (pIm)**						
PQ_{inst}^{pIm}	**0.5564**	0.4124	0.2760	0.2236	0.3657	0.5103
P_{inst}^{pIm}	0.7324	0.6640	0.7344	0.7439	0.7591	**0.8268**
R_{inst}^{pIm}	**0.8534**	0.8130	0.7526	0.7066	0.7931	0.8344
$F_{1,inst}^{pIm}$	0.7883	0.7310	0.7434	0.7248	0.7758	**0.8306**
**Per-class: Negative Tumor (nTu)**						
PQ_{inst}^{nTu}	0.3046	0.2385	0.2291	0.2313	0.3334	**0.3823**
P_{inst}^{nTu}	0.5575	0.5766	0.5753	0.5105	0.6108	**0.6623**
R_{inst}^{nTu}	**0.5808**	0.4812	0.5153	0.5243	0.5667	0.5397
$F_{1,inst}^{nTu}$	0.5689	0.5246	0.5437	0.5173	0.5879	**0.5948**
**Per-class: Negative Immune (nIm)**						
PQ_{inst}^{nIm}	0.3945	0.2927	0.3448	0.3234	0.3776	**0.4476**
P_{inst}^{nIm}	**0.6547**	0.6314	0.6409	0.5430	0.5649	0.6093
R_{inst}^{nIm}	0.6282	0.5533	0.5505	0.5459	0.6458	**0.7225**
$F_{1,inst}^{nIm}$	0.6412	0.5897	0.5923	0.5445	0.6027	**0.6611**

gains align with the class-wise trends in Table 3: the prompt decoder curbs cross-class confusion in crowded fields (e.g., higher $\mathbf{F}_{1,\text{inst}}^{pIm}$), while the final fusion stabilizes decisions under stain heterogeneity (e.g., higher $\text{PQ}_{\text{inst}}^{nIm}$). Consistent with these quantitative trends, Fig. 8 shows fewer subtype flips at tumor–stroma interfaces and more coherent immune predictions under FPNuNet; adapters reduce missed immune instances at lowcontrast membranes, the prompt decoder suppresses crossclass confusion in crowded fields, and the final fusion stabilizes decisions under heterogeneous staining.

Under identical protocols, FPNuNet surpasses HoverNet and SRSA-Net on mPQ and macro/micro-averaged metrics (Table 3): mPQ = 0.4838 (vs. 0.4730/0.3617), $\mathbf{P}_{\text{macro}}$ = 0.7097 (vs. 0.6966/0.7029), $\mathbf{F}_{1,\text{macro}}$ = 0.6481 (vs. 0.6349/0.5779), $\mathbf{F}_{1,\text{micro}}$ = 0.8239 (vs. 0.7984/0.7410), and $\mathbf{F}_{1,w}$ = 0.8317 (vs. 0.8172/0.7668). Although HoverNet retains a marginal advantage in $\mathbf{P}_w$ (0.8761 vs. 0.8586 for FPNuNet), but FPNuNet's higher **P**macro and $\mathbf{F}_{1,\text{macro}}$ indicate better acrossclass balance rather than reliance on majority categories; qualitative overlays in Fig. 8 show fewer subtype flips at tumor–stroma interfaces and more coherent immune delineations under FPNuNet.

FPNuNet exhibits the strongest type-aware aggregate performance (higher mPQ, $\mathbf{P}_{\text{macro}}$, $\mathbf{F}_{1,\text{macro}}$, $\mathbf{F}_{1,\text{micro}}$, and $\mathbf{F}_{1,w}$; Table 3), while HoverNet retains a small edge in prevalence-weighted precision $\mathbf{P}_w$. At the class level, FPNuNet attains the highest $\text{PQ}_{\text{inst}}^{pTu}$ and $\text{F}_{1,\text{inst}}^{pTu}$ with strong recall, whereas HoverNet yields the highest $\text{P}_{\text{inst}}^{pTu}$, indicating a recall–precision trade-off on tumor nuclei. For positive immune (pIm), FPNuNet improves precision and F_1 but falls slightly short of HoverNet in recall and type-consistent PQ_{inst}, suggesting residual detection/assignment sensitivity in crowded lymphocyte clusters. For negative tumor (nTu), FPNuNet raises precision and F_1 with recall comparable to HoverNet, leading to higher PQ_{inst} and fewer false positives at tumor–non-tumor interfaces. For negative immune (nIm), FPNuNet markedly improves recall with a minor precision trade-off, yielding superior F_1 and PQ_{inst} and reflecting better recovery of diffuse stromal immune cells. Taken together, gains concentrate in minority and morphologically challenging subtypes (nTu, nIm) while maintaining strong pTu performance—explaining the improvements in macro/micro averages and mPQ—whereas HoverNet's advantage in $\mathbf{P}_w$ is consistent with weighting toward the prevalent tumor class. Remaining gaps, most notably pIm PQ_{inst}, highlight headroom in small-object detection and assignment (e.g., class-specific matching thresholds or small-nucleus priors) to convert improved typing into higher panoptic quality.

Finally, cross-task consistency with the instance results helps explain the classification gains. The higher-quality instance masks and improved separation reported for FPNuNet reduce label noise at interfaces, producing cleaner per-object features that benefit subtype discrimination. In particular, better boundary localisation lessens tumor–stroma contamination and improves immune typing in densely packed regions, as qualitatively illustrated in Fig. 8.

In summary, FPNuNet achieves consistent and significant improvements across all evaluated tasks by integrating DCT-based frequency-aware refinement, adapter-guided dual encoding, and graph-based subtype decoding. Through systematic ablation, we demonstrate the complementary effect of these modules, particularly in enhancing subtype classification under challenging IHC conditions. These findings establish FPNuNet as a robust and generalizable framework for clinically meaningful applications such as tumor cellularity estimation, immune phenotyping, and biomarker-driven pathology analysis.

Discussion

This work makes dual contributions to biomarker-aware nuclei analysis in IHC-stained esophageal carcinoma: 1) **CD47-IHCNuSC**, to our knowledge the first expert-verified dataset for CD47-stained esophageal carcinoma with seven clinically meaningful nuclear subtypes; and 2) a spectrally informed, prompt-guided architecture (**FPNuNet**) that unifies structure-sensitive features from a SAM backbone with pathology-specific semantics from a ViT encoder using lightweight prompt adapters and a progressive DCT-based refinement neck. Together, these advances deliver consistent improvements in instance separation, boundary calibration, and subtype recognition on IHC images, with notable gains in panoptic quality and in the recognition of rare subtypes.

CD47-IHCNuSC addresses a gap in IHC resources by providing a biomarker-specific, expert-validated benchmark with fine-grained subtype labels. By deliberately preserving real-world staining heterogeneity and exhibiting a long-tailed class distribution, the dataset enables evaluation under IHC-specific failure modes (e.g., pigment or counterstain variation and weak or uneven chromogen) and stresses minority-class recognition. These properties help contextualize the observed advantages of FPNuNet, suggesting that methods which explicitly accommodate stain variability and class imbalance are better positioned to generalize. They also motivate designs that inject tissue-region priors into nuclei pipelines—either as postprocessing constraints or through sequential/joint tissue–nuclei modeling—to further improve subtype fidelity.

The effectiveness of FPNuNet appears to stem from a complementary interplay between frequency-space refinement and dual-encoder fusion. The progressive DCT-based refinement neck emphasizes high-frequency morphological cues, aiding nuclear boundary preservation and instance separation under staining variability. In parallel, the dual-encoder design unifies structure-sensitive features from a SAM backbone with pathology-specific semantics from a ViT encoder, while lightweight prompt adapters align feature spaces without extensive fine-tuning of large backbones. Ablation analyses indicate that spectral refinement and prompt-based alignment contribute additively, and qualitative audits reveal fewer over-merging errors in densely packed tumor regions and reduced confusions between morphologically proximate subtypes (e.g., immune-related vs. tumor-associated nuclei), —consistent with improved boundary calibration and subtype cue modeling.

Despite the promising results, this study still has several limitations. First, the dataset is restricted to a single biomarker (CD47) and tumor site, which limits assessment of inter-institutional variability and cross-antibody generalizability. Second, dense instance-level annotation can introduce residual label noise and inter-observer variability—particularly for rare or overlapping morphologies (e.g., immune-related vs. stromal)—potentially capping performance on minority classes. Third, the dual-encoder design increases memory footprint and latency relative to single-backbone baselines, constraining deployment on resource-limited systems. Finally, although the baseline set is representative, prospective studies and external validation on held-out cohorts are needed to more fully characterize generalization.

Potential future work could extend CD47-IHCNuSC to additional IHC biomarkers and institutions and investigate semi- and self-supervised domain adaptation to mitigate scanner- and stain-induced shifts. Methodologically, emphasis can be placed on adapter-based integration with pathology foundation models while keeping large encoders largely frozen; on hybrid DCT/FFT modules and learnable spectral filters to enhance multi-scale detail preservation; and on explicit incorporation of tissue-region priors through sequential or joint tissue–nuclei architectures. In parallel, uncertainty estimation and active learning can be explored to prioritize expert review where predictions are am-

biguous. At the application level, tile- and cell-level outputs can be linked to slide-level endpoints—including tumor purity estimation, immune contexture quantification, and biomarker-driven risk stratification—to more rigorously assess clinical utility within realistic diagnostic workflows.

Together, the biomarker-specific CD47-IHCNuSC benchmark and the spectrally informed multi-task FPNuNet provide a foundation for reproducible, IHC-aware nuclei analysis and facilitate integration into digital pathology workflows. In the near term, improved instance separation and subtype recognition can enable pre-analytical triage for molecular assays (e.g., purity thresholds), quantitative assessment of the tumor–immune microenvironment, and scalable quality control. In the longer term, coupling histology-derived phenotypes with spatial omics could clarify how cellular identity shapes morphology and, ultimately, patient outcomes.

Availability of Source Code and Requirements

Project name: FPNuNet
Project home page: https://github.com/LuluQin766/FPNuNet
Operating system(s): Linux
Programming language: Python (≥3.7)
Other requirements: PyTorch (≥1.13.0), torchvision (≥0.14.0), PyTorch Lightning, NumPy (≥1.20), SciPy (≥1.10), Pillow (≥9.4), OpenCV (≥4.7), scikit-learn (≥1.2), PyYAML (≥6.0), timm (≥0.3.2), matplotlib (≥3.1), tqdm, tensorboardX, onnxruntime (≥1.14), imageio (≥2.9), h5py
License: MIT License
PFNuNet is registered as a software application on on SciCrunch (RRID: SCR_027659) and biotools (biotools:fpnunet)

Data Availability

The CD47-IHCNuSC dataset, including all image patches, instance-level annotations, type maps, and associated metadata, has been deposited in Zenodo under an embargoed/private access setting. To allow peer review, a private access link has been provided to the editors and reviewers (access link: private Zenodo link). The dataset will be publicly released upon acceptance of the manuscript.

Abbreviations

AJI: aggregated Jaccard index; BCE: binary cross-entropy; CBAM: convolutional block attention module; CD47: cluster of differentiation 47; Cls.: classification; CNN: convolutional neural network; DAB: diaminobenzidine; DQ: detection quality; FPNuNet: frequency-aware prompt-guided network; H&E: hematoxylin and eosin; HV: horizontal–vertical; IHC: immunohistochemistry; Inst. Seg.: instance segmentation; Mag.: magnification; MSE: mean squared error; MSGE: multi-scale global encoder; NuSC: nuclear segmentation and classification; PFAR: progressive frequency-aware residual; PQ: panoptic quality; ROI: region of interest; SAM: segment anything model; Seg.: segmentation; SQ: segmentation quality; UNI: ViT-based universal pathology encoder; ViT: vision transformer; WSI: whole-slide image.

Ethics Approval and Consent to Participate

The present study has been approved by the Ethics Committee of Jiangjin Central Hospital, Chongqing Municipality (KY2022037) and complies with all relevant ethical, legal, and institutional regulations.

Competing interests

No competing interest is declared.

Funding

This work was supported in part by the National Key Research and Development Program of China, under Grant 2022YFF1202104, and in part by the National Natural Science Foundation of China under Grant 62471310.

Author contributions statement

L.Q., Zh.P., X.H., J.Zh., and Z.Z. conceived the experiments and analyzed the results. L.Q. J.Zh., and Z.Z. conducted the experiments. L.Q. and Zh.P. annotated the dataset. L.Q. and Z.Z. wrote and reviewed the manuscript. All authors approved the final version.

References

1. McGenity C, Clarke EL, Jennings C, Matthews G, Cartlidge C, Freduah-Agyemang H, et al. Artificial Intelligence in Digital Pathology: A Systematic Review and Meta-Analysis of Diagnostic Test Accuracy. npj Digital Medicine 2024;7(1):1–19.
2. Hosseini MS, Bejnordi BE, Trinh VQH, Chan L, Hasan D, Li X, et al. Computational Pathology: A Survey Review and the Way Forward. Journal of Pathology Informatics 2024;15:100357.
3. Qin L, Yang X, Xu X, Zhu Z. A Survey of Deep Learning in Histopathological Nuclear Segmentation. IEEE Computational Intelligence Magazine 2025 Aug;20(3):19–40.
4. Shafi S, Kellough DA, Lujan G, Satturwar S, Parwani AV, Li Z. Integrating and Validating Automated Digital Imaging Analysis of Estrogen Receptor Immunohistochemistry in a Fully Digital Workflow for Clinical Use. Journal of Pathology Informatics 2022;13:100122.
5. Wu S, Yue M, Zhang J, Li X, Li Z, Zhang H, et al. The Role of Artificial Intelligence in Accurate Interpretation of HER2 Immunohistochemical Scores 0 and 1+ in Breast Cancer. Modern Pathology 2023;36(3):100054.
6. Holder AM, Dedeilia A, Sierra-Davidson K, Cohen S, Liu D, Parikh A, et al. Defining clinically useful biomarkers of immune checkpoint inhibitors in solid tumours. Nature Reviews Cancer 2024 Jul;24(7):498–512.
7. Graham S, Vu QD, Raza SEA, Azam A, Tsang YW, Kwak JT, et al. Hover-Net: Simultaneous Segmentation and Classification of Nuclei in Multi-Tissue Histology Images. Medical Image Analysis 2019;58:101563.
8. Chen J, Lu Y, Yu Q, Luo X, Adeli E, Wang Y, et al., TransUNet: Transformers Make Strong Encoders for Medical Image Segmentation. arXiv; 2021.
9. Zhao B, Chen X, Li Z, Yu Z, Yao S, Yan L, et al. Triple U-net: Hematoxylin-aware Nuclei Segmentation with Progressive Dense Feature Aggregation. Medical Image Analysis 2020;65:101786.
10. Cao H, Wang Y, Chen J, Jiang D, Zhang X, Tian Q, et al. Swin-Unet: Unet-like Pure Transformer for Medical Image Segmentation. In: Computer Vision – ECCV 2022 Workshops Cham: Springer Nature Switzerland; 2023. p. 205–218.
11. Guan B, Chu G, Wang Z, Li J, Yi B. Instance-level semantic segmentation of nuclei based on multimodal structure encoding. BMC Bioinformatics 2025 Feb;26(1):42.
12. Mahanta LB, Hussain E, Das N, Kakoti L, Chowdhury M. IHC-Net: A Fully Convolutional Neural Network for Automated Nuclear Segmentation and Ensemble Classification for Allred Scoring in Breast Pathology. Applied Soft Computing 2021;103:107136.

13. Wang R, Qiu Y, Hao X, Jin S, Gao J, Qi H, et al. Simultaneously segmenting and classifying cell nuclei by using multi-task learning in multiplex immunohistochemical tissue microarray sections. Biomedical Signal Processing and Control 2024 Jul;93:106143.
14. He Z, Jia D, Shi Y, Jiang H, Zhang C, Li Z, et al. An immunohistochemical scoring network based on multi-branch and dual attention mechanisms for the evaluation of biomarker PCNA in esophageal cancer. Applied Soft Computing 2025 Jan;169:112636.
15. Xu S, Li G, Song H, Wang J, Wang Y, Li Q. GeNSeg-Net: A General Segmentation Framework for Any Nucleus in Immunohistochemistry Images. In: Proceedings of the 32nd ACM International Conference on Multimedia New York, NY, USA: Association for Computing Machinery; 2024. p. 4475–4484.
16. Wang H, Yang J, Katayama R, Matsusaki M, Miyao T, Zhou J. NuclSeg: nuclei segmentation using semi-supervised stain deconvolution. In: Proceedings of the 5th ACM International Conference on Multimedia in Asia (MMAsia '23) New York, NY, USA: Association for Computing Machinery; 2024. p. 1–6.
17. Yao Y, Hu Y, Xue Y, Li S, Huang J, Wang H, et al. UPHGAN: Generative Adversarial Network Based on Unet512 and PatchGAN Fusion with Huber Loss Function for Immunohistochemical Cell Nucleus Segmentation. In: Neural Information Processing (ICONIP 2024), Communications in Computer and Information Science Singapore: Springer Nature; 2025. p. 88–101.
18. Trullo R, Bui QA, Tang Q, Olfati-Saber R. Image Translation Based Nuclei Segmentation for Immunohistochemistry Images. In: Deep Generative Models Cham: Springer Nature Switzerland; 2022. p. 87–96.
19. Wang S, Rong R, Gu Z, Fujimoto J, Zhan X, Xie Y, et al. Unsupervised Domain Adaptation for Nuclei Segmentation: Adapting from Hematoxylin & Eosin Stained Slides to Immunohistochemistry Stained Slides Using a Curriculum Approach. Computer Methods and Programs in Biomedicine 2023;241:107768.
20. Zurek N, Zhang Y, McGovern DPB, Walts AE, Gertych A. Immunohistochemistry annotations enhance AI identification of lymphocytes and neutrophils in digitized H&E slides from inflammatory bowel disease. Computer Methods and Programs in Biomedicine 2024 Dec;257:108423.
21. Verma R, Kumar N, Patil A, Kurian NC, Rane S, Graham S, et al. MoNuSAC2020: A Multi-Organ Nuclei Segmentation and Classification Challenge. IEEE Transactions on Medical Imaging 2021;40(12):3413–3423.
22. Gamper J, Koohbanani NA, Benes K, Graham S, Jahanifar M, Khurram SA, et al., PanNuke Dataset Extension, Insights and Baselines. arXiv; 2020.
23. Wan Ahmad WSHM, Hasan MJ, Fauzi MFA, Lee JTH, Khor SY, Looi LM, et al. Nuclei Classification in ER-IHC Stained Histopathology Images Using Deep Learning Models. In: TENCON 2022 - 2022 IEEE Region 10 Conference (TENCON) Hong Kong, Hong Kong: IEEE; 2022. p. 1–5.
24. Kirillov A, Mintun E, Ravi N, Mao H, Rolland C, Gustafson L, et al., Segment Anything. arXiv; 2023.
25. Chen RJ, Ding T, Lu MY, Williamson DFK, Jaume G, Song AH, et al. Towards a general-purpose foundation model for computational pathology. Nature Medicine 2024 Mar;30(3):850–862.
26. Zhao HJ, Pa F, Shi YC, Luo X, Ren RR, Peng LH, et al. Prognostic Significance of CD47 in Human Malignancies: A Systematic Review and Meta-Analysis. Translational Cancer Research 2018;7(3).
27. Yu L, Ding Y, Wan T, Deng T, Huang H, Liu J. Significance of CD47 and Its Association With Tumor Immune Microenvironment Heterogeneity in Ovarian Cancer. Frontiers in Immunology 2021;12.
28. Lee MKI, Rabindranath M, Faust K, Yao J, Gershon A, Alsafwani N, et al. Compound Computer Vision Workflow for Efficient and Automated Immunohistochemical Analysis of Whole Slide Images. Journal of Clinical Pathology 2023;76(7):480–485.
29. Zhang M, Wang L, Gu L, Li Z, Wang Y, Ling T, et al., SAM2-PATH: A better segment anything model for semantic segmentation in digital pathology. arXiv; 2024.
30. Chen T, Lu A, Zhu L, Ding C, Yu C, Ji D, et al., SAM2-Adapter: Evaluating & Adapting Segment Anything 2 in Downstream Tasks: Camouflage, Shadow, Medical Image Segmentation, and More. arXiv; 2024.
31. Woo S, Park J, Lee JY, Kweon IS, CBAM: Convolutional Block Attention Module. arXiv; 2018.
32. Huang Z, Ding Y, Song G, Wang L, Geng R, He H, et al. BCData: A Large-Scale Dataset and Benchmark for Cell Detection and Counting. In: Medical Image Computing and Computer Assisted Intervention – MICCAI 2020 Cham: Springer International Publishing; 2020. p. 289–298.
33. Ghahremani P, Li Y, Kaufman A, Vanguri R, Greenwald N, Angelo M, et al. Deep learning-inferred multiplex immunofluorescence for immunohistochemical image quantification. Nature Machine Intelligence 2022 Apr;4(4):401–412.
34. Mouelhi A, Rmili H, Ali JB, Sayadi M, Doghri R, Mrad K. Fast Unsupervised Nuclear Segmentation and Classification Scheme for Automatic Allred Cancer Scoring in Immunohistochemical Breast Tissue Images. Computer Methods and Programs in Biomedicine 2018;165:37–51.
35. Priego-Torres BM, Lobato-Delgado B, Atienza-Cuevas L, Sanchez-Morillo D. Deep Learning-Based Instance Segmentation for the Precise Automated Quantification of Digital Breast Cancer Immunohistochemistry Images. Expert Systems with Applications 2022;193:116471.
36. Liakopoulos P, Massonnet J, Bonjour J, Mizrakli MT, Graham S, Cuendet MA, et al., HoverFast: an accurate, high-throughput, clinically deployable nuclear segmentation tool for brightfield digital pathology images. arXiv; 2024.
37. Weigert M, Schmidt U. Nuclei Instance Segmentation and Classification in Histopathology Images with Stardist. In: 2022 IEEE International Symposium on Biomedical Imaging Challenges (ISBIC); 2022. p. 1–4.
38. Pachitariu M, Rariden M, Stringer C, Cellpose-SAM: superhuman generalization for cellular segmentation. bioRxiv; 2025.
39. Taha AA, Hanbury A. Metrics for evaluating 3D medical image segmentation: analysis, selection, and tool. BMC Medical Imaging 2015 Aug;15(1):29.
40. Vu QD, Graham S, Kurc T, To MNN, Shaban M, Qaiser T, et al. Methods for Segmentation and Classification of Digital Microscopy Tissue Images. Frontiers in Bioengineering and Biotechnology 2019 Apr;7:53.
41. Kumar N, Verma R, Sharma S, Bhargava S, Vahadane A, Sethi A. A Dataset and A Technique for Generalized Nuclear Segmentation for Computational Pathology. IEEE Trans Med Imaging 2017;36(7):1550–1560.
42. Kirillov A, He K, Girshick R, Rother C, Dollar P. Panoptic Segmentation. In: Proceedings of the IEEE/CVF Conference on Computer Vision and Pattern Recognition; 2019. p. 9404–9413.
43. Zhang S, Yuan Z, Zhou X, Wang H, Chen B, Wang Y. VENet: Variational energy network for gland segmentation of pathological images and early gastric cancer diagnosis of whole slide images. Computer Methods and Programs in Biomedicine 2024 Jun;250:108178.
