# Supplementary material for: FPNuNet: a frequency-aware prompt-guided network for nuclear segmentation and classification in immunohistochemistry images
Source: Gigascience. 2026 Sep 7;15:giag091. doi: 10.1093/gigascience/giag091 (PMC13618258; doi:10.1093/gigascience/giag091)
Supplement: Supplementary file 3 [file giag091_giga-d-25-00479_revision_1.pdf]

# GigaScience

## FPNuNet: A Frequency-Aware Prompt-Guided Network for Nuclear Segmentation and Classification in Immunohistochemistry Images

--Manuscript Draft--

| | |
|---|---|
| **Manuscript Number:** | GIGA-D-25-00479R1 |
| **Full Title:** | FPNuNet: A Frequency-Aware Prompt-Guided Network for Nuclear Segmentation and Classification in Immunohistochemistry Images |
| **Article Type:** | Research |
| **Funding Information:** | National Key Technologies Research and Development Program of China (2022YFF1202104) — Prof. Zexuan Zhu<br>National Natural Science Foundation of China (62471310) — Prof. Zexuan Zhu |
| **Abstract:** | Accurate nuclear segmentation and classification (NuSC) in immunohistochemistry (IHC)-stained whole-slide images is essential for reliable biomarker quantification in computational pathology. Although existing NuSC methods perform well on Hematoxylin and Eosin images, they frequently underperform on IHC-stained images owing to stain heterogeneity, low nuclear contrast, and scarce biomarker-specific annotations. Prior efforts based on stain normalization or cross-domain knowledge transfer have yielded only marginal improvements under these conditions. To address these limitations, we introduce FPNuNet, a frequency-aware prompt-guided network tailored for NuSC of IHC-stained images. FPNuNet extracts multi-source features through four parallel encoders: a frozen SAM-based structural encoder and a frozen UNI-based semantic encoder---both adapted via lightweight discrete cosine transform-based prompt generators---together with a wavelet feature encoder and a multi-scale context encoder that capture complementary spatial and frequency-domain descriptors from the raw input. A discrete cosine transform-enabled frequency-aware fusion neck integrates all four feature streams with spectral enhancement, and three collaborative decoder branches jointly predict binary masks, horizontal--vertical vectors, and nuclear types. FPNuNet is evaluated on CD47-IHCNuSC, a dataset comprising 86 CD47-stained esophageal cancer patches with 18,483 manually annotated nuclei spanning seven clinically meaningful subtypes. On CD47-IHCNuSC, FPNuNet achieves the best instance-segmentation and aggregate subtype-classification performance among all evaluated methods under challenging IHC staining conditions. |
| **Corresponding Author:** | Zexuan Zhu<br>Shenzhen University<br>Shenzhen, CHINA |
| **Corresponding Author Secondary Information:** | |
| **Corresponding Author's Institution:** | Shenzhen University |
| **Corresponding Author's Secondary Institution:** | |
| **First Author:** | Lulu Qin |
| **First Author Secondary Information:** | |
| **Order of Authors:** | Lulu Qin |
| | Zhigang Pei |
| | Xudong He |
| | Jiarui Zhou |
| | Xianhong Xu |
| | Zexuan Zhu |

Powered by Editorial Manager® and ProduXion Manager® from Aries Systems Corporation

| | |
|---|---|
| **Order of Authors Secondary Information:** | |
| **Response to Reviewers:** | Dear Editor and Reviewers,<br><br>We sincerely thank the Editor and both Reviewers for their careful and thorough evaluation of our manuscript. The critiques are substantive and have considerably sharpened our thinking about the scope and limitations of this work. We are particularly grateful for the Reviewers' recognition of the dataset's value to the community, and we regard the methodological concerns—regarding baseline fairness, statistical rigor, architectural justification, and dataset accessibility—as precisely the kind of constructive challenge that strengthens published science.<br><br>In this revision, we have addressed every comment with one of three approaches: (1) corrected or expanded manuscript text where the existing evidence was sufficient but the writing was unclear or misleading; (2) new experimental evidence where additional data were available; and (3) honest acknowledgment of limitations where the current study cannot fully resolve a concern.<br><br>All section/table references below refer to the revised manuscript.<br><br>Editor Requirements<br><br>EQ1. Register the software on bio.tools and SciCrunch.org to obtain an RRID and a biotoolsID, and cite these in the manuscript.<br><br>Response: Done. FPNuNet is registered on SciCrunch (RRID: SCR_027659) and bio.tools (biotools:fpnunet). Both identifiers are cited in the revised "Availability of Source Code and Requirements" section.<br><br>EQ2. Register the computational workflow on workflowhub.eu and cite the DOI in the manuscript.<br><br>Response: Done. The analysis workflow is deposited on WorkflowHub (DOI: 10.48546/workflowhub.workflow.2166.1). The DOI is cited in the revised "Availability of Source Code and Requirements" section.<br><br>EQ3. Submit a point-by-point response in the "Response to Reviewers" box.<br><br>Response: This document constitutes the point-by-point response.<br><br>EQ4. If data or code have been modified, update the public repository.<br><br>Response: The public GitHub repository (https://github.com/LuluQin766/FPNuNet) has been updated to include corrected model descriptions, evaluation scripts, and the CD47-IHCNuSC dataset with train/test split identifiers.<br><br>EQ5. Ensure the revised manuscript follows all journal format requirements.<br><br>Response: We have verified compliance with the GigaScience author guidelines. The revised manuscript conforms to the required structure, reference style, and formatting.<br><br>Response to Reviewer #1<br><br>We are grateful to Reviewer #1 for a highly structured and technically demanding review. The five major concerns—cross-domain generalization, baseline fairness, dataset accessibility, annotation quality control, and ablation granularity—each identify a genuine gap between the paper's claims and the evidence provided. We address them in full below.<br><br>R1-Q1: Generalization beyond a single biomarker and tumor site<br>The dataset contains only a single biomarker (CD47) and a single tumor site, with a limited number of WSIs and patches. The review requires: (a) external validation on different cohorts/institutions/scanners;<br>(b) a clear distinction between in-dataset performance and cross-domain generalization claims. |

Powered by Editorial Manager® and ProduXion Manager® from Aries Systems Corporation

Response: We thank the reviewer for raising this important concern. We agree that the current dataset is limited to one biomarker and one tumor site, and therefore does not support broad cross-domain generalization claims.
What we have done:
1. We revised the Abstract, Introduction, Results, and Discussion to clearly restrict our performance claims to the CD47-IHCNuSC benchmark and to avoid implying generalization across biomarkers, tumor types, institutions, or scanners.
2. We examined publicly available IHC nuclear analysis datasets for possible external validation. However, most existing IHC resources are either not publicly accessible, provide only server-side evaluation, or are designed for different tasks such as point-level detection, binary positivity as-sessment, or H&E-based nuclear segmentation. To the best of our knowledge, no public IHC dataset currently provides instance-level nuclear segmentation masks with subtype-level classifi-cation labels compatible with the NuSC evaluation protocol used in this study.
3. We therefore acknowledge the lack of external validation as a major limitation rather than presenting CD47-IHCNuSC results as evidence of cross-domain robustness. The Discussion has been revised to explicitly state that future validation should include multi-institutional cohorts, additional scanners, additional biomarkers, and other tumor types.
4. We also added future directions on domain-adaptive fine-tuning, stain augmentation, and the ex-tension of CD47-IHCNuSC to broader IHC settings.
Accordingly, the revised manuscript positions CD47-IHCNuSC as an initial benchmark for IHC-based nuclear segmentation and classification, rather than as evidence of universal generalization across IHC domains.
[Manuscript change] Revised Abstract (scope qualification); new Limitations paragraph on generalization in Discussion; honest acknowledgment that cross-dataset validation is precluded by the current unavailability of compatible public IHC NuSC benchmarks.

R1-Q2: Baseline comparison fairness
Some baselines use publicly pretrained weights without fine-tuning, while other models use the paper’s training protocol. This creates an unfair comparison.

Response: This concern is well-founded and overlaps with R2-Q5.
What we have done:
1. In the revised manuscript, we have retrained HoverFast and Cellpose-SAM on the CD47-IHCNuSC training split under the same training protocols as FPNuNet, HoVer-Net, and SRSA-Net. Only StarDist remains evaluated with its publicly released pretrained model as an off-the-shelf instance segmentation baseline, because its TensorFlow backend is incompatible with our PyTorch training pipeline.
2. The revised Experimental Setup now explicitly states the training condition for each baseline (Section 3.1): “the FPNuNet series, HoVer-Net, SRSA-Net, HoverFast, and Cellpose-SAM were trained on the same CD47-IHCNuSC training split using consistent training and evaluation protocols whenever applicable. StarDist was evaluated with its publicly released pretrained model.”
3. StarDist’s training condition is transparently stated in the Experimental Setup; the large performance gap (e.g., AJI 0.450 vs. FPNuNet’s 0.747) reflects both architectural limitations and the absence of domain adaptation, and does not affect the ranking among fine-tuned methods.
[Manuscript change] Experimental Setup: HoverFast and Cellpose-SAM retrained on CD47-IHCNuSC; explicit training-condition statement for each baseline.

R1-Q3: Reproducibility and data access
The dataset was private during review. The review requires: preprocessing/slicing code, exact train/test split identifiers, evaluation code, and a clear public release plan.

Response: We apologize for the dataset not being publicly accessible during the initial review. The CD47-IHCNuSC dataset—including image patches, instance-level masks, nuclear type maps, and metadata—is now publicly hosted in the project repository: https://github.com/LuluQin766/FPNuNet/tree/ main/CD47_IHCNUSC. Train/test split identifiers and evaluation scripts are included.
[Manuscript change] Revised “Data Availability” section with public repository URL.

R1-Q4: Annotation reliability and rare-class error patterns

Powered by Editorial Manager® and ProduXion Manager® from Aries Systems Corporation

Human-in-the-loop annotation is described but no quantitative quality control is provided. The review requires: inter-annotator agreement metrics, adjudication rate, error pattern analysis for rare classes, and analysis of class imbalance effects.

Response: The Reviewer correctly identifies that the annotation protocol description lacks quantitative grounding.
What we have done:
1. The Methods section (Section 2.1) describes the full annotation pipeline: HoverFast-based prelabeling, manual refinement in QuPath by trained scientists, and adjudication by board-certified pathologists.
2. A new “Annotation quality” paragraph in the Discussion transparently acknowledges that no formal inter-annotator agreement (e.g., Cohen’s κ) or adjudication-rate statistics were recorded during the annotation campaign. We identify this as a limitation and recommend multi-rater protocols with explicit agreement quantification for future annotation efforts.
3. Residual label noise is discussed as most likely concentrated at morphologically ambiguous boundaries (immune-related vs. stromal nuclei) and in rare subtypes, contributing to the performance ceiling on minority classes.
4. The effects of class imbalance are addressed in a dedicated “Class imbalance” paragraph in the Discussion (see also R2-Q1).
[Manuscript change] Discussion: new “Annotation quality” paragraph acknowledging the absence of quantitative inter-annotator agreement data; new “Class imbalance” paragraph with per-class analysis.

R1-Q5: Ablation clarity and complexity justification
The frequency (DCT/wavelet) and prompt components are complex. The ablation is insufficiently fine-grained. The review requires: targeted ablations isolating each component; statistical confidence across slides; runtime/memory/latency analysis.

Response: We agree that the original ablation presentation was insufficient.
What we have done:
1. A new progressive ablation table (Table 4) traces the contribution of each module (Adapter, Type Decoder, DCT-PFAE/SkipNeck). The Results text explicitly acknowledges that the final step couples three changes and notes this as a limitation.
2. A new Frequency Transform Ablation subsection (Section 3.5, Table 7) isolates the effect of trans-form selection by comparing four variants on CD47-IHCNuSC.
3. Table 2 reports THOP-measured FLOPs and parameter counts for all baselines with available profiling (HoverFast, HoVer-Net, Cellpose-SAM, FPNuNet). Latency and memory are reported in the accompanying prose. A revised “Computational complexity” paragraph in the Discussion frames FPNuNet as a parameter-efficient adaptation framework.
4. Statistical power limitations (N=16) are addressed in a new Discussion paragraph (see R2-Q2).
5. To provide the requested statistical confidence across slides, we now report paired per-image
bootstrap 95% confidence intervals (10,000 resamples) and Holm–Bonferroni-corrected Wilcoxon signed-rank tests for all pairwise comparisons in the Results (Section 3.3). On AJI, FPNuNet significantly outperforms every baseline and ablation variant (corrected $p < 0.05$). Corresponding
CIs and p-values are reported for bPQ, SQ, DQ, Dice1, and Dice2, with non-significant differences explicitly acknowledged (e.g., bPQ vs. HoVer-Net: $p = 0.28$).
[Manuscript change] New Table 4 (progressive ablation); new Table 7 (frequency transform ablation); Table 2 (model complexity for four baselines); DCT coupling paragraph in Results; revised “Computational complexity” paragraph in Discussion; new paired statistical analysis (bootstrap CI + Wilcoxon) in Results Section 3.3.

Response to Reviewer #2

We thank Reviewer #2 for a rigorous and technically precise review. While affirming the dataset’s value, Reviewer #2 raises pointed concerns regarding statistical credibility, architectural proportionality, and a factual error in a complexity claim. We accept several of these criticisms unreservedly and address each below.

R2-Q1: Class imbalance insufficiently addressed

Severe class imbalance (pTu 40.5%, multiple classes <1%) is not sufficiently addressed. Only 4 of 7 classes are reported in Table 3. Standard focal loss cannot fundamentally solve such severe imbalance.

Response: Reviewer #2 is correct on all counts. We acknowledge that focal loss alone cannot adequately address imbalance of this magnitude, and the omission of three classes from Table 3 was a significant oversight that we have now corrected.
Why only 4 of 7 classes were originally reported: Three categories (pOth, nOth, nSt) are either extremely rare or morphologically ambiguous, providing insufficient test-set representation for reliable per-class evaluation. They are merged into pTu and nTu prior to training, reducing the taxonomy to four foreground classes. The original manuscript did not describe this merging decision, which we acknowledge as an oversight.
What we have done:
1. The revised Methods section (Section 2.2) now explicitly describes the class merging, its rationale, and the resulting four-class taxonomy.
2. The loss configuration (focal $\gamma$=2.0, uniform foreground weights, no inverse-frequency weighting) is stated explicitly.
3. A new per-class classification table (Table 6) reports PQ/P/R/F1 for all four foreground subtypes across all models. Table 5 is updated accordingly.
4. A new “Class imbalance” paragraph in the Discussion analyses per-class trends, acknowledges remaining gaps on minority subtypes, and identifies inverse-frequency weighting and prototype-based learning as future directions.
[Manuscript change] Revised Methods (class merging + loss specification); new per-class Table 6 with four foreground classes; updated Table 5 (overall metrics); new “Class imbalance” paragraph in Discussion.

R2-Q2: No statistical testing, N=16
There is no statistical testing with only 16 test images. Modest performance improvements may reflect random variation.

Response: We agree that N=16 constitutes a small test set and that modest performance differences may reflect random variation.
What we have done:
1. We performed paired statistical testing based on per-image metric values. Pairwise comparisons between FPNuNet and each competing method were assessed using Wilcoxon signed-rank tests with Holm–Bonferroni correction for multiple comparisons (Results, Section 3.3).
2. Performance claims in the Abstract, Introduction, and Results have been qualified to reflect the limited sample size. Where margins are small or statistically non-significant, observed differences are framed as trends rather than definitive rankings.
[Manuscript change] Paired Wilcoxon signed-rank tests with Holm–Bonferroni correction added to Results Section 3.3; revised “Statistical power” paragraph in the Discussion; scope-qualified performance claims throughout.

R2-Q3: Complexity vs. performance gain
Limited performance gains relative to large architectural complexity increase. Paper acknowledges memory and latency issues but lacks quantitative analysis.

Response: This is a substantive architectural critique, and we accept its core premise.
What we have done:
1. Table 2 reports THOP-measured FLOPs and parameter counts for all profiled baselines; latency and memory are discussed in the accompanying prose (see also R1-Q5).
2. The revised “Computational complexity” paragraph in the Discussion frames FPNuNet as a parameter-efficient adaptation framework rather than a lightweight inference model, and suggests smaller pretrained encoders as a path to faster deployment.
3. Regarding cross-dataset evaluation, please see our detailed response in R1-Q1: despite extensive efforts, no publicly available IHC dataset with compatible instance-level NuSC annotations could be identified. This limitation is acknowledged in the revised Discussion.
[Manuscript change] Table 2 (model complexity for four baselines); revised “Computational complexity” paragraph in Discussion; revised Limitations paragraph

| | |
|---|---|
| | acknowledging the absence of cross-dataset validation.<br><br>R2-Q4: DCT vs. FFT complexity claim is incorrect<br>The claim "DCT provides real-valued frequency representation without FFT complexity" is factually wrong—both are O(n log n). FFT may be faster. Non-DCT changes may be the actual performance source.<br><br>Response: We accept this correction unreservedly. The original statement was factually incorrect and should not have appeared in the manuscript.<br>What we have done:<br>1. The incorrect complexity claim has been removed. The revised Frequency-Aware Fusion Modules description (Section 2.2) now justifies the DCT choice on two accurate grounds: (a) energy compaction—DCT concentrates spectral energy of smooth, localized structures into fewer low-frequency components, providing a compact spectral basis for IHC nuclear textures; and (b) real-valued coefficients—DCT eliminates the complex-number bookkeeping (magnitude/phase storage, complex-to-real conversion) that FFT would impose, simplifying the gradient path. These are implementation and representation advantages, not computational complexity reductions.<br>2. The revised Methods paragraph describes DCT's role uniformly at both the encoder adaptation and post-encoder fusion stages, with empirical validation of this design choice in the Frequency Transform Ablation (Section 3.5).<br>3. A new Frequency Transform Ablation subsection (Section 3.5, Table 7) isolates the effect of trans-form selection by comparing four variants on CD47-IHCNuSC, confirming the DCT choice empirically.<br>4. The Results text acknowledges that the final ablation step couples three changes and notes this as a limitation (see also R1-Q5).<br>[Manuscript change] Corrected PFAE Block text in Methods (Section 2.2); new frequency processing overview paragraph (Section 2.2); Table 4 (progressive ablation); new Table 7 (frequency transform ablation, Section 3.5); new DCT coupling discussion paragraph in Results.<br><br>R2-Q5: Baseline comparison not apples-to-apples<br>CellPose and similar models were not fine-tuned on the authors' dataset. Even without fine-tuning, FPNuNet's advantage is marginal.<br><br>Response: This concern overlaps directly with R1-Q2, and we accept the criticism.<br>What we have done:<br>1. In the revised manuscript, HoverFast and Cellpose-SAM have been retrained on the CD47-IHCNuSC training split using the same protocols as the other baselines. Only StarDist remains evaluated with its pretrained weights (TensorFlow backend incompatibility).<br>2. The revised Experimental Setup now explicitly states the training condition for each baseline.<br>3. StarDist's training condition is transparently stated in the Experimental Setup; the large performance gap does not affect conclusions among fine-tuned methods.<br>See R1-Q2 for full details.<br>[Manuscript change] Same as R1-Q2. |
| **Additional Information:** | |
| **Question** | **Response** |
| Are you submitting this manuscript to a special series or article collection? | No |
| **Experimental design and statistics**<br><br><br>Full details of the experimental design and statistical methods used should be given in the Methods section, as detailed in our Minimum Standards Reporting Checklist. Information essential to interpreting the | Yes |

| | |
|---|---|
| data presented should be made available in the figure legends.<br><br>Have you included all the information requested in your manuscript? | |
| **Resources**<br><br>A description of all resources used, including antibodies, cell lines, animals and software tools, with enough information to allow them to be uniquely identified, should be included in the Methods section. Authors are strongly encouraged to cite Research Resource Identifiers (RRIDs) for antibodies, model organisms and tools, where possible.<br><br>Have you included the information requested as detailed in our Minimum Standards Reporting Checklist? | Yes |
| **Availability of data and materials**<br><br>All datasets and code on which the conclusions of the paper rely must be either included in your submission or deposited in publicly available repositories (where available and ethically appropriate), referencing such data using a unique identifier in the references and in the "Availability of Data and Materials" section of your manuscript.<br><br>Have you have met the above requirement as detailed in our Minimum Standards Reporting Checklist? | Yes |
| GigaScience has policies and guidelines in place for the use of generative AI-writing tools such as ChatGPT. If you have used such writing tools to assist with writing the manuscript this must be declared and cited in the text. Authors should not list AI-writing tools and other AI-assisted technologies as an author or co-author and should acknowledge that they are fully responsible for text | No |

| | |
|---|---|
| generated or refined by AI-writing tools.<p><br><br>A summary of use (particularly in the introduction or among methods) needs to be included at the end of the paper, and the outputs should also be included as a supplementary file hosted in GigaDB or other open repositories. Please <a href=https://academic.oup.com/gigascience/pages/editorial_policies_and_reporting_standards target="_new" > read our guidelines for more information. </a> <p><br><br>By submitting to GigaScience, you are aware of the journal's AI-writing tools policy, and if you have declared use of such tools below, you have acknowledged this where appropriate in your manuscript and have made a summary of use and outputs available. </b><p><br><b>AI-assisted writing tools have been used in the preparation of this manuscript? | |

Powered by Editorial Manager® and ProduXion Manager® from Aries Systems Corporation

Manuscript
Click here to access/download;Manuscript;main.pdf

Placeholder for OUP logo oup.pdf

*GigaScience*, 2025, 1–15

doi: xx.xxxx/xxxx
**Manuscript in Preparation**
**Paper**

PAPER

# FPNuNet: A Frequency-Aware Prompt-Guided Network for Nuclear Segmentation and Classification in Immunohistochemistry Images

Lulu Qin[1], Zhigang Pei[2], Xudong He[3], Jiarui Zhou[4], Xianhong Xu[5] and Zexuan Zhu[3,*]

[1]College of Computer Science and Software Engineering, Shenzhen University, China and [2]Pathology Department, Chongqing University Jiangjin Hospital, China and [3]School of Artificial Intelligence, Shenzhen University, China and [4]School of Biosciences, University of Birmingham, UK and [5]Department of Oncology, University of Oxford, UK

[*]Corresponding author. zhuzx@szu.edu.cn

## Abstract

Accurate nuclear segmentation and classification (NuSC) in immunohistochemistry (IHC)-stained whole-slide images is essential for reliable biomarker quantification in computational pathology. Although existing NuSC methods perform well on Hematoxylin and Eosin images, they frequently underperform on IHC-stained images owing to stain heterogeneity, low nuclear contrast, and scarce biomarker-specific annotations. Prior efforts based on stain normalization or cross-domain knowledge transfer have yielded only marginal improvements under these conditions. To address these limitations, we introduce FPNuNet, a frequency-aware prompt-guided network tailored for NuSC of IHC-stained images. FPNuNet extracts multi-source features through four parallel encoders: a frozen SAM-based structural encoder and a frozen UNI-based semantic encoder—both adapted via lightweight discrete cosine transform-based prompt generators—together with a wavelet feature encoder and a multi-scale context encoder that capture complementary spatial and frequency-domain descriptors from the raw input. A discrete cosine transform-enabled frequency-aware fusion neck integrates all four feature streams with spectral enhancement, and three collaborative decoder branches jointly predict binary masks, horizontal–vertical vectors, and nuclear types. FPNuNet is evaluated on CD47-IHCNuSC, a dataset comprising 86 CD47-stained esophageal cancer patches with 18,483 manually annotated nuclei spanning seven clinically meaningful subtypes. On CD47-IHCNuSC, FPNuNet achieves the best instance-segmentation and aggregate subtype-classification performance among all evaluated methods under challenging IHC staining conditions.

**Key words**: Medical Imaging; Computational Pathology; Nuclear Segmentation and Classification; Immunohistochemistry; Frequency-Aware Fusion

## Introduction

Automated histopathology is reshaping clinical workflows by enabling reproducible, scalable biomarker quantification and large-cohort analysis [1, 2]. Robust nuclear segmentation and classification (NuSC) is central to these pipelines, underpinning cell-level readouts (e.g., counts, densities, and phenotypes) that inform diagnosis, prognosis, and treatment monitoring [3]. Yet clinical slide heterogeneity and lab-specific protocols demand NuSC models that remain accurate across staining variability and acquisition differences [4, 5, 6].

Deep learning has driven rapid advances in NuSC on Hematoxylin and Eosin (H&E) slides, enabled by sustained algorithmic innovation and the standardization of evaluation on widely adopted public benchmarks. Representative architectures such as HoVer-Net [7] unify pixel-wise masks with horizontal–vertical distance regression to split touching instances while enabling joint nuclear

**Compiled on:** August 25, 2026.
Draft manuscript prepared by the author.

**Key Points**

- We propose FPNuNet, a frequency-aware, prompt-guided multi-encoder framework for nuclear segmentation and classification in immunohistochemistry (IHC)-stained pathology images that combines frozen pretrained backbones with lightweight wavelet and multi-scale encoders.
- A new benchmark dataset, CD47-IHCNuSC, is curated for biomarker-aware analysis of CD47-stained esophageal carcinoma.
- Evaluations on CD47-IHCNuSC demonstrate that FPNuNet improves several instance-segmentation and subtype-classification metrics over the compared baselines under challenging IHC conditions.

typing. TransUNet [8] combines a transformer encoder with a U-Net style decoder to fuse long-range dependencies with multi-scale detail. Triple U-Net [9] adds hematoxylin-aware pathways and progressive dense aggregation to stabilize boundaries and reduce type confusion. Swin-UNet [10] employs hierarchical windowed self-attention with shifted windows to capture multi-scale context efficiently. Collectively, these designs achieve reliable instance delineation and nuclear typing on H&E slides, yet they are optimized for H&E-specific stain appearance and morphology, which limits direct transfer to immunohistochemistry (IHC)-stained images [11].

IHC introduces heterogeneous 3,3′-diaminobenzidine (DAB) deposition that alters local spectral characteristics and attenuates the hematoxylin counterstain, thereby blurring nuclear boundaries and reducing intra-nuclear contrast. Variation in antibody selection and staining protocols further reshapes tissue texture and class distribution, amplifying ambiguity among positive, weak-positive, and negative phenotypes. Together, these domain shifts produce characteristic errors—boundary leakage, over- or under-segmentation, and inconsistent type assignment—motivating the IHC-specific methods discussed below.

Recent IHC-oriented NuSC research has pursued two complementary strategies. The first learns directly on IHC with coupled objectives so that segmentation and phenotype or intensity estimation co-adapt within a single model. For example, IHC-Net [12] combines a fully convolutional network segmenter with watershed splitting and a classical ensemble for intensity scoring on estrogen receptor IHC. SRSA-Net [13] modernizes the triple-head design with a lightweight encoder, decoder-level self-attention, and stitching. PH-ScoreNet [14] uses dual branches and dual attention to predict IHC-scores end-to-end for proliferating cell nuclear antigen. The second reduces the stain gap prior to segmentation through generative translation or morphology normalization. For instance, GeNSeg-Net [15] performs in-domain generative adversarial network (GAN) based enhancement followed by a lightweight U-Net and watershed, achieving strong cross-tissue transfer. NuclSeg [16] bridges from IHC to multiplex immunofluorescence images using semi-supervised stain deconvolution with multi-GAN fusion and thus depends on co-registered pairs. UPHGAN [17] translates IHC to virtual H&E with a U-Net–PatchGAN generator–discriminator trained with a Huber-augmented objective and then fuses masks from both domains. Trullo et al. [18] similarly translate IHC to virtual H&E to leverage H&E-specialized segmenters such as HoVer-Net, although gains for cross-modal models such as StarDist remain modest. ISC-GAN integrates CycleGAN with Mask R-CNN in a curriculum to adapt from labeled H&E to unlabeled IHC [19]. An alternative strategy treats IHC primarily as a supervision source rather than an inference modality, employing destain–restain registration to transfer IHC-derived labels back to H&E and thereby improve immune-cell recognition [20]. Despite tangible gains from task coupling, in-domain enhancement, and cross-stain supervision, progress remains constrained by the absence of publicly accessible, biomarker-specific IHC benchmarks with subtype-level NuSC labels and by the scarcity of architectures explicitly designed for heterogeneous IHC staining conditions.

A further obstacle is the paucity of high-quality, publicly available, and expertly annotated IHC datasets [3]. While established benchmarks such as CoNSeP [7], MoNuSAC [21], and PanNuke [22] have catalyzed advances in H&E-based NuSC, they do not account for IHC-specific complexity—heterogeneous DAB deposition, diminished nuclear contrast, and the absence of biomarker context. By contrast, IHC resources remain fragmented: many widely cited datasets are private or request-only [12, 23, 15, 16, 20, 14], and others are image-only test corpora without publicly released ground truth, with scoring available only via server-side submission [18, 17]. Even where labels exist, they are often restricted to binary positivity rather than per-nucleus subtypes and frequently lack standardized tiling/stitching protocols, complicating fair instance-level evaluation and cross-study replication [13, 17, 18]. In sum, the field still lacks publicly accessible, expert-verified IHC benchmarks with subtype-level annotations and standardized evaluation protocols, suitable for rigorous, reproducible NuSC on IHC.

To address the aforementioned issues and facilitate biomarker-aware NuSC under the severe stain variability of IHC, we present FPNuNet, a frequency-aware, prompt-guided network that employs four parallel encoders—a frozen Segment Anything Model (SAM) structural encoder [24], a frozen UNI pathology semantic encoder [25], a wavelet feature encoder (WFE), and a multi-scale context encoder (MSCE)—and integrates their outputs through a discrete cosine transform (DCT)-enabled frequency-aware fusion neck, as shown in Fig. 1. FPNuNet is evaluated on IHC-stained images of a representative biomarker, the tumor-associated antigen *cluster of differentiation* 47 (CD47). CD47 overexpression enables tumor cells to evade macrophage-mediated phagocytosis and is correlated with aggressive behavior and poor prognosis in esophageal carcinoma and other malignancies [26, 27, 28]. To support this task, we curate CD47-IHCNuSC, an expert-annotated benchmark for CD47-stained esophageal carcinoma. Experiments on CD47-IHCNuSC demonstrate that FPNuNet achieves strong instance-segmentation and subtype-classification performance under challenging IHC staining conditions. The main contributions are as follows:

- We propose FPNuNet, a frequency-aware, prompt-guided architecture for NuSC of IHC-stained images. FPNuNet comprises four parallel encoders (a frozen SAM structural encoder, a frozen UNI pathology encoder, WFE, and MSCE), a DCT-enabled Prompt-guided Frequency-Aware Enhancement (PFAE) fusion neck, and tri-branch decoders for simultaneous binary, horizontal–vertical (HV), and type predictions.
- We release CD47-IHCNuSC, the first publicly available IHC dataset dedicated to CD47-stained esophageal cancer. The dataset comprises 86 high-resolution patches (18,483 nuclei across seven clinically meaningful subtypes), annotated through a semi-automated, pretrained-model-assisted protocol with expert verification.
- Experiments on CD47-IHCNuSC show that FPNuNet achieves the highest AJI and bPQ among all evaluated methods trained on the same benchmark, with progressive ablation confirming complementary contributions from prompt adaptation,

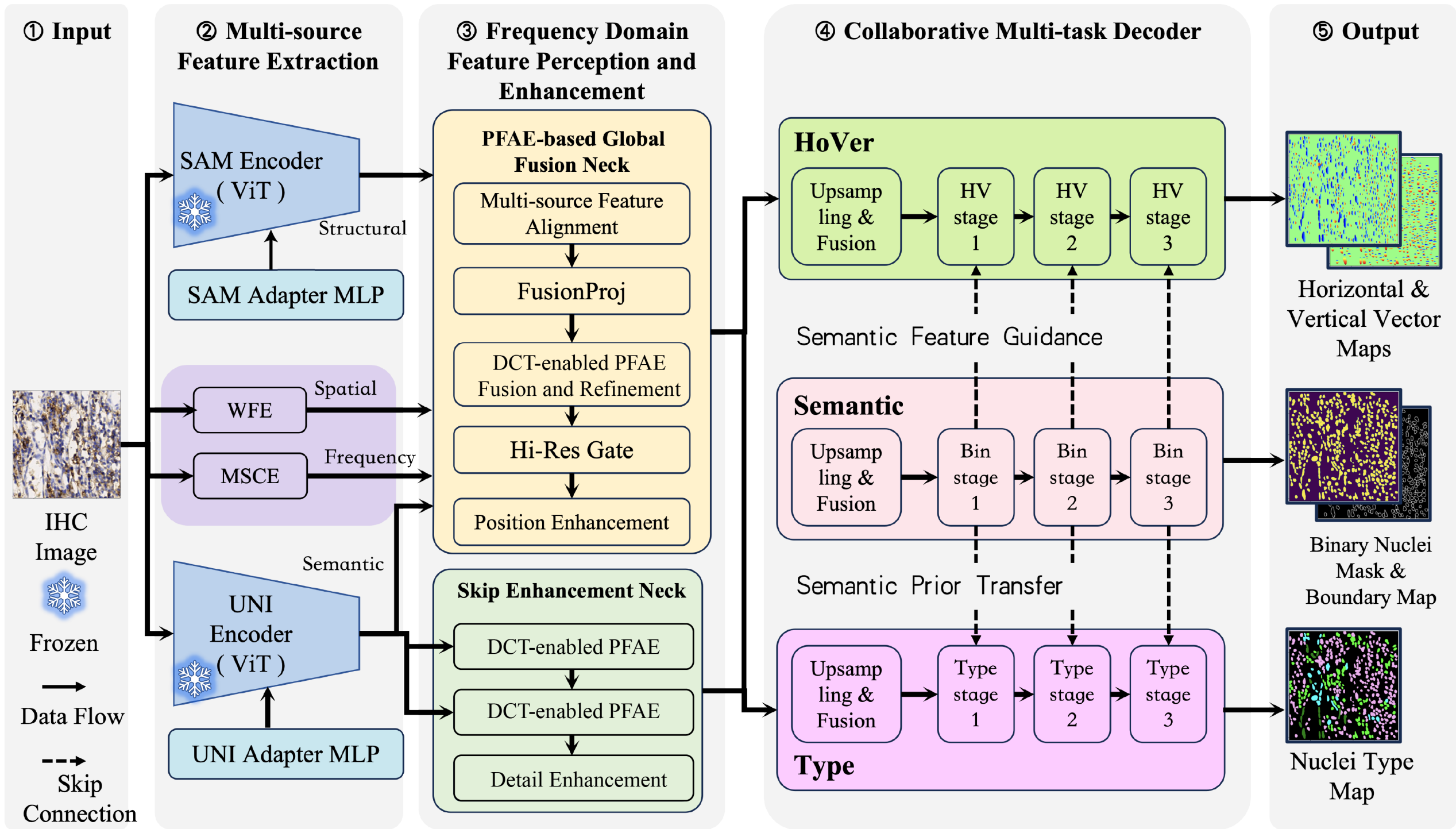


**Figure 1.** Overview of the FPNuNet architecture. RGB input patches are processed by a frozen SAM ViT-B encoder with DCT-based high-pass prompt adaptation, a frozen UNI ViT-L encoder with deep additive prompt injection, and lightweight trainable WFE and MSCE branches. A DCT-enabled four-way PFAE fusion neck integrates all feature streams, while DCT-enabled skip necks refine UNI intermediate features. Three task-specific decoders — binary, HV, and type — are sequentially guided by binary decoder features through gated fusion, jointly predicting nuclei masks, boundaries, HV regression maps, and nuclear type labels.

frequency-aware fusion, and decoder design.

## Materials and Methods

### Data Description

To enable rigorous evaluation of biomarker-aware NuSC under clinically realistic IHC conditions, we construct **CD47-IHCNuSC**, a new benchmark targeting CD47-stained esophageal cancer. Among publicly accessible resources, label granularity is typically limited to detection or binary positivity rather than fine-grained nuclear subtyping—for example, BCData [29] offers point detection with two classes, DeepLIIF [30] provides binary nuclear positivity labels without instance-level delineation, and the SRSA-Net TMA corpus [13] annotates nuclei with only two categories (positive/negative) on breast tissue microarray cores. Other efforts emphasize stain-domain robustness rather than subtype-level NuSC, and several influential works remain non-public or prioritize intensity scoring over subtype taxonomy [12, 23, 31]. **CD47-IHCNuSC** addresses these limitations by targeting esophageal tissue and the CD47 biomarker, providing expert-validated instance masks with seven fine-grained, phenotype-aware nuclear subtypes under realistic IHC variability.

#### *Construction, Annotation, and Quality Control*

All CD47-immunostained esophageal whole-slide images (WSIs) were processed using a DAB chromogen with hematoxylin counterstain and digitized at 0.25 μm/pixel (40×) on an Aperio AT2 scanner. A board-certified pathologist delineated tumor-enriched, morphologically diverse regions; patches with low tissue content were excluded. We sampled **86** non-overlapping 1024 × 1024 patches (see Table 1 for image counts and class distributions). To preserve real-world appearance, no stain normalization was applied.

To generate instance-level and type-level annotations, a semi-automated human-in-the-loop pipeline was used, as shown in Fig. 2. Coarse nuclear masks were initially generated using HoverFast [32], a publicly available segmentation model that includes a variant pretrained for nuclear semantic segmentation on IHC-stained images. These preliminary masks were refined in QuPath through multiple rounds of split/merge editing and boundary correction. Trained scientists assigned preliminary subtypes; board-certified pathologists adjudicated ambiguities and harmonized criteria. Final annotations are released in standardized formats—OME-TIFF for images and mat for instance masks—to ensure reproducibility and compatibility with downstream pipelines.

#### *Statistics, Benchmark Characteristics, and Availability*

The final dataset comprises **18,483** quality-controlled nuclei assigned to **seven** histologically and biomarker-informed subtypes spanning CD47-positive/negative tumor, immune, stromal, and rare/other categories (Table 1; Fig. 2). The distribution exhibits a pronounced long tail: positive-tumor and immune nuclei dominate, whereas rare categories (e.g., negative-stroma and negative-others) are sparsely represented. Beyond class imbalance, the benchmark captures three key challenges: (i) stain heterogeneity (DAB intensity and counterstain variability), (ii) nuclear crowding and adhesion in tumor regions, and (iii) shape and size diversity across tissue contexts. Collectively, these factors stress a model's ability to jointly delineate instances and infer biomarker-sensitive types under real-world clinical heterogeneity.

Because three of the seven annotated categories are either extremely rare or carry limited clinical specificity—pOth (0.13%, 25 instances) and nOth (0.02%, 3 instances) represent morphologically ambiguous nuclei, while nSt (2.5%, 464 instances) provides insufficient test-set representation ($n_{\text{test}}$=54) for reliable per-class evaluation—they are merged into morphologically related categories before model training and evaluation: pOth is merged into pTu, and nSt is merged into nTu. The remaining negative-immune (nIm) and negative-others (nOth) categories are combined into a single **negative-others (nOth)** class, as both represent biomarker-negative non-tumor nuclei whose morphological distinction is un-

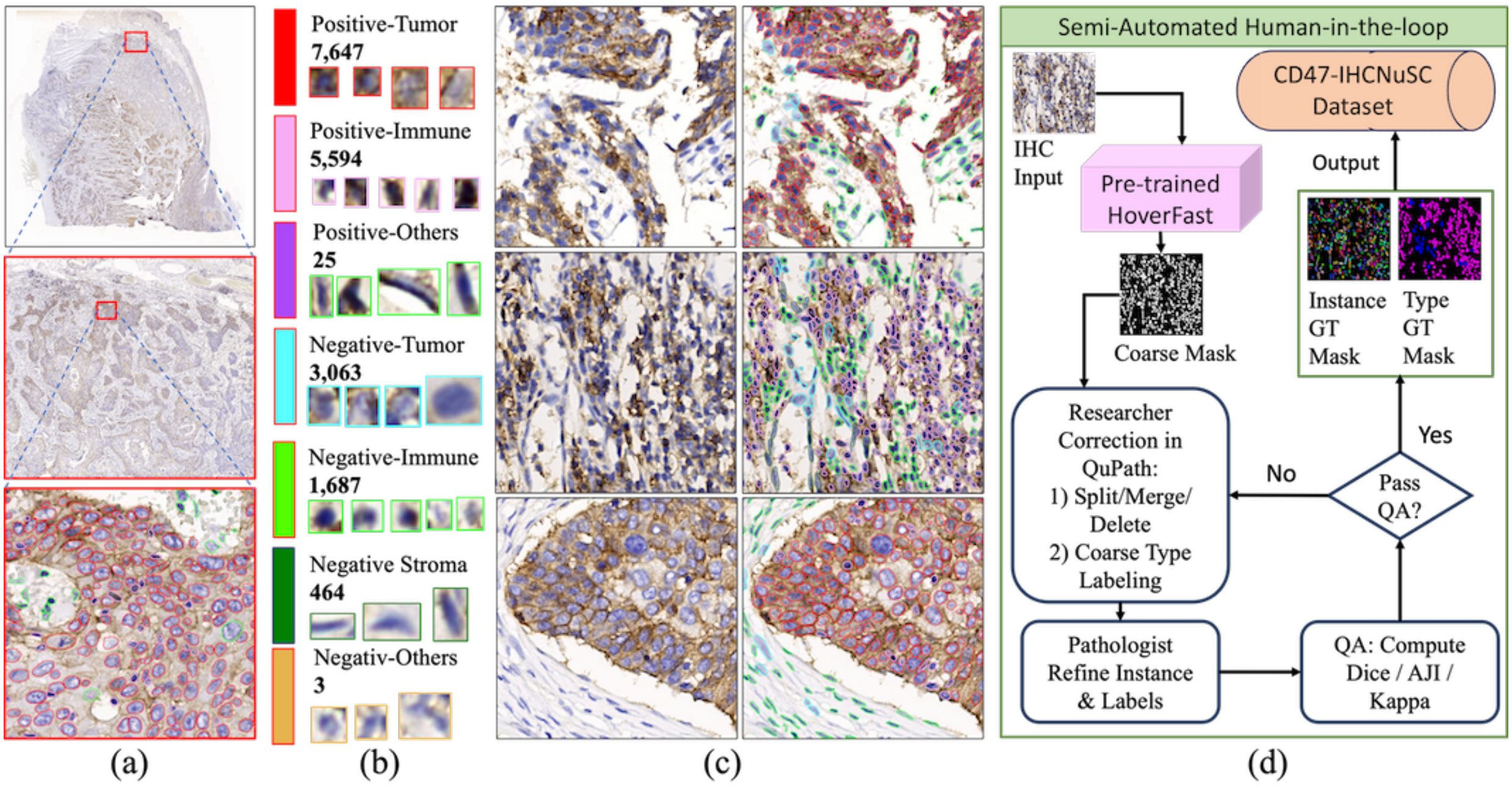


**Figure 2.** Overview of the CD47-IHCNuSC dataset and annotation workflow. (a) Representative CD47-stained WSIs, tumor-enriched regions, and extracted 1024 × 1024 patches. (b) Nuclear crops from seven annotated subtypes, including biomarker-positive and biomarker-negative tumor, immune, stromal, and rare cells; border colors correspond to subtype labels. (c) Visualization of subtype-labeled nuclei overlaid on the original image. Colors match subtype labels shown in (b). (d) Semi-automated annotation pipeline: HoverFast-based pre-labeling, manual refinement in QuPath, and expert validation with quality assurance.

reliable at the annotation granularity of this study. All models therefore operate on **four foreground classes** (pTu, pIm, nTu, nOth) plus background, and all evaluation is reported at this four-class granularity.

CD47-IHCNuSC complements the current breast-centric datasets [12, 23, 29, 30] and multiplex Tissue Microarray (TMA) corpora [13] by broadening biomarker and organ coverage to esophageal IHC with fine-grained nuclear subtyping. The combination of biomarker specificity, fine-grained nuclear categorization, real-world stain variability, and expert-reviewed annotations makes CD47-IHCNuSC a challenging and representative benchmark. It provides a valuable resource for training and evaluating segmentation models under clinically realistic IHC conditions. In the following section, we evaluate FPNuNet and existing baselines on CD47-IHCNuSC to characterize its utility and difficulty as a benchmark for biomarker-aware NuSC.

**Table 1.** Distribution of nuclear categories and image counts in the CD47-IHCNuSC dataset. *Abbrev.*: P = Positive, N = Negative, Tu = Tumor, Im = Immune, St = Stroma, Oth = Others.

| Nuclear Category | Abbrev. | Train | Test | Total | Proportion |
|---|---|---|---|---|---|
| Positive-Tumor | pTu | 5,958 | 1,689 | 7,647 | 40.5% |
| Positive-Immune | pIm | 4,712 | 882 | 5,594 | 29.6% |
| Positive-Others | pOth | 17 | 8 | 25 | 0.13% |
| Negative-Tumor | nTu | 1,473 | 214 | 1,687 | 8.9% |
| Negative-Immune | nIm | 2,631 | 432 | 3,063 | 16.2% |
| Negative-Stroma | nSt | 410 | 54 | 464 | 2.5% |
| Negative-Others | nOth | 3 | 0 | 3 | 0.02% |
| **Total Nuclei** | – | 15,204 | 3,279 | 18,483 | 100% |
| **Number of Images** | – | 70 | 16 | 86 | – |

## FPNuNet

As illustrated in Fig. 1, FPNuNet comprises three functional stages: 1) a multi-source feature extraction module with four parallel encoders for structural, semantic, spatial, and frequency-domain feature extraction, 2) DCT-enabled PFAE modules for multi-branch feature integration and spectral enhancement, and 3) multi-task decoders with hierarchical guidance for collaborative prediction. The four encoders consist of a frozen SAM ViT-B structural encoder and a frozen UNI ViT-L semantic encoder—both adapted through lightweight, input-dependent DCT-based prompt generators—together with WFE and MSCE branches, which extract complementary spatial and frequency-domain descriptors directly from the raw RGB input. All four feature streams are integrated within a four-way DCT-enabled PFAE global fusion neck. The resulting representations are decoded through three task-specific branches — binary, HV regression, and nuclear type classification — that jointly produce binary masks, horizontal–vertical offset vectors for instance separation, and nuclear subtype maps, supporting joint learning for NuSC. Of the 394.8 M total parameters, only 5.4 M (1.37%) are trainable, demonstrating parameter-efficient foundation-model adaptation for histopathology nuclei analysis.

### *Multi-Source Feature Extraction*

As shown in Fig. 1, the multi-source feature extraction stage comprises four parallel encoders that independently extract complementary multi-domain, multi-scale representations from the raw input image. Two frozen pretrained encoder backbones provide high-level structural and semantic features, while two lightweight multi-domain feature encoders extract spatial and frequency-domain descriptors directly from the RGB input.

A pretrained SAM Vision Transformer (ViT)-B image encoder [24] serves as the structural feature backbone. The encoder backbone is frozen except for a trainable patch embedding projection (Conv2d, 3 → 768, kernel 8 × 8, stride 8; 148,224 parameters) and a lightweight high-pass prompt generator (49,488 parameters). The prompt generator operates in two branches: a DCT-based high-pass branch that applies a frequency-domain mask to suppress low-frequency content and reconstruct edge-sensitive features from the input image, and a patch-embedding branch that

captures local structural cues. Both branches are fused through a shared bottleneck multi-layer perceptron (dimension 24) with per-block lightweight projections. For each of the 12 transformer blocks, the resulting token-wise prompt embedding is additively injected before the block, enabling deep, input-dependent visual prompt adaptation without modifying the pretrained attention weights. The SAM prompt encoder and mask decoder are not used; only the image encoder output at $16 \times 16$ spatial resolution is retained.

*Semantic Encoder (UNI).* A pretrained UNI ViT-L pathology foundation model [25] provides semantic embeddings. The UNI backbone is entirely frozen; domain adaptation is achieved through the same prompt generator architecture used for SAM, instantiated with a 1024-dimensional embedding space and a bottleneck dimension of 32 (103,872 parameters). Token-wise additive prompts are injected into 12 of UNI's 24 transformer layers (alternating layers 0, 2, …, 22), constituting deep visual prompt injection at half-depth granularity. Intermediate features from layers 3, 12, and 21 are extracted as skip features and reshaped to spatial resolutions of $32 \times 32$, $64 \times 64$, and $128 \times 128$ for decoder use.

Both encoders share the same `PromptGenerator` class. Unlike Low-Rank Adaptation [33], which applies input-agnostic weight perturbations, the prompt generator produces input-dependent, frequency-informed additive tokens, preserving the pretrained representation while injecting task-relevant boundary and texture cues.

*Multi-Domain Feature Encoders.* The pretrained encoder backbones provide high-level structural and semantic representations, but their outputs are spatially coarse after repeated downsampling, and their feature statistics carry pretrained-domain bias that may not fully preserve the high-frequency textures and local contrast critical for nuclear boundary delineation in IHC images. To compensate, FPNuNet introduces two lightweight multi-domain feature encoders that operate directly on the raw RGB input, supplying complementary frequency-domain and spatial-domain descriptors that are orthogonal to the pretrained features.

As shown in Fig. 3(a), WFE decomposes the input via Haar wavelet pooling convolution into four sub-bands—LL (low-frequency approximation), LH, HL, and HH (horizontal, vertical, and diagonal high-frequency detail)—which are concatenated along the channel dimension, upsampled, and fused back to the input resolution through a $3 \times 3$ convolution followed by a $1 \times 1$ projection (952 trainable parameters), producing a single full-resolution frequency descriptor $\mathbf{F}_{\mathrm{WFE}} \in \mathbb{R}^{8\times128\times128}$. Wavelet decomposition inherently supports multi-resolution analysis: it separates frequency components while preserving spatial localization, enabling the model to perceive nuclear boundaries, chromogen deposition textures, and other high-frequency structural cues that are often obscured by the stain heterogeneity and weak boundary contrast characteristic of IHC images.

As shown in Fig. 3(b), MSCE extracts multi-granularity spatial context through three parallel branches. Each branch applies a $3 \times 3$ convolution at a different spatial scale—original, 1/2, and 1/4 resolution—to capture features ranging from fine-grained local textures to broader regional morphology. The branch outputs are upsampled to the original resolution, concatenated, and fused via a $1 \times 1$ projection (7,384 trainable parameters), yielding a single full-resolution spatial descriptor $\mathbf{F}_{\mathrm{MSCE}} \in \mathbb{R}^{8\times128\times128}$. Together with $\mathbf{F}_{\mathrm{WFE}}$, these descriptors compensate for the fine-grained spatial detail that the pretrained backbones lose through downsampling.

The four encoders run in parallel, each independently extracting features from the raw input image. The pretrained backbones output structural features $\mathbf{F}_{\mathrm{SAM}}$ and semantic features $\mathbf{F}_{\mathrm{UNI}}$; the multi-domain encoders output $\mathbf{F}_{\mathrm{WFE}}$ and $\mathbf{F}_{\mathrm{MSCE}}$. All four feature streams are forwarded to the PFAE-based global fusion neck for alignment and integration.

***Frequency-Aware Fusion Modules***

FPNuNet employs DCT-based frequency-domain processing at two distinct architectural stages. At the *encoder adaptation* stage, DCT-based high-pass filtering within the prompt generators extracts boundary and texture cues from the raw input image, compensating for the low-pass effect of ViT patch tokenization. At the *post-encoder fusion* stage, DCT-enabled PFAE modules refine learned feature representations in the frequency domain. DCT is adopted uniformly at both stages because it produces purely real-valued coefficients and concentrates spectral energy of smooth, localized structures—characteristic of IHC nuclear textures—into fewer low-frequency components, providing a compact and efficient spectral basis for both input-level prompt generation and feature-level enhancement. This uniform transform choice also eliminates the complex-number bookkeeping (magnitude/phase storage, complex-to-real conversion) that the fast Fourier transform (FFT) would impose, simplifying the gradient path through both stages. Empirical comparison of transform configurations confirms that the DCT-only design achieves the best overall performance.

The fusion stage comprises a PFAE-based global fusion neck that integrates encoded features from both encoders together with complementary raw-image features, and two PFAE-based skip enhancement necks that refine shallow features from the semantic encoder. The following subsections detail the PFAE block architecture and the global fusion neck design.

*PFAE Block.* Although many segmentation models directly use encoder outputs and skip connection features for decoding, they often fail to preserve high-frequency structural cues essential for accurate nuclear boundary delineation, particularly under stain variability and morphological ambiguity. To address this, we introduce the PFAE block, which combines multi-scale spatial context, DCT-based frequency-domain attention, and positional priors in a residual refinement pipeline.

The overall architecture of the PFAE block is illustrated in Fig. 4(a). Let $\mathbf{X} \in \mathbb{R}^{C\times H\times W}$ denote the input feature map. The input undergoes an initial $1 \times 1$ projection:

$$\mathbf{F}_0 = \phi_{\mathrm{proj}}(\mathbf{X}),$$

producing a compact embedding $\mathbf{F}_0 \in \mathbb{R}^{C\times H\times W}$ that anchors three progressive refinement stages. At each stage $s = 1, 2, 3$, a dilated convolution $\phi_{DC_k}(\cdot)$ with kernel size $3 \times 3$ and dilation rate $k \in \{3, 5, 7\}$ increases the receptive field, followed by a lightweight frequency-aware attention module $\psi_{\mathrm{LWFA}}(\cdot)$:

$$\mathbf{S}_s = \phi_{DC_k}(\mathbf{F}_{s-1}), \tag{1}$$

$$\mathbf{F}_s = \mathbf{S}_s + \psi_{\mathrm{LWFA}}(\mathbf{S}_s). \tag{2}$$

Each $\psi_{\mathrm{LWFA}}(\cdot)$ transforms the spatial feature $\mathbf{S}_s$ into the frequency domain via a real-valued DCT—chosen for the energy-compaction and real-valued coefficient properties discussed above—and applies multi-head attention directly on the resulting spectral embedding to focus on morphology-relevant frequency bands. A Convolutional Block Attention Module (CBAM) [34] further refines frequency responses through sequential channel and spatial attention, as shown in Fig. 4(b).

To aggregate multi-scale outputs, a global context vector $\mathbf{G}$ is extracted from $\mathbf{F}_0$ via average pooling and fused with the outputs $\{\mathbf{F}_1, \mathbf{F}_2, \mathbf{F}_3\}$ and the initial embedding $\mathbf{F}_0$ through softmax-normalized attention weights $\{\alpha_k\}$:

$$\mathbf{F} = \mathrm{Concat}\left[\alpha_k \cdot \mathbf{O}_k\right]_{k=0}^{4}, \quad \mathbf{O} = \{\mathbf{F}_0, \mathbf{F}_1, \mathbf{F}_2, \mathbf{F}_3, \mathbf{G}\} \tag{3}$$

Finally, coordinate-aware gating injects spatial priors, as shown

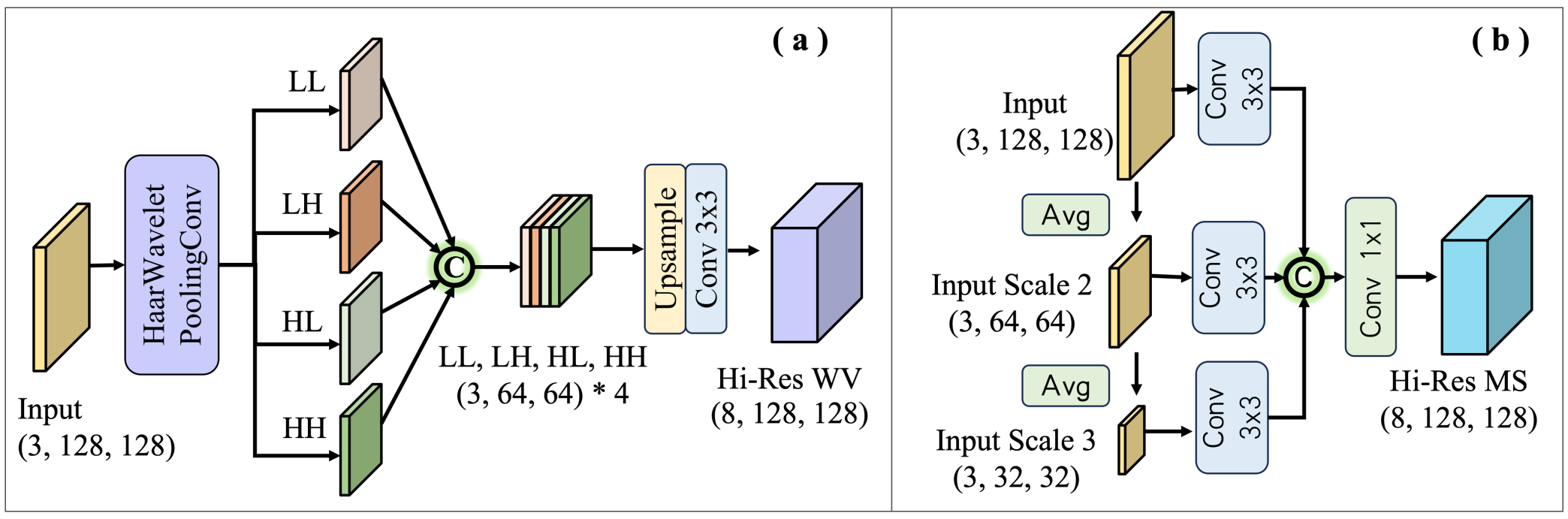


**Figure 3.** Multi-domain feature encoders. (a) WFE: Haar wavelet decomposition into LL/LH/HL/HH sub-bands, concatenation, and upsampled fusion. (b) MSCE: three-scale parallel convolution with upsampled fusion.

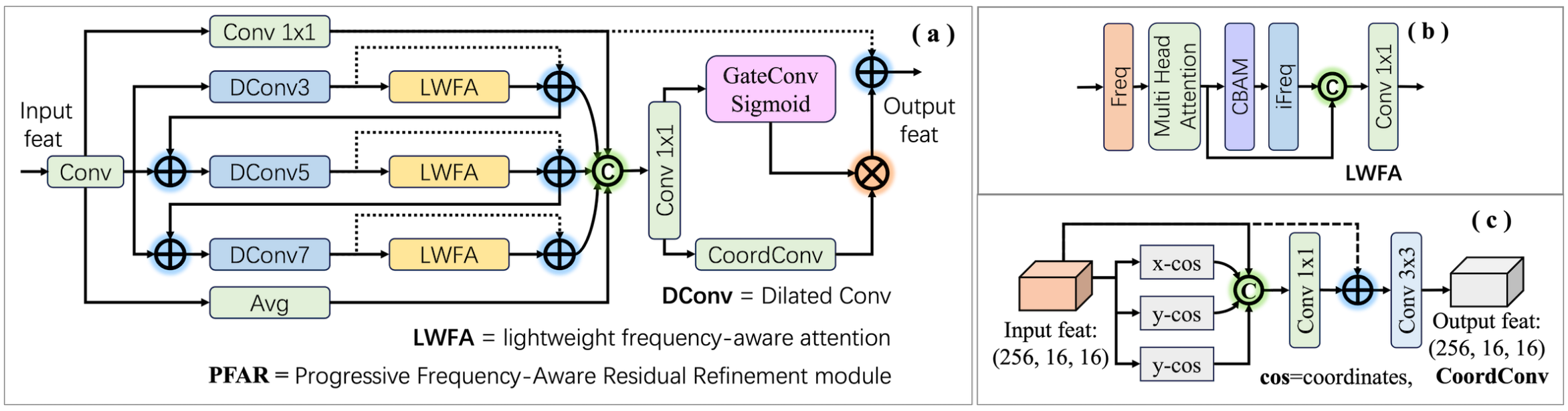


**Figure 4.** PFAE block architecture. (a) Overview: three progressive refinement stages with dilated convolutions, DCT-based lightweight frequency-aware attention (LWFA), CBAM, multi-scale aggregation, and coordinate-aware gating. (b) LWFA module. (c) Coordinate-aware gating.

in Fig. 4(c):

$$\mathbf{Y} = \phi(\mathbf{F}) + \delta_{\text{coord}}(\tilde{\mathbf{F}}) \odot \sigma(\phi_{\text{gate}}(\tilde{\mathbf{F}})), \quad (4)$$

where $\tilde{\mathbf{F}}$ is a $1 \times 1$ convolution of $\mathbf{F}$, $\delta_{\text{coord}}(\cdot)$ injects coordinate priors through trigonometric position encodings, $\sigma(\cdot)$ is the sigmoid activation, and $\odot$ denotes element-wise multiplication.

*PFAE-Based Global Fusion Neck.* The PFAE-based global fusion neck (Fig. 5) receives the four feature streams produced by the multi-source extraction stage: $\mathbf{F}_{\text{SAM}} \in \mathbb{R}^{768\times16\times16}$, $\mathbf{F}_{\text{UNI}} \in \mathbb{R}^{1024\times16\times16}$, $\mathbf{F}_{\text{WFE}} \in \mathbb{R}^{8\times128\times128}$, and $\mathbf{F}_{\text{MSCE}} \in \mathbb{R}^{8\times128\times128}$.

To align spatial resolutions, the full-resolution WFE and MSCE features are pooled and projected to match the encoder feature resolution. The four projected streams are concatenated and fused via a $1 \times 1$ convolution:

$$\mathbf{F}_{\text{cat}} = \phi_{\text{fuse}}(\text{Concat}[\phi_s(\mathbf{F}_{\text{SAM}}), \phi_u(\mathbf{F}_{\text{UNI}}), \tilde{\mathbf{F}}_{\text{WFE}}, \tilde{\mathbf{F}}_{\text{MSCE}}]),$$

where $\tilde{\mathbf{F}}_{\text{WFE}}$ and $\tilde{\mathbf{F}}_{\text{MSCE}}$ denote the pooled and projected WFE and MSCE features at the encoder resolution. The fused representation is refined by a DCT-enabled PFAE block with a multi-head attention stage (4 heads), yielding a spectrally enhanced representation $\mathbf{F}_{\text{pfae}}$. To inject high-resolution spatial detail, the full-resolution WFE and MSCE features are concatenated, downsampled, and passed through a gating mechanism:

$$\mathbf{F}_{\text{out}} = \text{Pos}(\mathbf{F}_{\text{cat}} + \mathbf{F}_{\text{pfae}} + \sigma(\mathbf{g}) \odot \mathbf{v}),$$

where $\mathbf{g}$ and $\mathbf{v}$ are the gate and value projections of the concatenated high-resolution features, $\sigma(\cdot)$ is the sigmoid activation, and $\text{Pos}(\cdot)$ applies position-aware enhancement. The output $\mathbf{F}_{\text{out}} \in \mathbb{R}^{256\times16\times16}$ is forwarded to all three decoder branches.

In addition, two DCT-enabled PFAE skip enhancement necks (7,246 parameters total) refine UNI skip features at two spatial levels ($32 \times 32$ and $64 \times 64$), strengthening shallow feature propagation to the decoders. The fusion neck accounts for 2,634,507 trainable parameters.

### Multi-Task Decoders with Hierarchical Guidance

Accurate nuclear quantification necessitates the concurrent resolution of three interrelated tasks: binary segmentation to identify nuclear regions, instance separation to delineate individual cells, and phenotype classification to assign biologically meaningful subtypes. FPNuNet adopts a sequential, causally guided multi-task decoder inspired by HoVer-Net [7], consisting of three task-specific branches—binary, HV, and type decoders. The binary decoder executes first and produces multi-scale intermediate feature maps; these features (not final logits) are then propagated to both the HV and type decoders through sigmoid-gated fusion modules, reinforcing nuclear localization and cross-task consistency at the feature level. All branches receive multi-scale, DCT-enhanced features from the PFAE-based fusion neck and skip pathways, ensuring that both structural fidelity and semantic richness are preserved throughout decoding.

*Binary Segmentation Decoder.* The binary decoder (578,628 parameters; Fig. 6(b)) serves as the structural foundation for both instance separation and phenotype classification. It adopts a three-stage hierarchical design where, at each stage, fused input features are upsampled via pixel-shuffle blocks ($1 \times 1$ convolution followed by PixelShuffle(2), batch normalization, and ReLU) and concatenated with DCT-enhanced skip features from the UNI encoder. The concatenated feature map is processed by a convolutional refinement block to produce resolution-aligned feature maps. The decoder outputs binary nuclei logits and boundary logits. Critically, it also outputs multi-scale intermediate feature maps that provide prior guidance for the downstream HV and type decoders.

The binary branch is supervised by a composite loss:

$$\mathcal{L}_{\text{bin}} = \mathcal{L}_{\text{BCE}} + \mathcal{L}_{\text{Dice}} + \mathcal{L}_{\text{Focal}} + 0.5 \cdot \mathcal{L}_{\text{boundary}}, \quad (5)$$

where $\mathcal{L}_{\text{boundary}}$ supervises the predicted boundary map to sharpen nuclear contour delineation, addressing class imbalance and pro-

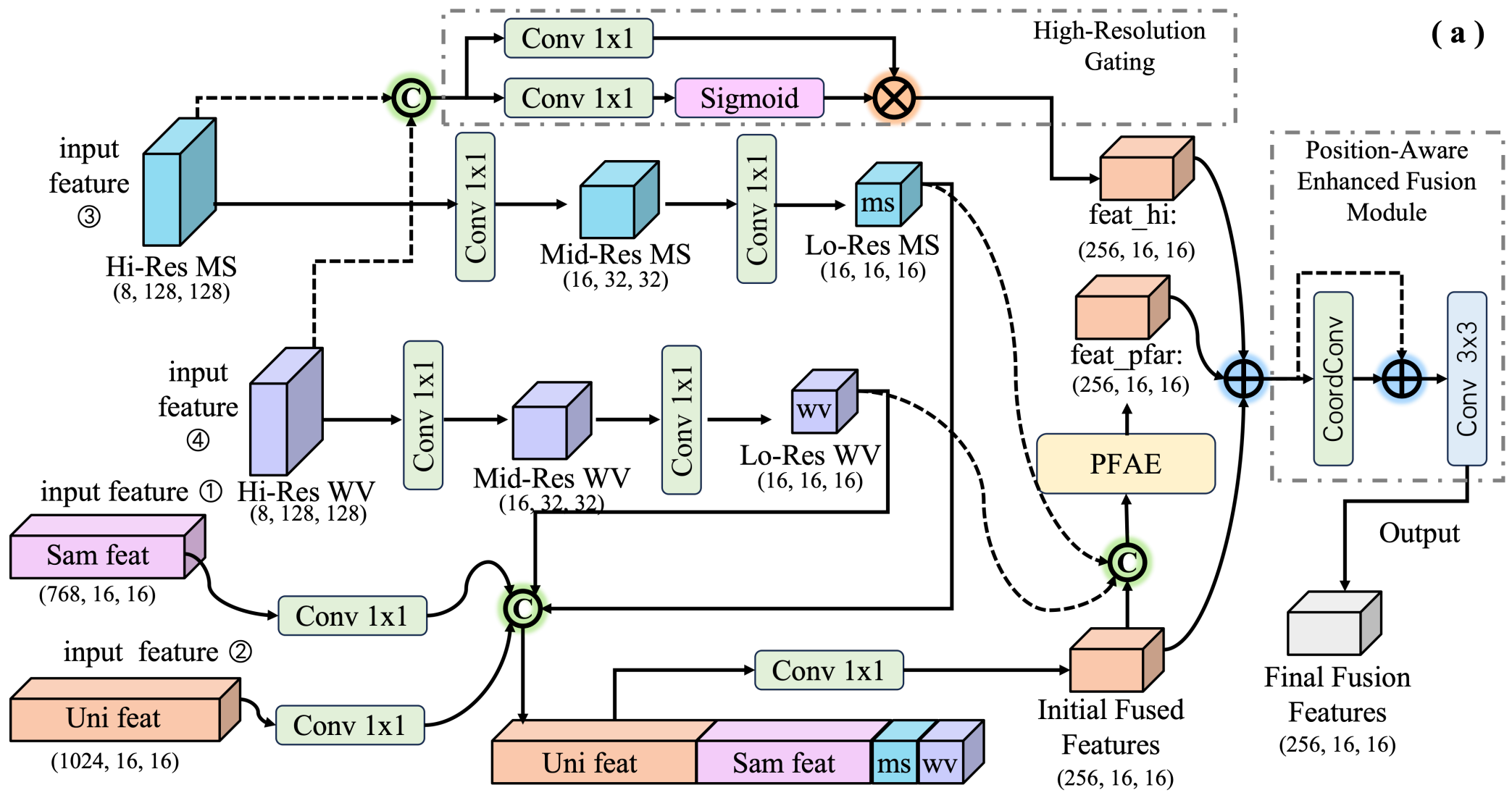


**Figure 5.** PFAE-based global fusion neck. The neck receives SAM, UNI, WFE, and MSCE features, aligns them at the encoder resolution via channel projection and concatenation, refines the fused representation with a DCT-enabled PFAE block, and injects full-resolution WFE/MSCE detail through a high-resolution sigmoid gate followed by position-aware enhanced fusion.

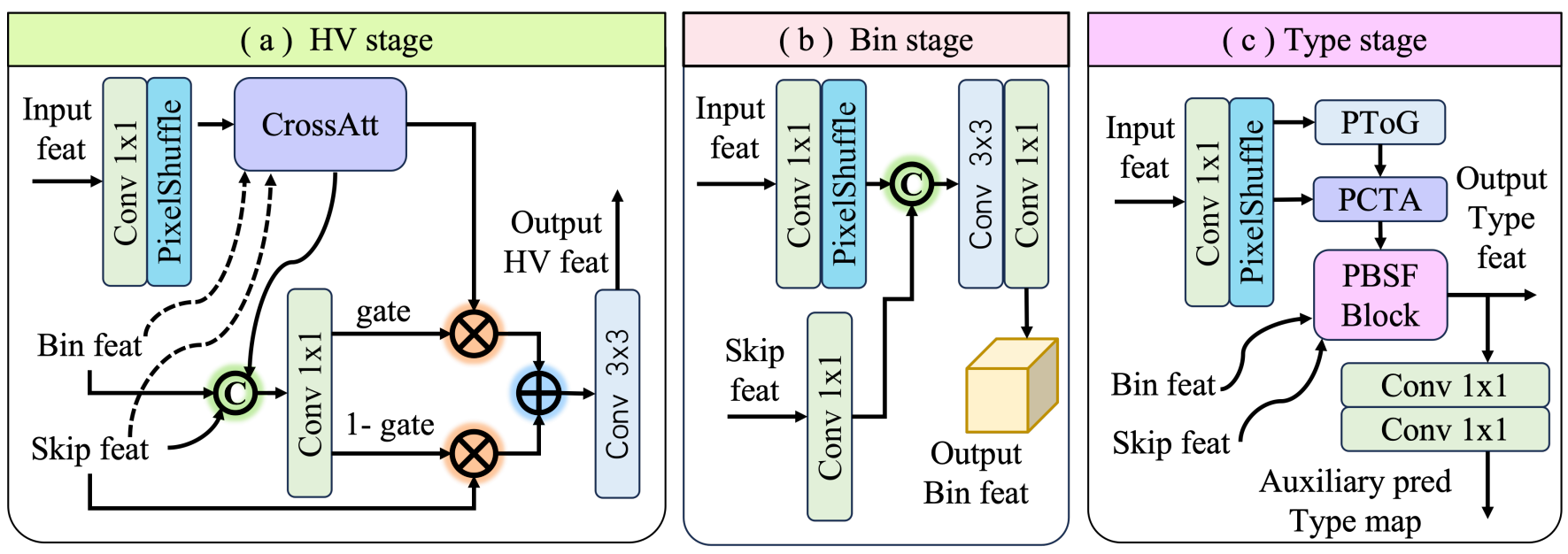


**Figure 6.** Collaborative multi-task decoder with hierarchical binary guidance. (a) HV vector regression decoder (binary-guided, bilinear upsampling, dense refinement). (b) Binary segmentation decoder (produces logits and intermediate feature guidance maps). (c) Nuclear type classification decoder (prompt-to-feature cross-attention, binary-guided skip fusion).

viding reliable structural priors for the other branches.

*HV Vector Regression Decoder.* The HV decoder (763,718 parameters; Fig. 6(a)) estimates pixel-wise horizontal and vertical offsets $\hat{\mathbf{V}} \in \mathbb{R}^{2\times H\times W}$ pointing from each pixel to its corresponding nuclear centroid for instance-level separation. The HV decoder employs bilinear upsampling blocks (replacing pixel-shuffle), dense feature refinement blocks for local detail recovery, and a unified binary-guided skip fusion module at each stage. Each fusion module applies cross-attention with decoder features as queries and UNI skip features as keys and values, followed by a sigmoid-gated modulation using binary decoder intermediate features. This mechanism allows binary segmentation priors to adaptively control how skip features enter the HV prediction path.

Supervision adopts foreground-masked regression:

$$\mathcal{L}_{\text{hv}} = \mathcal{L}_{\text{MSE}}^{\text{fg}} + \mathcal{L}_{\text{MSGE}}^{\text{full-grad}}, \tag{6}$$

where $\mathcal{L}_{\text{MSE}}^{\text{fg}}$ applies mean squared error only within the foreground mask, and $\mathcal{L}_{\text{MSGE}}^{\text{full-grad}}$ denotes the full-gradient mean squared gradient error computed from Sobel derivatives, promoting smooth and spatially coherent HV maps. Both terms carry equal weight (1.0).

*Nuclear Type Classification Decoder.* The type decoder (1,104,219 parameters; Fig. 6(c)) classifies each pixel into one of $K$ nuclear subtypes through a dedicated prompt-to-feature cross-attention mechanism.

At each of the three decoding stages, the decoder receives the same tri-source input as the HV decoder: upsampled features from the previous stage, DCT-enhanced UNI skip features, and binary decoder intermediate feature maps. To introduce global semantic priors, a prompt token generator produces learnable type-specific tokens. In contrast to earlier decoder versions where spatial features queried prompt tokens, the type decoder reverses the attention direction: *learned prompt tokens serve as Query, while pooled decoder features serve as Key and Value* in multi-head cross-attention. This design enables each type prompt to actively retrieve image-specific evidence from the entire spatial feature map, producing a prompt-conditioned global vector that is projected back to the feature dimension and added residually to the decoder features. Subsequently, binary-guided skip fusion (identical to the HV decoder) modulates the contribution of structural and semantic information through sigmoid-gated feature integration.

The type branch is supervised by a multi-term classification loss with equal weighting (1.0) for all four terms:

$$\mathcal{L}_{\text{type}} = \mathcal{L}_{\text{CE}}^{w} + \mathcal{L}_{\text{Dice}} + \mathcal{L}_{\text{Focal}} + \mathcal{L}_{\text{IoU}}, \tag{7}$$

where $\mathcal{L}_{\text{CE}}^{w}$ is class-weighted cross-entropy in which background is down-weighted (weight 0.25) relative to all foreground classes (weight 1.0), and $\mathcal{L}_{\text{IoU}}$ is a soft IoU loss. The focal term uses a focusing parameter of $\gamma$=2.0. As described in Section Data Description, rare and morphologically ambiguous categories are merged before training, yielding **four foreground classes** (pTu, pIm, nTu, nOth) plus background.

**Table 2.** Model complexity comparison. FLOPs are reported as 2× multiply–accumulate operations on a single NVIDIA RTX 4090 GPU; profiled with THOP. StarDist is omitted owing to its TensorFlow backend (incompatible with THOP); SRSA-Net is omitted because the publicly released code does not expose a standalone model class suitable for THOP profiling.

| Model | Input | Total Params | Trainable Params | FLOPs |
|---|---|---|---|---|
| HoverFast | $256^2$ | 0.95 M | 0.95 M (100%) | 33.2 G |
| HoVer-Net | $270^2$ | 54.7 M | 54.7 M (100%) | 394.1 G |
| Cellpose-SAM | $256^2$ | 304.6 M | 304.6 M (100%) | 621.1 G |
| **FPNuNet** | $128^2$ | 394.8 M | **5.4 M (1.37%)** | 111.2 G |

*Overall Loss.* The overall training objective combines all three branch losses with equal weight:

$$\mathcal{L}_{\text{total}} = \mathcal{L}_{\text{bin}} + \mathcal{L}_{\text{hv}} + \mathcal{L}_{\text{type}} \tag{8}$$

At inference, individual nuclear instances are extracted from the predicted binary mask and HV map using the same Sobel-gradient-based watershed post-processing as HoVer-Net [7], and each instance is assigned the majority predicted type from the type map.

# Results

## Experimental Setup

We evaluated FPNuNet on the curated CD47-IHCNuSC dataset, processed into overlapping 128 × 128 patches (stride 64) with augmentation via flips, rotations, color jittering, and stain perturbation. FPNuNet was trained with the AdamW optimizer (batch size 8, weight decay $1 \times 10^{-2}$) using bf16 mixed precision, adopting a dual-rate learning schedule: $1 \times 10^{-3}$ for prompt generator parameters and $5 \times 10^{-4}$ for all other trainable components. Both learning-rate groups followed a step-level schedule with linear warmup over 1,000 steps and multiplicative decay by 0.5 at steps 20,000 and 27,000, for a total of 30,000 optimizer steps (∼12 epochs). The pretrained SAM (natural images) and UNI (pathology images) encoders provided initial representations, with prompt generators fine-tuned jointly. All experiments were conducted using PyTorch 2.0 and MONAI on a workstation equipped with four NVIDIA RTX 4090 GPUs.

*Model complexity.* FPNuNet contains 394.8 M total parameters, of which only 5.4 M (1.37%) are trainable. The majority of parameters reside in the frozen UNI backbone (303.4 M) and the frozen SAM backbone (86.1 M). Among trainable components, the fusion neck is the largest (2.6 M), followed by the type decoder (1.1 M), HV decoder (0.8 M), binary decoder (0.6 M), and SAM/UNI prompt generators (0.2 M combined). Table 2 compares model complexity for all baselines amenable to THOP-based profiling. Despite having the largest total parameter count, FPNuNet has the smallest trainable footprint (5.4 M, 1.37%)—over 10× fewer trainable parameters than HoVer-Net (54.7 M) and over 56× fewer than Cellpose-SAM (304.6 M). Its FLOPs (111.2 G) are likewise lower than those of HoVer-Net (394.1 G) and Cellpose-SAM (621.1 G). On a single NVIDIA RTX 4090 GPU with a 128×128 input, the sequential four-encoder forward pass averages 106.7 ms latency and 1.62 GB peak memory, higher than single-backbone baselines (e.g., HoVer-Net: 13.6 ms) owing to the serial execution of two frozen ViT encoders.

FPNuNet is compared against five baseline methods: **HoVer-Net** [7], **SRSA-Net** [13], **StarDist** [35], **HoverFast** [32], and **Cellpose-SAM** [36]. To evaluate the contribution of key components, several FPNuNet variants are included in the ablation study: **FPNuNet-F** (dual encoders SAM+UNI with FFT-based frequency-aware enhancement, concatenation fusion neck, standard binary/HV/type decoders, no prompt adapter on UNI), **FPNuNet-A** (the baseline augmented with symmetric prompt generators on both encoders for cross-domain feature alignment), and **FPNuNet-AP** (the adapter variant further augmented with a prompt-guided type decoder that injects learnable subtype tokens via cross-attention into the type branch). The full **FPNuNet** model additionally replaces the FFT-based prompt generators with DCT-based variants, upgrades the fusion and skip necks to DCT-enabled PFAE, upgrades the HV and type decoder architectures, and adds skip-connection enhancement necks.

For a controlled comparison, the FPNuNet series, HoVer-Net, SRSA-Net, HoverFast, and Cellpose-SAM were trained on the same CD47-IHCNuSC training split using consistent training and evaluation protocols whenever applicable. Since StarDist, HoverFast, and Cellpose-SAM are instance-segmentation–oriented methods, they were included in the instance-level segmentation comparisons but not in subtype classification evaluation. StarDist was evaluated with its publicly released pretrained model as an off-the-shelf instance segmentation baseline.

## Evaluation and Metrics

The performance of nuclei analysis models is assessed along two complementary axes: 1) nuclear instance segmentation, which evaluates the recovery of object identities and boundaries; and 2) nuclear classification, which covers both dense, pixel-wise semantic segmentation (treated as per-pixel classification) and instance-level subtype recognition.

### *Nuclear Instance Segmentation Evaluation*

Nuclear instance segmentation is quantified using complementary criteria that assess how well a method detects, separates, and delineates individual instances. Given a set of ground-truth (GT) nuclear masks $\mathcal{G}$ and a set of predicted nuclear masks $\mathcal{S}$ for an image, region-overlap metrics such as the Intersection-over-Union (IoU) and Dice coefficient (**Dice**) [37] measure pixel-wise agreement between the corresponding regions:

$$\text{IoU} = \frac{|G \cap S|}{|G \cup S|}, \tag{9}$$

$$\text{Dice} = \frac{2\,|G \cap S|}{|G| + |S|}. \tag{10}$$

The pairwise aggregate variant (**Dice2**) [38] computes Dice over all matched ground-truth–prediction pairs and averages them, providing a more instance-sensitive estimate of segmentation accuracy.

To further incorporate unmatched regions, the Aggregated Jaccard Index (AJI) [39] aggregates intersections and unions over all matched pairs, penalizing unpaired ground-truth or prediction regions:

$$\text{AJI} = \frac{\sum |G \cap S|}{\sum |G \cup S| + \sum |G_{\text{unmatched}}| + \sum |S_{\text{unmatched}}|}. \tag{11}$$

Its assignment-robust variant AJI+ replaces greedy matching with optimal bipartite (Hungarian) assignment to better handle clumped or overlapping nuclei [7].

Following the HoVer-Net [7] and SRSA-Net evaluation protocol [13], we report Panoptic Quality (PQ) [40] together with its decomposition into Detection Quality (DQ) and Segmentation Quality (SQ). Binary PQ (bPQ) is computed across all nuclei regardless of type, while the type-aware counterpart evaluates per-class panoptic quality. Let TP (true positives), FP (false positives), and FN (false negatives) represent correctly matched nuclei, unmatched predictions, and missed GT nuclei, respectively. Then DQ and SQ

are defined as:

$$\mathrm{DQ} = \frac{\mathrm{TP}}{\mathrm{TP} + 0.5\,\mathrm{FP} + 0.5\,\mathrm{FN}}, \tag{12}$$

$$\mathrm{SQ} = \frac{1}{\mathrm{TP}} \sum \mathrm{IoU}(G, S), \tag{13}$$

and the overall PQ is obtained as:

$$\mathrm{PQ} = \mathrm{DQ} \times \mathrm{SQ}. \tag{14}$$

In addition, we evaluate model performance using Precision ($\mathbf{P}$), Recall ($\mathbf{R}$), and F1-score ($\mathbf{F}_1$), which are mathematically defined as:

$$\mathbf{P} = \frac{\mathrm{TP}}{\mathrm{TP} + \mathrm{FP}}, \quad \mathbf{R} = \frac{\mathrm{TP}}{\mathrm{TP} + \mathrm{FN}}, \quad \mathbf{F}_1 = \frac{2\,\mathbf{P}\,\mathbf{R}}{\mathbf{P} + \mathbf{R}}. \tag{15}$$

To distinguish these from pixel-level measures, we denote the object-level Precision ($\mathbf{P}_{\mathrm{obj}}$), Recall ($\mathbf{R}_{\mathrm{obj}}$), and F1-score ($\mathbf{F}_{1,\mathrm{obj}}$), computed from nucleus-level TP, FP, and FN counts. Together, these metrics cover pixel-wise overlap, instance separation, boundary accuracy, and assignment robustness.

***Nuclear Classification Evaluation***

Nuclear type classification is evaluated using instance-level metrics that assess the correctness of subtype labels assigned to individual nuclei while accounting for detection performance. Given the GT set with type annotations $\mathcal{G} = \{(g_i, y_i)\}_{i=1}^{N}$ and the predicted set with assigned types $\mathcal{S} = \{(s_j, \hat{y}_j)\}_{j=1}^{M}$, a one-to-one correspondence $\mathcal{M} \subseteq \mathcal{G} \times \mathcal{S}$ is established by matching predicted and GT nuclei. Each matched prediction is then assigned the class with the highest predicted score. Background (class 0) is excluded.

For each matched pair $(g_i, s_j)$, a TP is counted if $\hat{y}_j = y_i$. A label mismatch contributes a FP for the predicted class $\hat{y}_j$ and a FN for the GT class $y_i$. Unmatched predictions and unmatched GT instances are counted as FP and FN, respectively. This matching procedure yields the per-class counts $\{TP^{(k)}, FP^{(k)}, FN^{(k)}\}_{k=1}^{K}$, which form the basis for the macro-, micro-, and support-weighted $\mathrm{F}_1$ scores, as well as accuracy variants outlined below.

For each class $k \in \{\mathrm{pTu}, \mathrm{pIm}, \mathrm{nTu}, \mathrm{nOth}\}$ (excluding background), the instance-level Precision ($\mathrm{P}_{\mathrm{inst}}^{(k)}$), Recall ($\mathrm{R}_{\mathrm{inst}}^{(k)}$), and F1-score ($\mathrm{F}_{1,\mathrm{inst}}^{(k)}$) are calculated as follows:

$$\mathrm{P}_{\mathrm{inst}}^{(k)} = \frac{TP^{(k)}}{TP^{(k)} + FP^{(k)}}, \quad \mathrm{R}_{\mathrm{inst}}^{(k)} = \frac{TP^{(k)}}{TP^{(k)} + FN^{(k)}}, \tag{16}$$

$$\mathrm{F}_{1,\mathrm{inst}}^{(k)} = \frac{2\,\mathrm{P}_{\mathrm{inst}}^{(k)}\,\mathrm{R}_{\mathrm{inst}}^{(k)}}{\mathrm{P}_{\mathrm{inst}}^{(k)} + \mathrm{R}_{\mathrm{inst}}^{(k)}}. \tag{17}$$

The macro-averaged scores $\mathbf{P}_{\mathrm{cls,\,macro}}$ and $\mathbf{F}_{1,\mathrm{macro}}$ are obtained by averaging the per-class instance-level precision and $\mathrm{F}_1$ with equal weights. The support-weighted metrics $\mathbf{P}_{\mathrm{w}}$ and $\mathbf{F}_{1,\mathrm{w}}$ weight per-class scores by class support, with $w_k = n^{(k)}/N$ where $n^{(k)}$ is the number of GT nuclei in class $k$ and $N = \sum_k n^{(k)}$. The micro-averaged $\mathrm{F}_1$ score $\mathbf{F}_{1,\mathrm{micro}}$ is computed from the TP, FP, and FN counts aggregated across all classes.

For further evaluation, the overall metrics include the mean Panoptic Quality (mPQ) over non-background classes, computed as $\mathrm{mPQ} = \frac{1}{K}\sum_{k=1}^{K} \mathrm{PQ}^{(k)}$, where $\mathrm{PQ}^{(k)} = \mathrm{DQ}^{(k)} \times \mathrm{SQ}^{(k)}$ as defined in the instance segmentation subsection. A match is counted as $TP^{(k)}$ only when the predicted type equals the ground-truth type $k$ (type-consistent PQ).

## Nuclear Instance Segmentation Performance

FPNuNet achieves the strongest overall instance-segmentation performance on CD47-IHCNuSC across region-overlap, boundary, and detection metrics (Table 3; Figs. 7 and 8). The box plots in Fig. 8 reveal higher medians and tighter interquartile ranges for AJI, AJI+, and bPQ, indicating stable gains across individual test images rather than improvements confined to a small subset. Qualitatively, FPNuNet produces cleaner splits in crowded clusters, smoother yet well-aligned contours along membrane staining, and fewer missed small nuclei than competing methods (Fig. 7).

To quantify the statistical reliability of observed differences given the limited test set ($N$=16), we conducted paired per-image bootstrap 95% confidence interval estimation (10,000 resamples) and two-sided Wilcoxon signed-rank tests with Holm–Bonferroni correction for multiple comparisons. On AJI, FPNuNet yields higher values than every compared method and ablation variant (corrected $p < 0.05$), with the largest margins observed over StarDist (+0.299, 95% CI [0.257, 0.344]) and HoverFast (+0.235, 95% CI [0.208, 0.261]). On bPQ and SQ, FPNuNet yields higher values than all methods except HoVer-Net, for which the observed differences are small (bPQ: +0.010, $p$ = 0.28; SQ: −0.001, $p$ = 0.50). Conversely, on Dice2, FPNuNet scores lower than HoVer-Net (−0.028, 95% CI [−0.040, −0.017]) and Cellpose-SAM (−0.050, 95% CI [−0.068, −0.032]), suggesting that this pairwise overlap measure favours methods with dedicated semantic segmentation heads. On DQ, FPNuNet yields higher values than SRSA-Net (+0.148), HoverFast (+0.207), StarDist (+0.382), and FPNuNet-F (+0.034; corrected $p < 0.01$), whereas the smaller margins over HoVer-Net (+0.013, $p$ = 0.53), Cellpose-SAM (+0.001, $p$ = 0.63), FPNuNet-A, and FPNuNet-AP remain above the corrected threshold. On Dice, FPNuNet scores 0.008 lower than HoVer-Net (95% CI [−0.011, −0.005]) and higher than every other compared method and ablation variant, including Cellpose-SAM (+0.060, 95% CI [0.044, 0.080]; corrected $p < 0.001$). Within the ablation family, all three partial configurations (FPNuNet-F, -A, -AP) score below the full model on AJI, bPQ, and SQ (corrected $p < 0.05$), consistent with complementary contributions from the added modules.

Ablation across the FPNuNet family reveals progressive and complementary contributions from each module. Introducing adapters (FPNuNet-F → FPNuNet-A) yields the largest detection-quality gain in the ablation sequence (+2.22 percentage points (pp) DQ; Table 4), with concurrent improvements across all overlap metrics, suggesting that adapters primarily improve instance recovery and contact handling under weak IHC signal. Adding the prompt-guided decoder (FPNuNet-AP) further strengthens boundary quality (+0.96 pp bPQ) and shape fidelity (+1.26 pp SQ), while detection quality is essentially maintained. The complete FPNuNet configuration produces the largest single-step gain, with broad-based improvements led by bPQ (+3.44 pp) and SQ (+2.41 pp), accompanied by higher $\mathbf{P}_{\mathrm{inst}}$ at negligible cost to $\mathbf{R}_{\mathrm{inst}}$ (Table 4). These trends are corroborated by the upward median shifts and narrower interquartile ranges in Fig. 8, as well as the qualitative overlays in Fig. 7: adapters reduce missed detections at low-contrast boundaries, the prompt-guided decoder suppresses residual merges in crowded regions, and the final fusion stabilizes boundary delineation.

The transition from FPNuNet-AP to the full FPNuNet encompasses four coupled changes: replacing FFT with DCT in both the prompt generators and the frequency-aware fusion modules, upgrading the PFAE block architecture, and introducing the skip-connection enhancement neck. Although the ablation design does not isolate each factor individually, the combined effect constitutes the largest single-step improvement in the sequence (Table 4), consistent with DCT's energy-compaction advantage on localized IHC nuclear textures. A separate internal ablation suggests that the FFT-to-DCT substitution alone yields consistent gains; however, because this intermediate configuration was not included in the

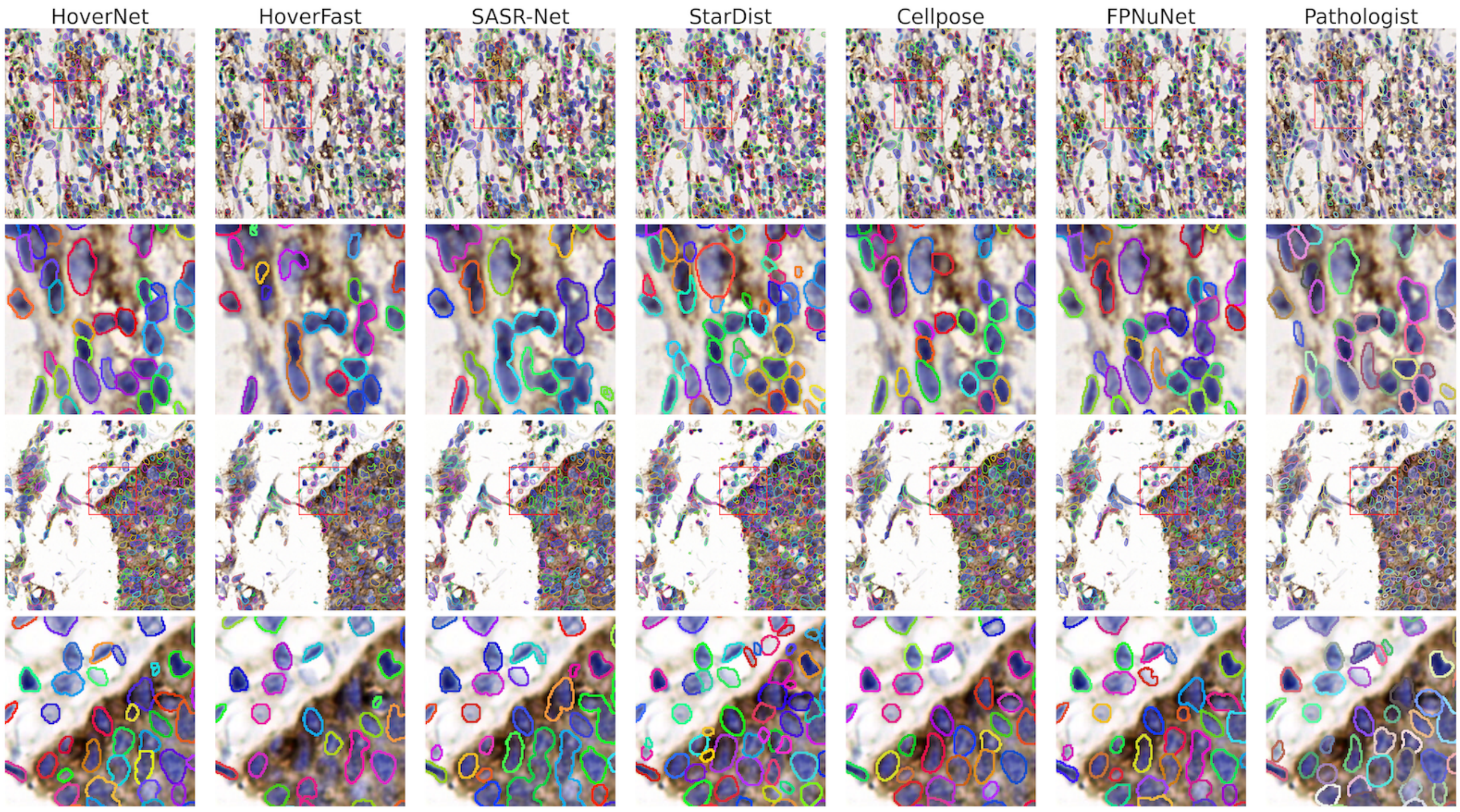


**Figure 7.** Qualitative comparisons of instance segmentation models on representative CD47-IHCNuSC test samples. Colors are used solely to distinguish individual nuclei.

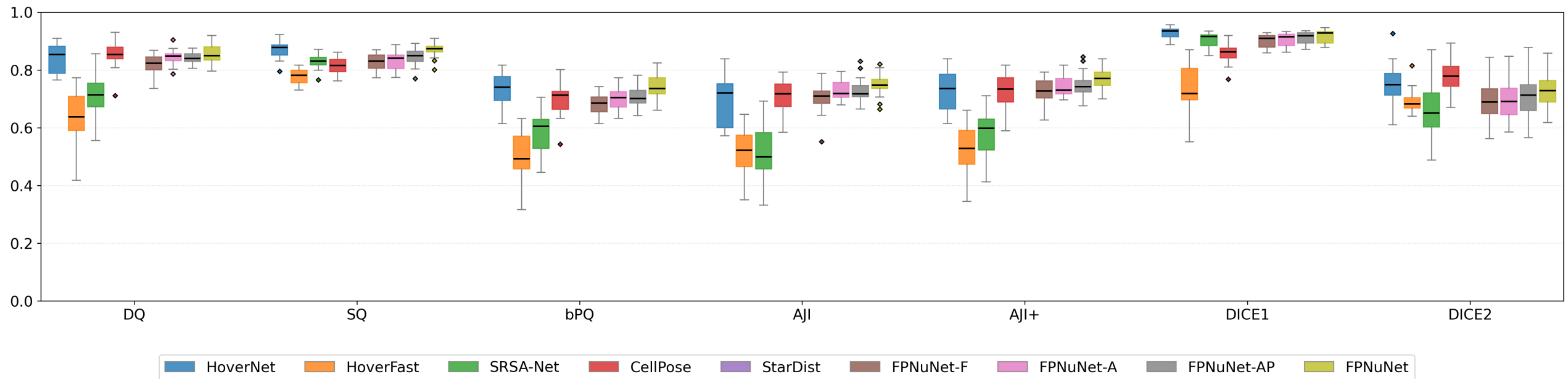


**Figure 8.** Box plots highlighting the performance of competing instance segmentation methods on the CD47-IHCNuSC dataset. Each box summarizes the distribution of evaluation metrics (*DQ*, *SQ*, *bPQ*, *AJI*, *AJI+*, *Dice*, and *Dice2*) across all test images.

**Table 3.** Quantitative evaluation of instance segmentation on the CD47-IHCNuSC test set. Best result per metric in **bold**.

| Model | Dice | Dice2 | AJI | AJI+ | bPQ | DQ | SQ | $F_{1,inst}$ | $P_{inst}$ | $R_{inst}$ |
|---|---|---|---|---|---|---|---|---|---|---|
| HoVer-Net | **0.9264** | 0.7541 | 0.6962 | 0.7307 | 0.7303 | 0.8387 | **0.8700** | 0.8438 | 0.8647 | 0.8264 |
| SRSA-Net | 0.9051 | 0.6640 | 0.5101 | 0.5822 | 0.5872 | 0.7076 | 0.8292 | 0.7189 | 0.7964 | 0.6588 |
| HoverFast | 0.7403 | 0.6944 | 0.5144 | 0.5256 | 0.5057 | 0.6483 | 0.7785 | 0.6524 | 0.7395 | 0.5903 |
| StarDist | 0.7019 | 0.5751 | 0.4503 | 0.4697 | 0.3529 | 0.4737 | 0.7402 | 0.4753 | 0.3784 | 0.7114 |
| Cellpose-SAM | 0.8585 | **0.7766** | 0.7101 | 0.7263 | 0.6970 | 0.8542 | 0.8147 | 0.8594 | **0.9130** | 0.8147 |
| FPNuNet-F | 0.9017 | 0.6917 | 0.7048 | 0.7256 | 0.6814 | 0.8221 | 0.8286 | 0.8266 | 0.7882 | 0.8723 |
| FPNuNet-A | 0.9055 | 0.6992 | 0.7298 | 0.7443 | 0.7018 | 0.8443 | 0.8310 | 0.8490 | 0.8109 | **0.8921** |
| FPNuNet-AP | 0.9100 | 0.7118 | 0.7303 | 0.7502 | 0.7114 | 0.8431 | 0.8436 | 0.8477 | 0.8122 | 0.8880 |
| FPNuNet | 0.9215 | 0.7296 | **0.7472** | **0.7717** | **0.7458** | **0.8589** | 0.8677 | **0.8637** | 0.8462 | 0.8834 |

main evaluation protocol, we report the combined effect and note the design coupling as a limitation.

Among baselines trained on CD47-IHCNuSC under identical protocols, FPNuNet achieves the strongest instance-segmentation performance (Table 3). Relative to HoVer-Net, FPNuNet improves AJI by +7.3 pp, reflecting primarily recall-driven gains ($\mathbf{R}_{\text{inst}}$: 0.883 vs. 0.826) at a modest precision trade-off ($\mathbf{P}_{\text{inst}}$: 0.846 vs. 0.865), while maintaining comparable shape fidelity (SQ). These gains indicate more complete recovery of occluded and touching nuclei without sacrificing contour quality, as confirmed by tighter interquartile ranges in Fig. 8 and cleaner cluster splits in Fig. 7. Against SRSA-Net, margins are substantially larger (e.g., AJI +23.7 pp), consistent with SRSA-Net's limited recall under dense packing; Fig. 7 further shows residual merges and contour bleeding for SRSA-Net, whereas FPNuNet maintains well-delineated boundaries.

Among the instance-only baselines retrained on CD47-IHCNuSC, FPNuNet recorded higher AJI and bPQ than Cellpose-SAM and HoverFast (Table 3); StarDist was evaluated separately

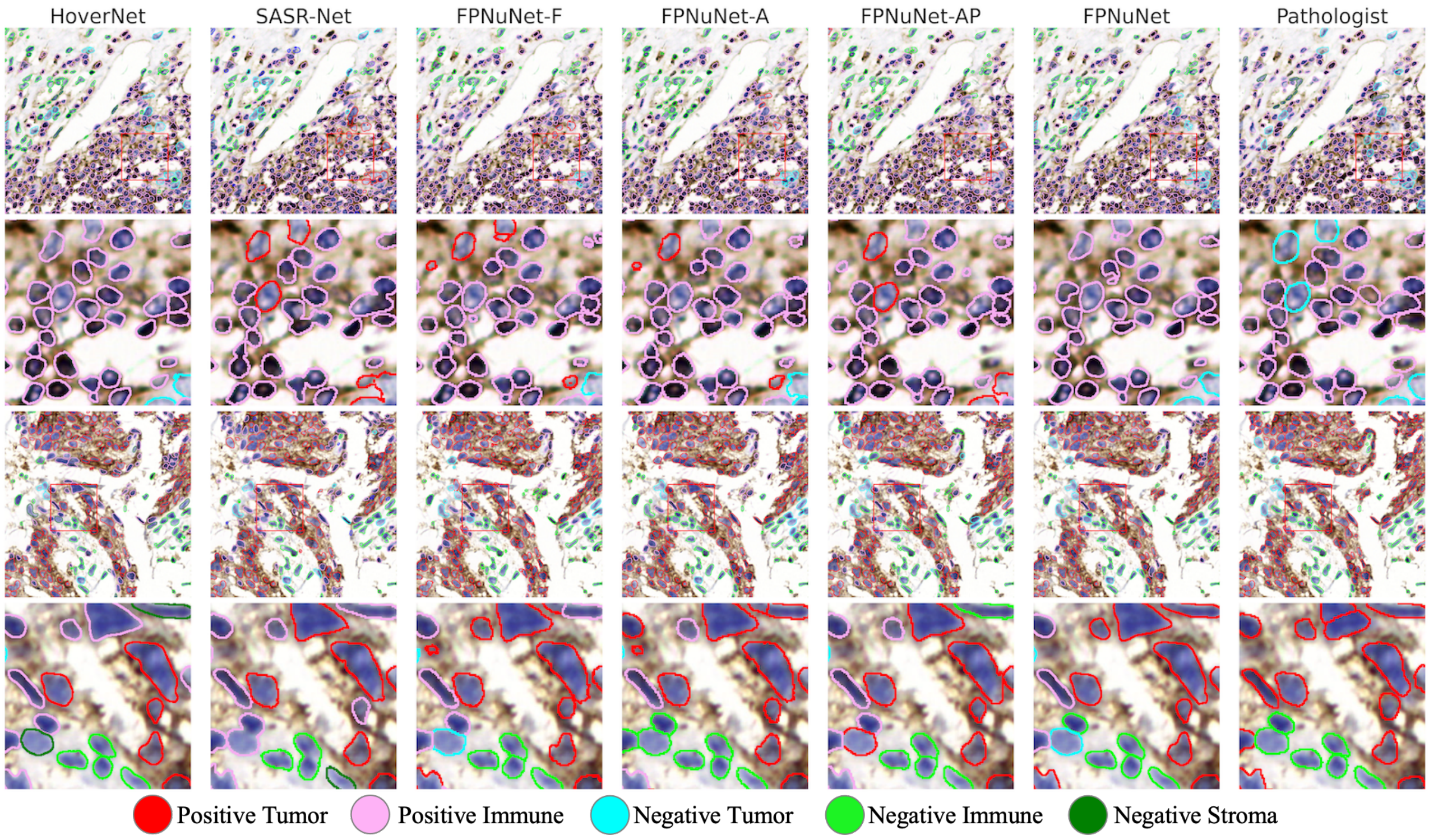


**Figure 9.** Qualitative comparisons of NuSC-capable models on representative CD47-IHCNuSC test samples. All models output instance masks; for models that support classification, subtype predictions are color-coded according to the legend below.

**Table 4.** Ablation study on CD47-IHCNuSC. Each row adds one module to the preceding configuration. Metrics use the same pipeline as Table 3. $\triangle$: pp change vs. previous row.

| Config | Added | AJI | bPQ | DQ | SQ |
|---|---|---|---|---|---|
| FPNuNet-F | Baseline | 0.7048 | 0.6814 | 0.8221 | 0.8286 |
| FPNuNet-A | +Adapter | 0.7298 | 0.7018 | 0.8443 | 0.8310 |
| | $\triangle$ | *+2.50* | *+2.04* | *+2.22* | *+0.24* |
| FPNuNet-AP | +Type Dec. | 0.7303 | 0.7114 | 0.8431 | 0.8436 |
| | $\triangle$ | *+0.05* | *+0.96* | *−0.12* | *+1.26* |
| FPNuNet | +DCT-PFAE | **0.7472** | **0.7458** | **0.8589** | **0.8677** |
| | $\triangle$ | *+1.69* | *+3.44* | *+1.58* | *+2.41* |

**Table 5.** Quantitative evaluation of classification on the CD47-IHCNuSC test set (overall metrics). Best in **bold**.

| Model | mPQ | $P_{macro}$ | $P_w$ | $F_{1,macro}$ | $F_{1,micro}$ | $F_{1,w}$ |
|---|---|---|---|---|---|---|
| HoVer-Net | 0.4801 | 0.6966 | **0.8761** | 0.6349 | 0.7984 | 0.8172 |
| SRSA-Net | 0.3661 | 0.7029 | 0.8648 | 0.5779 | 0.7410 | 0.7668 |
| FPNuNet-F | 0.3664 | 0.5985 | 0.7929 | 0.5028 | 0.7767 | 0.7634 |
| FPNuNet-A | 0.3520 | 0.5534 | 0.7788 | 0.4653 | 0.7647 | 0.7513 |
| FPNuNet-AP | 0.4365 | 0.6888 | 0.8458 | 0.5879 | 0.7906 | 0.8000 |
| FPNuNet | **0.5074** | **0.7097** | 0.8586 | **0.6481** | **0.8239** | **0.8317** |

using its publicly released pretrained weights. Taken together, these results summarize the detection, separation, and contour performance of FPNuNet on CD47-IHCNuSC (Table 3; Figs. 8 and 7).

## Nuclear Type Classification Performance

FPNuNet achieves the strongest aggregate subtype classification performance on CD47-IHCNuSC (Tables 5 and 6; Fig. 9), leading all compared methods on mPQ, $\mathbf{P}_{\text{macro}}$, $\mathbf{F}_{1,\text{macro}}$, $\mathbf{F}_{1,\text{micro}}$, and $\mathbf{F}_{1,w}$. HoVer-Net retains the highest prevalence-weighted precision $\mathbf{P}_w$ (0.8761; FPNuNet: 0.8586), yet FPNuNet's higher macro-averaged precision and $F_1$ point to better cross-class balance rather than reliance on majority categories. Qualitative overlays in Fig. 9 corroborate these trends, with fewer subtype flips at tumor–stroma interfaces and more coherent immune predictions under FPNuNet.

The ablation study within the FPNuNet family quantifies each module's contribution to type recognition. Relative to FPNuNet-F, the adapter-only variant FPNuNet-A degrades both detection-aware type quality (mPQ: 0.3520 vs. 0.3664) and macro-level discrimination, indicating that cross-domain alignment alone does not suffice for phenotype separability. Incorporating the prompt-guided decoder in FPNuNet-AP substantially reverses this trend, lifting mPQ by +24.0% with concurrent improvements across all macro-averaged metrics (Table 5), confirming that explicit type supervision is essential for phenotype discrimination. The complete FPNuNet configuration yields further broad-based gains over FPNuNet-AP, most notably in mPQ (+16.2%) and $\mathbf{F}_{1,\text{macro}}$ (+10.3%), consistent with the stabilizing effect of DCT-based frequency fusion on minority-class decisions. These stepwise gains align with the per-class trends in Table 6: the prompt decoder curbs cross-class confusion in crowded fields (e.g., higher $\mathbf{F}^{pIm}_{1,\text{inst}}$), while the final fusion stabilizes decisions under stain heterogeneity (e.g., higher $\text{PQ}^{nOth}_{\text{inst}}$). Qualitatively, these trends are visible in Fig. 9: adapters reduce missed immune instances at low-contrast membranes, the prompt decoder suppresses residual subtype flips in crowded fields, and the final fusion stabilizes assignments under heterogeneous staining.

These advantages are also evident in the baseline comparison under identical training protocols (Table 5). Although HoVer-Net retains an edge in $\mathbf{P}_w$, this advantage is driven by the prevalent tumor class; FPNuNet's consistently higher macro-averaged metrics indicate more balanced performance across all subtypes, including clinically relevant minority categories.

At the class level (Table 6), FPNuNet and HoVer-Net exhibit a consistent recall–precision trade-off on the dominant pTu

**Table 6.** Per-class classification on the CD47-IHCNuSC test set. All four foreground classes are shown; rare and morphologically ambiguous categories are merged before training (see Section Data Description). Best in **bold**.

| | **pTu** | | | | **pIm** | | | | **nTu** | | | | **nOth** | | | |
|---|---|---|---|---|---|---|---|---|---|---|---|---|---|---|---|---|
| **Model** | PQ | P | R | $F_1$ | PQ | P | R | $F_1$ | PQ | P | R | $F_1$ | PQ | P | R | $F_1$ |
| HoVer-Net | 0.6649 | **0.9496** | 0.8577 | 0.9013 | **0.5564** | 0.7324 | **0.8534** | 0.7883 | 0.3046 | 0.5575 | **0.5808** | 0.5689 | 0.3945 | **0.6547** | 0.6282 | 0.6412 |
| SRSA-Net | 0.5207 | 0.8984 | 0.8395 | 0.8679 | 0.4124 | 0.6640 | 0.8130 | 0.7310 | 0.2385 | 0.5766 | 0.4812 | 0.5246 | 0.2927 | 0.6314 | 0.5533 | 0.5897 |
| FPNuNet-F | 0.6157 | 0.8724 | **0.9353** | 0.9028 | 0.2760 | 0.7344 | 0.7526 | 0.7434 | 0.2291 | 0.5753 | 0.5153 | 0.5437 | 0.3448 | 0.6409 | 0.5505 | 0.5923 |
| FPNuNet-A | 0.6297 | 0.8712 | 0.9171 | 0.8935 | 0.2236 | 0.7439 | 0.7066 | 0.7248 | 0.2313 | 0.5105 | 0.5243 | 0.5173 | 0.3234 | 0.5430 | 0.5459 | 0.5445 |
| FPNuNet-AP | 0.6691 | 0.9098 | 0.8972 | 0.9035 | 0.3657 | 0.7591 | 0.7931 | 0.7758 | 0.3334 | 0.6108 | 0.5667 | 0.5879 | 0.3776 | 0.5649 | 0.6458 | 0.6027 |
| FPNuNet | **0.6894** | 0.9239 | 0.9308 | **0.9273** | 0.5103 | **0.8268** | 0.8344 | **0.8306** | **0.3823** | **0.6623** | 0.5397 | **0.5948** | **0.4476** | 0.6093 | **0.7225** | **0.6611** |

class: FPNuNet achieves higher PQ and $F_1$ through stronger recall, whereas HoVer-Net retains higher precision. For pIm, FPNuNet improves precision and $F_1$ but falls slightly short of HoVer-Net in type-consistent $PQ_{inst}$, suggesting residual assignment sensitivity in crowded lymphocyte clusters. The largest gains emerge on minority subtypes: for nTu, FPNuNet substantially raises precision (0.662 vs. 0.558) and reduces false positives at tumor–non-tumor interfaces; for nOth, markedly improved recall reflects better recovery of diffuse negative non-tumor nuclei. Overall, the gains concentrate on minority and morphologically challenging subtypes (nTu, nOth) while maintaining strong pTu performance, which accounts for the improvements in macro/micro averages and mPQ. HoVer-Net's advantage in $\mathbf{P}_W$ is consistent with its weighting toward the prevalent tumor class. Remaining gaps, particularly in pIm $PQ_{inst}$ (Table 6), indicate headroom in small-object detection and assignment (e.g., class-specific matching thresholds or small-nucleus priors) that could convert improved typing into higher panoptic quality.

We note that paired statistical tests were not applied to the classification metrics because the per-image type-level counts are sparse (several subtypes have fewer than five instances in individual test images), which violates the assumptions of rank-based tests and renders per-image bootstrap intervals unreliable. The reported aggregate differences should therefore be interpreted as descriptive rather than confirmatory.

Finally, the classification gains are consistent with the instance-level improvements reported above. Higher-quality instance masks and improved separation reduce label noise at object boundaries, yielding cleaner per-object features that benefit subtype discrimination. In particular, more accurate boundary localization lessens tumor–stroma contamination and improves immune typing in densely packed regions, as qualitatively illustrated in Fig. 9.

## Frequency Transform Ablation

To isolate the effect of frequency transform selection, we compare four FPNuNet variants that differ only in the transform used in the prompt generators and/or the PFAE fusion modules (Table 7). All variants share the same architecture, training schedule, and hyperparameters; only the frequency transform is varied.

The variant employing DCT in both the prompt generators and the PFAE modules (i.e. the full FPNuNet) achieves the highest AJI (0.7472) and bPQ (0.7458), followed by the variant using FFT uniformly in both stages (AJI = 0.7308) and the baseline without frequency-based prompts (AJI = 0.7136). The variant pairing FFT-based prompt generators with DCT-based PFAE modules attains competitive bPQ (0.7326) but exhibits a notably lower DQ (0.7321 vs. $\geq$0.82 for all other variants), suggesting that mismatched frequency transforms between the prompt and fusion stages may introduce detection instabilities under CD47-specific stain heterogeneity.

Removing the frequency-based prompt yields the lowest AJI, confirming that input-level frequency cues contribute meaningfully to instance recovery. Employing DCT uniformly in both the prompt generation and feature fusion stages achieves the best overall balance, corroborating the design choice adopted in the full FPNuNet model.

**Table 7.** Frequency transform ablation on CD47-IHCNuSC. All variants share the same architecture; only the transform in the prompt generators (Prompt) and PFAE modules is varied. Best in **bold**.

| **Variant (Prompt / PFAE)** | **AJI** | **bPQ** | **DQ** | **SQ** |
|---|---|---|---|---|
| No freq. prompt / DCT | 0.7136 | 0.7221 | 0.8264 | **0.8738** |
| FFT / FFT | 0.7308 | 0.7055 | 0.8214 | 0.8589 |
| FFT / DCT | 0.7249 | 0.7326 | 0.7321 | 0.8681 |
| DCT / DCT (FPNuNet) | **0.7472** | **0.7458** | **0.8589** | 0.8677 |

In summary, FPNuNet achieves overall performance gains on CD47-IHCNuSC by integrating DCT-based frequency-aware refinement, multi-source prompt-adapted feature extraction, and prompt-guided subtype decoding. The ablation results show that these components provide complementary contributions, particularly to subtype classification under challenging IHC staining conditions. These findings suggest that FPNuNet provides a promising framework for IHC-focused nuclear segmentation and classification, with potential utility in tumor cellularity estimation, immune phenotyping, and biomarker-driven pathology analysis.

# Discussion

FPNuNet's performance gains on CD47-IHCNuSC can be attributed to two complementary mechanisms. First, the frozen SAM and UNI backbones, adapted through lightweight DCT-based prompt generators (1.37% trainable parameters), retain generalizable structural and semantic priors while acquiring IHC-specific boundary and texture sensitivity. Second, the supplementary WFE and MSCE branches recover fine-grained spatial and frequency-domain detail that the pretrained encoders lose through repeated downsampling, and the DCT-enabled PFAE fusion neck selectively amplifies morphology-relevant spectral components. The ablation results confirm that these contributions are additive: prompt adaptation improves instance detection, the prompt-guided decoder strengthens subtype discrimination, and DCT-based fusion refines boundary calibration.

CD47-IHCNuSC fills a gap in publicly available IHC benchmarks by coupling biomarker-specific, expert-validated instance annotations with seven fine-grained subtype labels under deliberately preserved staining heterogeneity. Two data-centric factors, however, constrain the conclusions that can be drawn. First, no formal inter-annotator agreement metric (e.g., Cohen's $\kappa$) was recorded during the annotation campaign; residual label noise is therefore unquantified and likely concentrated at morphologically ambiguous boundaries and in rare subtypes, contributing to the performance ceiling on minority classes. Second, the pronounced long-tail class distribution (Table 1) is only partially addressed by the current focal-

loss and background-down-weighting strategy: per-class analysis shows clear gains on nTu and nOth, yet the gap on morphologically challenging subtypes such as pIm remains substantial, suggesting that inverse-frequency weighting, class-balanced sampling, or prototype-based learning for rare categories warrant investigation.

From a computational perspective, FPNuNet trades inference speed for parameter efficiency: although it has the largest total parameter count, only 5.4 M parameters (1.37%) are trainable (Table 2), substantially reducing training memory and optimizer-state overhead. The principal deployment cost is the sequential four-encoder forward pass, which is inherently slower than single-backbone architectures. FPNuNet is therefore best positioned as an empirical validation that frozen foundation models can be effectively adapted to specialized IHC tasks with minimal trainable overhead; lighter encoder configurations can be substituted when deployment latency is the binding constraint.

Several limitations should be acknowledged. The dataset is restricted to the CD47 biomarker in esophageal carcinoma from a single institution, which limits assessment of cross-antibody, cross-organ, and inter-institutional generalizability. External validation was not performed, and no compatible public IHC NuSC benchmark was identified; cross-dataset generalizability therefore remains untested. The test set ($N$=16) further limits statistical power; only large-effect comparisons meet the corrected $p$-value threshold and narrower margins should be interpreted as directional trends. Although the annotations were expert-verified, formal inter-rater agreement was not quantified; residual label ambiguity may therefore affect performance estimates, particularly for morphologically challenging minority classes. The multi-encoder architecture increases memory footprint and latency relative to single-backbone baselines, and end-to-end whole-slide-image throughput—including tile extraction, stitching, and post-processing—was not benchmarked, precluding direct assessment of clinical deployment feasibility.

These limitations suggest several directions for future work. Extending CD47-IHCNuSC to additional biomarkers, organs, and institutions—accompanied by multi-rater annotation with explicit agreement quantification—would strengthen both the benchmark and the evidence base. Domain-adaptive fine-tuning and stain augmentation are promising avenues for improving robustness across diverse IHC staining conditions. At the application level, linking tile- and cell-level outputs to slide-level clinical endpoints such as tumor purity estimation, immune contexture quantification, and biomarker-driven risk stratification would provide a more rigorous assessment of translational utility.

## Availability of Source Code and Requirements

Project name: FPNuNet
Project home page: https://github.com/LuluQin766/FPNuNet
Operating system(s): Linux
Programming language: Python (≥3.7)
Other requirements: PyTorch (≥1.13.0), torchvision (≥0.14.0), PyTorch Lightning, NumPy (≥1.20), SciPy (≥1.10), Pillow (≥9.4), OpenCV (≥4.7), scikit-learn (≥1.2), PyYAML (≥6.0), timm (≥0.3.2), matplotlib (≥3.1), tqdm, tensorboardX, onnxruntime (≥1.14), imageio (≥2.9), h5py
License: MIT License
RRID: SCR_027659
biotools: fpnunet
WorkflowHub: https://doi.org/10.48546/workflowhub.workflow.2166.1

## Data Availability

The CD47-IHCNuSC dataset—including image patches, instance-level masks, nuclear type maps, and metadata—is available in the GigaScience repository, GigaDB [41], under the dataset entry [42]. The DOME-ML annotations for this work can be found in the DOME Registry [43].

## Abbreviations

AJI: aggregated Jaccard index; BCE: binary cross-entropy; bPQ: binary panoptic quality; CBAM: convolutional block attention module; CD47: cluster of differentiation 47; DAB: 3,3′-diaminobenzidine; DQ: detection quality; FFT: fast Fourier transform; FPNuNet: frequency-aware prompt-guided network; H&E: hematoxylin and eosin; HV: horizontal–vertical; IHC: immunohistochemistry; mPQ: mean panoptic quality; MSE: mean squared error; MSGE: mean squared gradient error; NuSC: nuclear segmentation and classification; PFAE: prompt-guided frequency-aware enhancement; PQ: panoptic quality; SAM: Segment Anything Model; SQ: segmentation quality; TMA: Tissue Microarray; UNI: universal pathology foundation model; ViT: Vision Transformer; WSI: whole-slide image.

## Ethics Approval and Consent to Participate

The present study was approved by the Ethics Committee of Jiangjin Central Hospital, Chongqing Municipality (approval no. KY2022037) and was conducted in accordance with all relevant ethical, legal, and institutional regulations. Written informed consent to participate was obtained from all participants.

## Competing interests

The authors declare no competing interests.

## Funding

This work was supported in part by the National Key Research and Development Program of China, under Grant 2022YFF1202104, and in part by the National Natural Science Foundation of China under Grant 62471310.

## Author contributions statement

L.Q.: Conceptualization, Formal analysis, Investigation, Data curation, Writing – original draft, Writing – review & editing. Zh.P.: Conceptualization, Formal analysis, Data curation. X.H.: Conceptualization, Formal analysis. J.Zh.: Conceptualization, Formal analysis, Investigation. Z.Z.: Conceptualization, Formal analysis, Investigation, Writing – original draft, Writing – review & editing. All authors approved the final version.

## References

1. McGenity C, Clarke EL, Jennings C, Matthews G, Cartlidge C, Freduah-Agyemang H, et al. Artificial Intelligence in Digital Pathology: A Systematic Review and Meta-Analysis of Diagnostic Test Accuracy. npj Digital Medicine 2024;7(1):1–19. https://doi.org/10.1038/s41746-024-01106-8.
2. Hosseini MS, Bejnordi BE, Trinh VQH, Chan L, Hasan D, Li X, et al. Computational Pathology: A Survey Review and the Way Forward. Journal of Pathology Informatics 2024;15:100357. https://doi.org/10.1016/j.jpi.2023.100357.
3. Qin L, Yang X, Xu X, Zhu Z. A Survey of Deep Learning in Histopathological Nuclear Segmentation. IEEE Computational

Intelligence Magazine 2025 Aug;20(3):19–40. https://doi.org/10.1109/mci.2025.3564274.
4. Shafi S, Kellough DA, Lujan G, Satturwar S, Parwani AV, Li Z. Integrating and Validating Automated Digital Imaging Analysis of Estrogen Receptor Immunohistochemistry in a Fully Digital Workflow for Clinical Use. Journal of Pathology Informatics 2022;13:100122. https://doi.org/10.1016/j.jpi.2022.100122.
5. Wu S, Yue M, Zhang J, Li X, Li Z, Zhang H, et al. The Role of Artificial Intelligence in Accurate Interpretation of HER2 Immunohistochemical Scores 0 and 1+ in Breast Cancer. Modern Pathology 2023;36(3):100054. https://doi.org/10.1016/j.modpat.2022.100054.
6. Holder AM, Dedeilia A, Sierra-Davidson K, Cohen S, Liu D, Parikh A, et al. Defining clinically useful biomarkers of immune checkpoint inhibitors in solid tumours. Nature Reviews Cancer 2024 Jul;24(7):498–512. https://doi.org/10.1038/s41568-024-00705-7.
7. Graham S, Vu QD, Raza SEA, Azam A, Tsang YW, Kwak JT, et al. Hover-Net: Simultaneous Segmentation and Classification of Nuclei in Multi-Tissue Histology Images. Medical Image Analysis 2019;58:101563. https://doi.org/10.1016/j.media.2019.101563.
8. Chen J, Lu Y, Yu Q, Luo X, Adeli E, Wang Y, et al. TransUNet: Transformers Make Strong Encoders for Medical Image Segmentation. arXiv preprint arXiv:210204306 2021;https://doi.org/10.48550/arxiv.2102.04306.
9. Zhao B, Chen X, Li Z, Yu Z, Yao S, Yan L, et al. Triple U-net: Hematoxylin-aware Nuclei Segmentation with Progressive Dense Feature Aggregation. Medical Image Analysis 2020;65:101786. https://doi.org/10.1016/j.media.2020.101786.
10. Cao H, Wang Y, Chen J, Jiang D, Zhang X, Tian Q, et al. Swin-Unet: Unet-like Pure Transformer for Medical Image Segmentation. In: Computer Vision – ECCV 2022 Workshops Cham: Springer Nature Switzerland; 2023. p. 205–218. https://doi.org/10.1007/978-3-031-25066-8_9.
11. Guan B, Chu G, Wang Z, Li J, Yi B. Instance-level semantic segmentation of nuclei based on multimodal structure encoding. BMC Bioinformatics 2025 Feb;26(1):42. https://doi.org/10.1186/s12859-025-06066-8.
12. Mahanta LB, Hussain E, Das N, Kakoti L, Chowdhury M. IHC-Net: A Fully Convolutional Neural Network for Automated Nuclear Segmentation and Ensemble Classification for Allred Scoring in Breast Pathology. Applied Soft Computing 2021;103:107136. https://doi.org/10.1016/j.asoc.2021.107136.
13. Wang R, Qiu Y, Hao X, Jin S, Gao J, Qi H, et al. Simultaneously segmenting and classifying cell nuclei by using multi-task learning in multiplex immunohistochemical tissue microarray sections. Biomedical Signal Processing and Control 2024 Jul;93:106143. https://doi.org/10.1016/j.bspc.2024.106143.
14. He Z, Jia D, Shi Y, Jiang H, Zhang C, Li Z, et al. An immunohistochemical scoring network based on multi-branch and dual attention mechanisms for the evaluation of biomarker PCNA in esophageal cancer. Applied Soft Computing 2025 Jan;169:112636. https://doi.org/10.1016/j.asoc.2024.112636.
15. Xu S, Li G, Song H, Wang J, Wang Y, Li Q. GeNSeg-Net: A General Segmentation Framework for Any Nucleus in Immunohistochemistry Images. In: Proceedings of the 32nd ACM International Conference on Multimedia New York, NY, USA: Association for Computing Machinery; 2024. p. 4475–4484. https://doi.org/10.1145/3664647.3681441.
16. Wang H, Yang J, Katayama R, Matsusaki M, Miyao T, Zhou J. NuclSeg: nuclei segmentation using semi-supervised stain deconvolution. In: Proceedings of the 5th ACM International Conference on Multimedia in Asia (MMAsia '23) New York, NY, USA: Association for Computing Machinery; 2024. p. 1–6. https://doi.org/10.1145/3595916.3626392.
17. Yao Y, Hu Y, Xue Y, Li S, Huang J, Wang H, et al. UPHGAN: Generative Adversarial Network Based on Unet512 and PatchGAN Fusion with Huber Loss Function for Immunohistochemical Cell Nucleus Segmentation. In: Neural Information Processing (ICONIP 2024), Communications in Computer and Information Science Singapore: Springer Nature; 2025. p. 88–101. https://doi.org/10.1007/978-981-96-6969-1_7.
18. Trullo R, Bui QA, Tang Q, Olfati-Saber R. Image Translation Based Nuclei Segmentation for Immunohistochemistry Images. In: Deep Generative Models Cham: Springer Nature Switzerland; 2022. p. 87–96. https://doi.org/10.1007/978-3-031-18576-2_9.
19. Wang S, Rong R, Gu Z, Fujimoto J, Zhan X, Xie Y, et al. Unsupervised Domain Adaptation for Nuclei Segmentation: Adapting from Hematoxylin & Eosin Stained Slides to Immunohistochemistry Stained Slides Using a Curriculum Approach. Computer Methods and Programs in Biomedicine 2023;241:107768. https://doi.org/10.1016/j.cmpb.2023.107768.
20. Zurek N, Zhang Y, McGovern DPB, Walts AE, Gertych A. Immunohistochemistry annotations enhance AI identification of lymphocytes and neutrophils in digitized H&E slides from inflammatory bowel disease. Computer Methods and Programs in Biomedicine 2024 Dec;257:108423. https://doi.org/10.1016/j.cmpb.2024.108423.
21. Verma R, Kumar N, Patil A, Kurian NC, Rane S, Graham S, et al. MoNuSAC2020: A Multi-Organ Nuclei Segmentation and Classification Challenge. IEEE Transactions on Medical Imaging 2021;40(12):3413–3423. https://doi.org/10.1109/tmi.2021.3085712.
22. Gamper J, Koohbanani NA, Benes K, Graham S, Jahanifar M, Khurram SA, et al. PanNuke Dataset Extension, Insights and Baselines. arXiv preprint arXiv:200310778 2020;https://doi.org/10.48550/arxiv.2003.10778.
23. Wan Ahmad WSHM, Hasan MJ, Fauzi MFA, Lee JTH, Khor SY, Looi LM, et al. Nuclei Classification in ER-IHC Stained Histopathology Images Using Deep Learning Models. In: TENCON 2022 - 2022 IEEE Region 10 Conference (TENCON) Hong Kong, Hong Kong: IEEE; 2022. p. 1–5. https://doi.org/10.1109/tencon55691.2022.9977874.
24. Kirillov A, Mintun E, Ravi N, Mao H, Rolland C, Gustafson L, et al. Segment Anything. In: Proceedings of the IEEE/CVF International Conference on Computer Vision (ICCV); 2023. p. 4015–4026. https://doi.org/10.48550/arxiv.2304.02643.
25. Chen RJ, Ding T, Lu MY, Williamson DFK, Jaume G, Song AH, et al. Towards a general-purpose foundation model for computational pathology. Nature Medicine 2024 Mar;30(3):850–862. https://doi.org/10.1038/s41591-024-02857-3.
26. Zhao HJ, Pa F, Shi YC, Luo X, Ren RR, Peng LH, et al. Prognostic Significance of CD47 in Human Malignancies: A Systematic Review and Meta-Analysis. Translational Cancer Research 2018;7(3). https://doi.org/10.21037/tcr.2018.05.31.
27. Yu L, Ding Y, Wan T, Deng T, Huang H, Liu J. Significance of CD47 and Its Association With Tumor Immune Microenvironment Heterogeneity in Ovarian Cancer. Frontiers in Immunology 2021;12. https://doi.org/10.3389/fimmu.2021.768115.
28. Lee MKI, Rabindranath M, Faust K, Yao J, Gershon A, Alsafwani N, et al. Compound Computer Vision Workflow for Efficient and Automated Immunohistochemical Analysis of Whole Slide Images. Journal of Clinical Pathology 2023;76(7):480–485. https://doi.org/10.1136/jclinpath-2021-208020.
29. Huang Z, Ding Y, Song G, Wang L, Geng R, He H, et al. BC-Data: A Large-Scale Dataset and Benchmark for Cell Detection and Counting. In: Medical Image Computing and Computer Assisted Intervention – MICCAI 2020 Cham: Springer International Publishing; 2020. p. 289–298. https://doi.org/10.1007/978-3-030-59722-1_28.

30. Ghahremani P, Li Y, Kaufman A, Vanguri R, Greenwald N, Angelo M, et al. Deep learning-inferred multiplex immunofluorescence for immunohistochemical image quantification. Nature Machine Intelligence 2022 Apr;4(4):401–412. https://doi.org/10.1038/s42256-022-00471-x.
31. Mouelhi A, Rmili H, Ali JB, Sayadi M, Doghri R, Mrad K. Fast Unsupervised Nuclear Segmentation and Classification Scheme for Automatic Allred Cancer Scoring in Immunohistochemical Breast Tissue Images. Computer Methods and Programs in Biomedicine 2018;165:37–51. https://doi.org/10.1016/j.cmpb.2018.08.005.
32. Liakopoulos P, Massonnet J, Bonjour J, Mizrakli MT, Graham S, Cuendet MA, et al. HoverFast: an accurate, high-throughput, clinically deployable nuclear segmentation tool for brightfield digital pathology images. Journal of Open Source Software 2024 Sep;9(101):7022. https://doi.org/10.21105/joss.07022.
33. Hu EJ, Shen Y, Wallis P, Allen-Zhu Z, Li Y, Wang S, et al. LoRA: Low-Rank Adaptation of Large Language Models. arXiv preprint arXiv:210609685 2022;https://doi.org/10.48550/arXiv.2106.09685.
34. Woo S, Park J, Lee JY, Kweon IS. CBAM: Convolutional Block Attention Module. In: Proceedings of the European Conference on Computer Vision (ECCV); 2018. p. 3–19. https://doi.org/10.48550/arxiv.1807.06521.
35. Weigert M, Schmidt U. Nuclei Instance Segmentation and Classification in Histopathology Images with Stardist. In: 2022 IEEE International Symposium on Biomedical Imaging Challenges (ISBIC); 2022. p. 1–4. https://doi.org/10.1109/isbic56247.2022.9854534.
36. Pachitariu M, Rariden M, Stringer C. Cellpose-SAM: superhuman generalization for cellular segmentation. bioRxiv 2025;p. 2025.04.28.651001. https://doi.org/10.1101/2025.04.28.651001.
37. Taha AA, Hanbury A. Metrics for evaluating 3D medical image segmentation: analysis, selection, and tool. BMC Medical Imaging 2015 Aug;15(1):29. https://doi.org/10.1186/s12880-015-0068-x.
38. Vu QD, Graham S, Kurc T, To MNN, Shaban M, Qaiser T, et al. Methods for Segmentation and Classification of Digital Microscopy Tissue Images. Frontiers in Bioengineering and Biotechnology 2019 Apr;7:53. https://doi.org/10.3389/fbioe.2019.00053.
39. Kumar N, Verma R, Sharma S, Bhargava S, Vahadane A, Sethi A. A Dataset and A Technique for Generalized Nuclear Segmentation for Computational Pathology. IEEE Trans Med Imaging 2017;36(7):1550–1560. https://doi.org/10.1109/TMI.2017.2677499.
40. Kirillov A, He K, Girshick R, Rother C, Dollar P. Panoptic Segmentation. In: Proceedings of the IEEE/CVF Conference on Computer Vision and Pattern Recognition; 2019. p. 9404–9413. https://doi.org/10.1109/CVPR.2019.00963.
41. Li X, Hua C, Yue Q, Li Z, Tong J, Luo Z, et al. GigaDB: a redesigned repository for data publishing and management. GigaScience 2026;15:giag047. https://doi.org/10.1093/gigascience/giag047.
42. Qin L, Pei Z, He X, Zhou J, Xu X, Zhu Z, Supporting data for "FPNuNet: A Frequency-Aware Prompt-Guided Network for Nuclear Segmentation and Classification in Immunohistochemistry Images". GigaScience Database; 2026. https://doi.org/10.5524/102830.
43. Qin L, Pei Z, He X, Zhou J, Xu X, Zhu Z, FPNuNet: A Frequency-Aware Prompt-Guided Network for Nuclear Segmentation and Classification in Immunohistochemistry Images [DOME-ML-Annotations]. DOME-ML Registry; 2026. https://registry.dome-ml.org/review/b21g7cgb5n.
