# Supplementary material for: FPNuNet: a frequency-aware prompt-guided network for nuclear segmentation and classification in immunohistochemistry images
Source: Gigascience. 2026 Sep 7;15:giag091. doi: 10.1093/gigascience/giag091 (PMC13618258; doi:10.1093/gigascience/giag091)
Supplement: Supplementary file 4 [file giag091_reviewer_1_report_original_submission.pdf]

Reviewer Report

Title: FPNuNet: A Frequency-Aware Prompt-Guided Network for Nuclear Segmentation and Classification in Immunohistochemistry Images

Version: Original Submission **Date:** 1/5/2026

Reviewer name: Reviewer 1

Reviewer Comments to Author:

The manuscript proposes FPNuNet, a frequency-aware, prompt-guided dual-encoder network for joint nuclear segmentation and classification in IHC whole-slide imagery, and releases a new expert-verified CD47-IHCNuSC dataset (86 CD47-stained esophageal cancer patches, ~18.5k nuclei, 7 subtypes). The method combines a SAM-based structural encoder with a pathology-pretrained ViT semantic encoder, lightweight prompt adapters, and a progressive frequency-aware fusion neck, and reports improved instance segmentation and type classification over several baselines on the proposed dataset.
Major revision is needed before the claims are fully convincing:
(1) Generalization is under-tested
The dataset is single biomarker (CD47) and single tumor site with a small number of WSIs/patches; add external validation (different cohorts/institutions/scanners and ideally additional IHC markers) and clearly separate within-dataset performance from cross-domain claims;
(2) Evaluation fairness and completeness
Some baselines are run with public pretrained weights without fine-tuning while others are trained under the paper's protocol; provide a controlled comparison (either fine-tune all learnable baselines on CD47-IHCNuSC or clearly restrict conclusions), and include stronger recent IHC nuclei baselines if available;
(3) Reproducibility and data access
The dataset is embargoed/private during review; for a benchmark/dataset paper, reviewers need enough access to verify splits, formats, and scripts; at minimum, provide full preprocessing/tiling/stitching code, exact train/test split identifiers, and evaluation code (plus a clear plan for public release);
(4) Annotation reliability and label noise
The "human-in-the-loop" workflow is described, but quantitative QC (inter-annotator agreement, adjudication rate, error modes for rare classes) is missing; include agreement metrics and an analysis of how class imbalance/rare labels affect results;
(5) Ablation clarity and mechanism
The frequency (DCT/wavelet) and prompt components are complex; add targeted ablations that isolate each contribution under identical settings (and report statistical confidence across slides), plus runtime/memory/latency to justify the added architectural cost.

Methods

Are the methods appropriate to the aims of the study, are they well described, and are necessary controls included? Choose an item.

Conclusions

Are the conclusions adequately supported by the data shown? Choose an item.

Reporting Standards

Does the manuscript adhere to the journal's guidelines on minimum standards of reporting? Choose an item.

Choose an item.

Statistics

Are you able to assess all statistics in the manuscript, including the appropriateness of statistical tests used? Choose an item.

Quality of Written English

Please indicate the quality of language in the manuscript: Choose an item.

Declaration of Competing Interests

Please complete a declaration of competing interests, considering the following questions:

- Have you in the past five years received reimbursements, fees, funding, or salary from an organisation that may in any way gain or lose financially from the publication of this manuscript, either now or in the future?
- Do you hold any stocks or shares in an organisation that may in any way gain or lose financially from the publication of this manuscript, either now or in the future?
- Do you hold or are you currently applying for any patents relating to the content of the manuscript?
- Have you received reimbursements, fees, funding, or salary from an organization that holds or has applied for patents relating to the content of the manuscript?
- Do you have any other financial competing interests?
- Do you have any non-financial competing interests in relation to this paper?

If you can answer no to all of the above, write 'I declare that I have no competing interests' below. If your reply is yes to any, please give details below.

I declare that I have no competing interests

I agree to the open peer review policy of the journal. I understand that my name will be included on my report to the authors and, if the manuscript is accepted for publication, my named report including any attachments I upload will be posted on the website along with the authors' responses. I agree for my

report to be made available under an Open Access Creative Commons CC-BY license (http://creativecommons.org/licenses/by/4.0/). I understand that any comments which I do not wish to be included in my named report can be included as confidential comments to the editors, which will not be published.

Choose an item.

To further support our reviewers, we have joined with Publons, where you can gain additional credit to further highlight your hard work (see: https://publons.com/journal/530/gigascience). On publication of this paper, your review will be automatically added to Publons, you can then choose whether or not to claim your Publons credit. I understand this statement.

Yes Choose an item.
