# Supplementary material for: FPNuNet: a frequency-aware prompt-guided network for nuclear segmentation and classification in immunohistochemistry images
Source: Gigascience. 2026 Sep 7;15:giag091. doi: 10.1093/gigascience/giag091 (PMC13618258; doi:10.1093/gigascience/giag091)
Supplement: Supplementary file 6 [file giag091_reviewer_2_report_original_submission.pdf]

**Reviewer Report**

**Title: FPNuNet: A Frequency-Aware Prompt-Guided Network for Nuclear Segmentation and Classification in Immunohistochemistry Images**

**Version: Original Submission** **Date:** 2/1/2026

**Reviewer name: Reviewer 2**

**Reviewer Comments to Author:**

The authors have presented FPNuNet, a new deep learning architecture that uses a dual-encoder architecture for nuclear segmentation and classification in immunohistochemistry (IHC)-stained images and combines it with a SAM-based structural encoder and a UNI-based semantic encoder along with lightweight prompt adapters and DCT-based frequency-aware fusion modules. The authors have also generated a new dataset of 86 CD47-stained esophageal cancer patches containing 18,494 manually annotated nuclei across seven clinical subtypes.
Currently well-curated and expert verfied IHC datasets lag H&E and IF counterparts. Therefore, CD47-IHCNuSC dataset is indeed a much needed and welcome addition to publicly available datasets. However, I am less enthusiastic about FPNuNet due to the following reasons that I would like the authors to address:
1. The different nuclear categories have severe class imbalance, with for example, 40.5% positive-tumor, while a number of other classes < 1%. While their formulation of loss (for example, focal loss) does help with improving imbalance, there is nothing fundamental about the ability of these losses (which are relatively standard) to mitigate such severe class imbalance. This shows up in the fact that although the authors have curated 7 nuclear categories, they evaluate only 4 of these categories in Table 3. There also does not seem to be any proper discussion of the class-specific weighting that were specifically used to address the dramatic class imbalance. The authors need to clearly discuss the class imbalance, and quantitatively show that their formulation overcomes it in a meaningful manner.
2. I am concerned with the statistical rigor. Specifically, no statistical tests seemed to have been performed, and given only 16 test images, the relative modest improvement in performace could potentially be due to random variation. The authors should improve this aspect of their manuscript.
3. Furthermore, given the relatively modest improvement in performance, the associated increase in complexity is substantial: Dual encoders (SAM + UNI), prompt adapters, multi-scale global encoder (MSGE), wavelet-based frequency extractor (WVTrans), PFAR blocks with DCT, CBAM, coordinate gating, and three decoder branches with cross-attention. The authors do acknowledge this point: "the dualencoder design increases memory footprint and latency relative to single-backbone baseline". However, as there is no quantitative memory and runtime analysis, it is unclear where FPNuNet is a worthwhile direction to go in, even in resource rich settings. The authors themselves mention that focus on single marker limits its assessment of inter-institutional variability and cross-antibody generalizability. From the results presented it is unclear to me whether it would be effectively generalizable. I would urge the authors to quantitatively discuss these aspects in the manuscript. Potentially incorporating validation again other publicly available datasets could help in that effort.

4. The authors state that "The DCT provides real-valued frequency representation without FFT complexity...". However, both FFT and DCT have O(nlogn) complexity so it is unclear to me why DCT and not FFT is used, given that it seems to be that non-DCT architectural changes in FPNuNet are primarily responsible for the improvement. I should note that FFT can potentially be faster in practical implementations because of implementations that are better optimized.
5. Finally, FPNuNet comparisons with models like cellpose did not invovle the latter being fine-tuned at all for the authors' dataset. Only its pre-trained version was used. Even then the performance of FPNuNet is only modestly better. Such comparisons, it seems to me, are not apples to apples comparisons and it would be better if the authors included this fine turning in their comparisons.

**Methods**

Are the methods appropriate to the aims of the study, are they well described, and are necessary controls included? Choose an item.

**Conclusions**

Are the conclusions adequately supported by the data shown? Choose an item.

**Reporting Standards**

Does the manuscript adhere to the journal's guidelines on [minimum standards of reporting?]() Choose an item.

Choose an item.

**Statistics**

Are you able to assess all statistics in the manuscript, including the appropriateness of statistical tests used? Choose an item.

**Quality of Written English**

Please indicate the quality of language in the manuscript: Choose an item.

**Declaration of Competing Interests**

Please complete a declaration of competing interests, considering the following questions:

- Have you in the past five years received reimbursements, fees, funding, or salary from an organisation that may in any way gain or lose financially from the publication of this manuscript, either now or in the future?
- Do you hold any stocks or shares in an organisation that may in any way gain or lose financially from the publication of this manuscript, either now or in the future?

- Do you hold or are you currently applying for any patents relating to the content of the manuscript?
- Have you received reimbursements, fees, funding, or salary from an organization that holds or has applied for patents relating to the content of the manuscript?
- Do you have any other financial competing interests?
- Do you have any non-financial competing interests in relation to this paper?

If you can answer no to all of the above, write 'I declare that I have no competing interests' below. If your reply is yes to any, please give details below.

I declare that I have no competing interests

I agree to the open peer review policy of the journal. I understand that my name will be included on my report to the authors and, if the manuscript is accepted for publication, my named report including any attachments I upload will be posted on the website along with the authors' responses. I agree for my report to be made available under an Open Access Creative Commons CC-BY license (http://creativecommons.org/licenses/by/4.0/). I understand that any comments which I do not wish to be included in my named report can be included as confidential comments to the editors, which will not be published.

Choose an item.

To further support our reviewers, we have joined with Publons, where you can gain additional credit to further highlight your hard work (see: https://publons.com/journal/530/gigascience). On publication of this paper, your review will be automatically added to Publons, you can then choose whether or not to claim your Publons credit. I understand this statement.

Yes Choose an item.
